# HLA diversity in the Argentinian Umbilical Cord Blood Bank: frequencies according to donor’s reported ancestry and geographical distribution

**DOI:** 10.1038/s41598-021-83282-1

**Published:** 2021-02-18

**Authors:** Daniela Fernández Souto, Julieta Rosello, Laura Lazo, Florencia Veloso, Cecilia Gamba, Silvina Kuperman, Valeria Roca

**Affiliations:** 1 Cord Blood Bank, Hemotherapy Regional Center, Hospital de Pediatría Dr.Juan P. Garrahan, Combate de los Pozos 1881, (1245), Buenos Aires, Argentina; 2 National Research Council, Buenos Aires, Argentina

**Keywords:** Genetics, Medical research, Immunology, Transplant immunology

## Abstract

Umbilical cord blood (UCB) is a suitable source for hematopoietic stem cell transplantation. The study of HLA genes by next generation sequencing is commonly used in transplants. Donor/patient HLA matching is often higher within groups of common ancestry, however “Hispanic” is a broad category that fails to represent Argentina’s complex genetic admixture. Our aim is to describe HLA diversity of banked UCB units collected across the country taking into consideration donor’s reported ancestral origins as well as geographic distribution. Our results showed an evenly distribution of units mainly for 2 groups: of European and of Native American descent, each associated to a defined geographic location pattern (Central vs. North regions). We observed differences in allele frequency distributions for some alleles previously described in Amerindian populations: for Class I (A*68:17, A*02:11:01G, A*02:22:01G, B*39:05:01, B*35:21, B*40:04, B*15:04:01G, B*35:04:01, B*51:13:01) and Class II (DRB1*04:11:01, DRB1*04:07:01G/03, DRB1*08:02:01, DRB1*08:07, DRB1*09:01:02G, DRB1*14:02:01, DRB1*16:02:01G). Our database expands the current knowledge of HLA diversity in Argentinian population. Although further studies are necessary to fully comprehend HLA heterogeneity, this report should prove useful to increase the possibility of finding compatible donors for successful allogeneic transplant and to improve recruitment strategies for UCB donors across the country.

## Introduction

Umbilical cord blood (UCB) is considered a suitable source for Hematopoietic stem cell transplantation (HSCT) since 1988 when a patient received cells from an HLA identical sibling^1^. Soon after, unrelated cord blood transplant was shown to be successful and cord blood banks were established worldwide in order to provide alternative sources of hematopoietic progenitor cells (HPC) for patients^2,3^. In 2005, a National Public Cord Blood Bank was created in Argentina to represent the genetic diversity of our country.

Argentina has a population of 44.5 million people located mainly (92%) in urban regions. The majority of Argentinians (97%) are of European descent (mostly from Italy and Spain) and “mestizo” (mixed European and Amerindian ancestry). People of Native American descent compose about 2% of the total population, being mostly found in the North and South of the country. Other non-European groups are also present in our country. Mixed ancestry is also evident in terms of our language. Although Spanish is the national language, a variety of European and Amerindian languages are also spoken by small groups across the nation^4^.

The diversity of cultures is the result of several waves of Colombian and post- Colombian migrations into South America that merged with the ancestral population^5^. Those migrations, in turn, increased HLA genetic diversity. To date, the admixture observed in Argentinian’s genotypes continue to be underspecified.

Since 2005, we have recruited donors from 11 out of 24 provinces. Currently, our bank has over 5.000 cryopreserved UCB units, 3300 of which are available for transplantation and listed through the Argentinian Hematopoietic Progenitor Cell Donor Registry (INCUCAI—Registro Nacional De Donantes De CPH). These units are HLA-A, -B and -DR typed and available internationally, through the Search & Match service from World Marrow Donor Association (WMDA).

The best donor for HSCT is based on a minimal score of high-resolution HLA assignments of HLA-A, -B, -C, and -DRB1. Some transplant centers also consider HLA-DQB1 typing and/or permissive HLA–DPB1 T cell epitopes mismatching^6,7^. Although a fully matched related donor is considered the best option for transplantation, 70% of patients will need an alternative donor and an unrelated HLA-matched donor will be searched as the best source of cells^8^.

Also, a high number of HPC products from unrelated donors are shipped within and across borders worldwide, highlighting the need to understand HLA polymorphisms from different populations in order to lead to the recruitment of the best donor^9^.

Among the parameters of search for a suitable donor, ancestry is usually taken into consideration. Noticeably, international catalogues divide ancestry into population groups, where “Hispanic” is usually a broad category considered for all patients and donors with South and Central American, Hispanic and Latino origins^10,11^. For this reason, it is essential to make an effort to understand and describe the biogeographical heterogeneity of South American populations^12^.

However, to our knowledge, there are only a few previous reports describing high-resolution HLA alleles and haplotype frequencies of UCB units in South America^13^.

Therefore, our aim is to describe HLA diversity of banked UCB units from a random population recruited across the country. We use next generation sequencing (NGS) technology to report HLA allele and haplotype frequencies, taking into consideration donor’s reported ancestral origins and geographic distribution.

## Results

### Demographics characteristics

**Figure 1:**
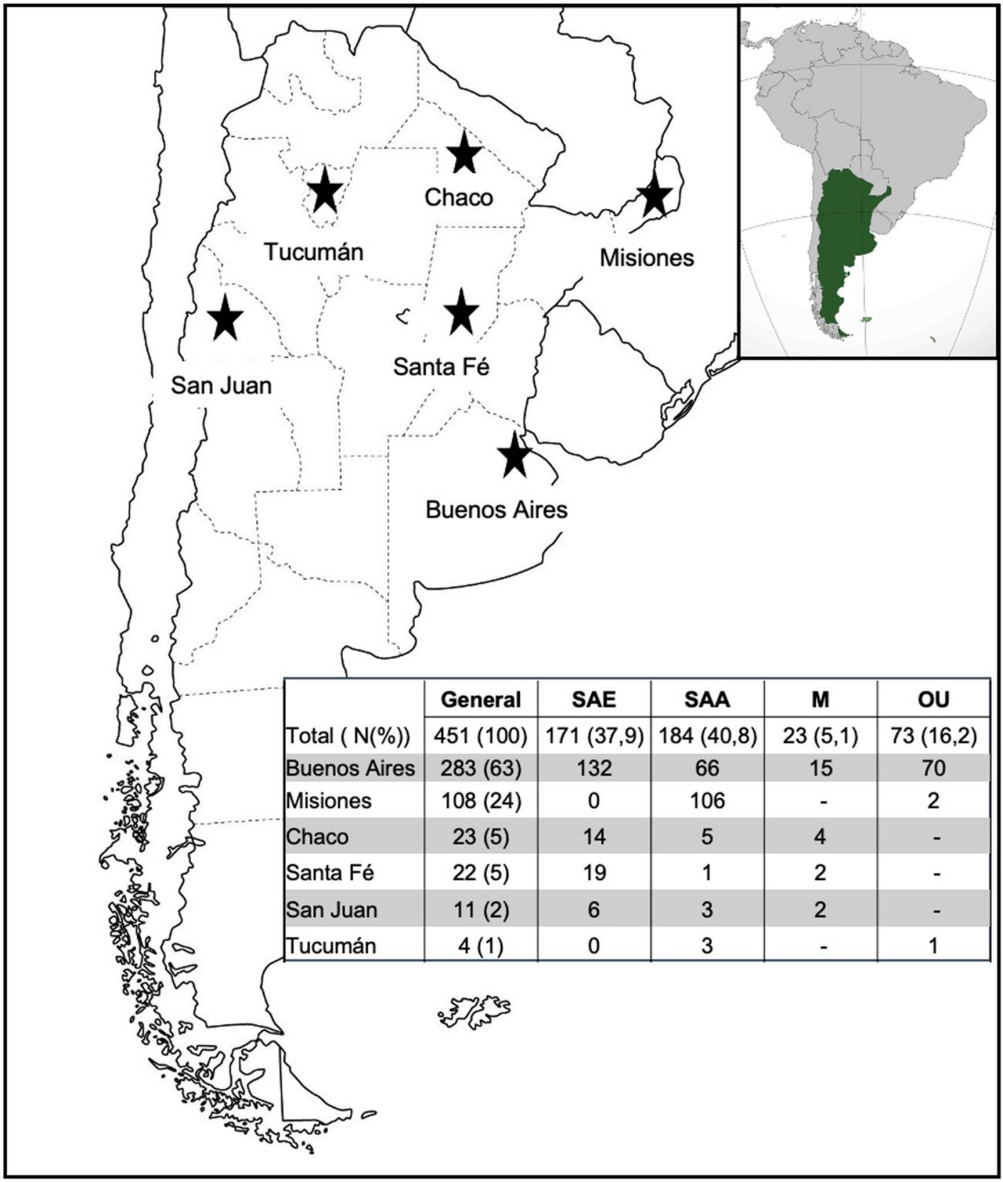
Geographic localization of UCB donations in Argentina and ancestral population groups. Map of Argentina showing the 24 provinces highlighting (stars) the 6 provinces where UCB units were collected. The upper inset shows Argentina´s localization within South America. Table inset shows the number of units collected in each province corresponding to each of the 4 population groups: SAE) South American of European descent, SAA) South American of Amerindian descent, M) Mixed SAE and SAA population, OU) Other mixed or Unknown origins.

UCB units (n = 451) were collected from maternities in 6 of the 24 provinces of Argentina (Fig. 1). Most of the donations (70%) came from the Central region of the country (Buenos Aires, San Juan, Santa Fé) while the remaining 30% were received from the North region (Misiones, Tucumán, Chaco).

Regarding donor´s reported ancestry, UCB units analyzed showed an even distribution between donors born in South America of European descent (SAE, N = 171, 37.9%) and donors born in South America of Amerindians populations descent (SAA, N = 184, 40.8%). However, when the geographic origin is taken into consideration, SAE population is more frequent in the Central region (N = 157/171, 91.8%), whereas SAA population is more frequent in the North (N = 114/184, 61.9%) (Fig. 1, table inset).

### HLA allele frequencies

Detailed information on individual allele frequencies for all loci, in the general population, SAE and SAA groups can be found on Supplementary Table 1.

Forty-two HLA-A alleles appear in our general population of 451 UCB units (Table 1).

**Table 1:** Summary of allele frequency for all loci.

HLA A	General		SAE			SAA		
	2 N = 902		2 N = 342			2 N = 368		
	N	freq	N	Abs freq	Rel freq	N	Abs freq	Rel freq
Total Alleles	42	1.0000	33	0.3792	1.0000	36	0.4080	1.0000
Alleles > 5%	6	0.6375	1	0.0831	0.2193	1	0.0998	0.2446
Other alleles	24	0.3492	21	0.2838	0.7485	23	0.2949	0.7228
One time Allele	12	0.0133	11	0.0122	0.0322	12	0.0133	0.0326
**HLA B**								
Total Alleles	88	1.0000	64	0.3792	1.0000	68	0.4080	1.0000
Alleles > 5%	6	0.3736	0	0.0000	0.0000	0	0.0000	0.0000
Other alleles	59	0.6009	41	0.3537	0.9327	48	0.3858	0.9457
One time Allele	23	0.0255	23	0.0255	0.0673	20	0.0222	0.0543
**HLA C**								
Total Alleles	34	1.0000	23	0.3792	1.0000	30	0.4080	1.0000
Alleles > 5%	8	0.6951	1	0.0599	0.1579	1	0.0621	0.1522
Other alleles	17	0.2949	19	0.3160	0.8333	21	0.3370	0.8261
One time Allele	9	0.0100	3	0.0033	0.0088	8	0.0089	0.0217
**HLA DRB1**								
Total Alleles	47	1.0000	37	0.3792	1.0000	38	0.4080	1.0000
Alleles > 5%	5	0.3525	0	0.0000	0.0000	0	0.0000	0.0000
Other alleles	33	0.6375	31	0.3725	0.9825	35	0.4047	0.9918
One time Allele	9	0.0100	6	0.0067	0.0175	3	0.0033	0.0082
**HLA DQB1**								
Total Alleles	17	1.0000	14	0.3792	1.0000	15	0.4080	1.0000
Alleles > 5%	8	0.8869	4	0.2616	0.6901	3	0.2361	0.5788
Other alleles	6	0.1098	8	0.1153	0.3041	10	0.1696	0.4158
One time Allele	3	0.0033	2	0.0022	0.0058	2	0.0022	0.0054

For the General population (451 UCB units, 902 alleles) N: Number of alleles present, Freq: frequency. For SAE and SAA populations (171 UCB units, 342 alleles and 184 UCB units, 368 alleles respectively) N: Number of alleles present, Abs Freq: absolute frequency, Rel Freq: relative frequency within the population.

Six alleles, each present at least 45 times (5%), contribute to 63.7% of the total allele frequency. Two alleles, A*02:01:01G (216/902, 23.9%) and A*24:02:01G (91/902, 10.1%), exhibit frequencies over 10% (Suppl. Table 1). Twelve HLA-A alleles appear only once and account for 1.3% of the total allele frequency. The analysis within SAE and SAA groups showed 33 and 36 different alleles respectively; only 1 allele in each population was present at least 45 times. Further analysis at low resolution showed that A*68 and A*31 were more represented in SAA group: A*68, SAA = 34 versus SAE = 14 and A*31, SAA = 32 versus SAE = 19. Conversely, A*26 was found 16 times in SAE group and 8 times in SAA group (Table 2). Noticeably, alleles previously described in our population^14–16^, showed higher frequencies in SAA group: A*68:17 (SAA = 7 versus SAE = 2); A*02:11:01G (SAA = 8 versus SAE = 2); A*02:22:01G (SAA = 1 versus SAE = 0) (Suppl. Table 1).

**Table 2:** Low resolution Class I allele frequency for the general population, SAE and SAA group group.

HLA-A	General		SAE			SAA			HLA-A (cont)	General		SAE			SAA		
	N	A freq	N	A freq	R freq	N	A freq	R freq		N	A freq	N	A freq	R freq	N	A freq	R freq
A*01	82	0.0909	34	0.0377	0.0994	34	0.0377	0.0881	A*30	36	0.0399	15	0.0166	0.0439	13	0.0144	0.0337
A*02	255	0.2827	86	0.0953	0.2515	109	0.1208	0.2824	A*31	61	0.0676	19	0.0211	0.0556	32	0.0355	0.0829
A*03	86	0.0953	35	0.0388	0.1023	32	0.0355	0.0829	A*32	32	0.0355	13	0.0144	0.0380	8	0.0089	0.0207
A*11	45	0.0499	20	0.0222	0.0585	20	0.0222	0.0518	A*33	21	0.0233	10	0.0111	0.0292	8	0.0089	0.0207
A*23	20	0.0222	5	0.0055	0.0146	7	0.0078	0.0181	A*34	1	0.0011	0	–	–	1	0.0011	0.0026
A*24	108	0.1197	49	0.0543	0.1433	37	0.0410	0.0959	A*66	5	0.0055	1	0.0011	0.0029	2	0.0022	0.0052
A*25	15	0.0166	8	0.0089	0.0234	4	0.0044	0.0104	A*68	65	0.0721	14	0.0155	0.0409	34	0.0377	0.0881
A*26	29	0.0322	16	0.0177	0.0468	8	0.0089	0.0207	A*74	1	0.0011	1	0.0011	0.0029	0	–	–
A*29	37	0.0410	14	0.0155	0.0409	18	0.0200	0.0466	A*80	3	0.0033	2	0.0022	0.0058	1	0.0011	0.0026

HLA-B	General		SAE			SAA			HLA-B (cont)	General		SAE			SAA		
	N	A Freq	N	A Freq	R Freq	N	A Freq	R Freq		N	A Freq	N	A Freq	R Freq	N	A Freq	R Freq
B*07	53	0.0588	24	0.0266	0.0702	17	0.0188	0.0440	B*46	1	0.0011	0	–	–	0	–	–
B*08	45	0.0499	14	0.0155	0.0409	21	0.0233	0.0544	B*47	3	0.0033	1	0.0011	0.0029	0	–	–
B*13	16	0.0177	9	0.0100	0.0263	4	0.0044	0.0104	B*48	19	0.0211	5	0.0055	0.0146	9	0.0100	0.0233
B*14	45	0.0499	21	0.0233	0.0614	13	0.0144	0.0337	B*49	12	0.0133	5	0.0055	0.0146	2	0.0022	0.0052
B*15	68	0.0754	14	0.0155	0.0409	39	0.0432	0.1010	B*50	13	0.0144	4	0.0044	0.0117	4	0.0044	0.0104
B*18	59	0.0654	30	0.0333	0.0877	16	0.0177	0.0415	B*51	88	0.0976	39	0.0432	0.1140	35	0.0388	0.0907
B*27	17	0.0188	9	0.0100	0.0263	7	0.0078	0.0181	B*52	11	0.0122	4	0.0044	0.0117	6	0.0067	0.0155
B*35	123	0.1364	42	0.0466	0.1228	55	0.0610	0.1425	B*53	11	0.0122	4	0.0044	0.0117	4	0.0044	0.0104
B*37	5	0.0055	2	0.0022	0.0058	3	0.0033	0.0078	B*55	9	0.0100	7	0.0078	0.0205	1	0.0011	0.0026
B*38	17	0.0188	9	0.0100	0.0263	5	0.0055	0.0130	B*56	3	0.0033	1	0.0011	0.0029	0	–	–
B*39	49	0.0543	16	0.0177	0.0468	25	0.0277	0.0648	B*57	27	0.0299	8	0.0089	0.0234	11	0.0122	0.0285
B*40	61	0.0676	17	0.0188	0.0497	34	0.0377	0.0881	B*58	18	0.0200	3	0.0033	0.0088	10	0.0111	0.0259
B*41	13	0.0144	4	0.0044	0.0117	5	0.0055	0.0130	B*67	1	0.0011	1	0.0011	0.0029	0	–	–
B*44	105	0.1164	46	0.0510	0.1345	37	0.0410	0.0959	B*78	1	0.0011	0	–	–	1	0.0011	0.0026
B*45	8	0.0089	2	0.0022	0.0058	4	0.0044	0.0104	B*81	1	0.0011	1	0.0011	0.0029	0	–	–

HLA-C	General		SAE			SAA			HLA-C (cont)	General		SAE			SAA		
	N	A freq	N	A freq	R freq	N	A freq	R freq		N	A freq	N	A freq	R freq	N	A freq	R freq
C*01	23	0.0255	7	0.0078	0.0205	9	0.0100	0.0233	C*08	55	0.0610	20	0.0222	0.0585	22	0.0244	0.0570
C*02	42	0.0466	24	0.0266	0.0702	13	0.0144	0.0337	C*12	59	0.0654	28	0.0310	0.0819	18	0.0200	0.0466
C*03	108	0.1197	27	0.0299	0.0789	57	0.0632	0.1477	C*14	16	0.0177	6	0.0067	0.0175	4	0.0044	0.0104
C*04	143	0.1585	54	0.0599	0.1579	56	0.0621	0.1451	C*15	56	0.0621	21	0.0233	0.0614	28	0.0310	0.0725
C*05	58	0.0643	22	0.0244	0.0643	19	0.0211	0.0492	C*16	51	0.0565	24	0.0266	0.0702	21	0.0233	0.0544
C*06	70	0.0776	26	0.0288	0.0760	29	0.0322	0.0751	C*17	11	0.0122	4	0.0044	0.0117	4	0.0044	0.0104
C*07	209	0.2317	79	0.0876	0.2310	87	0.0965	0.2254	C*18	1	0.0011	0	–	–	1	0.0011	0.0026

For the General population (451 UCB units, 902 alleles), SAE group (171 UCB units, 342 alleles) and SAA group group (184 UCB units, 368 alleles) N: Number of alleles present, A Freq: absolute frequency, R Freq: relative frequency within each group.

As expected, the highest polymorphism was observed within the HLA-B region, encompassing 88 different alleles (Table 1). The cumulative frequency of HLA-B alleles appearing 45 or more times is 37.3%. Among them, B*51:01:01G (82/902, 9.1%), B*18:01:01G (58/902, 6.4%) and B*44:03:01G (52/902, 5.8%) showed the highest frequencies. Almost one fourth (23/88, 26.1%) of the HLA-B alleles appeared only once accounting for 2.5% of the total frequency. For the SAE group, 64 alleles were found, while one third (23/64, 35.9%) appeared only once. Regarding the SAA group, 68 alleles were found and 20 (20/68, 29.4%) appeared only once. Analyzing the main differences among groups for HLA-B at low resolution, the top 3 alleles families in SAE group were: B*44, B*35, B*51, while in SAA group we found them to be: B*35, B*15, B*44 (Table 2). Remarkably, in fourth place in SAE group we found B*18 (appeared 30 times), while in SAA group it appeared with a lower frequency (16 times). Other alleles showing differences in frequency distribution were B*07 and B*14 (SAE = 24 vs. SAA = 17 and SAE = 21 vs. SAA = 13, respectively). On the other hand, B*15, B*40 and B*58 predominate in SAA group with respect to SAE (B*15, SAA = 39 vs. SAE = 14; B*40, SAA = 34 vs. SAE = 17; and B*58, SAA = 10 vs. SAE = 3). Further analysis of alleles previously described in our population^14,16–22^, showed higher allele frequencies in SAA group: B*39:05:01 and B*40:04 (SAA = 12 vs. SAE = 2 and SAA = 12 vs. SAE = 1 respectively). Moreover, B*35:21 (N = 3); B*15:04:01G (N = 11); B*35:04:01 (N = 10) and B*51:13:01 (N = 2) were only seen in SAA group (Suppl. Table 1).

Thirty-four HLA-C alleles are present in our UCB units. Eight alleles, each present at least 45 times, contribute to 69.5% of the allele frequency. Three alleles, C*04:01:01G (143/902, 15.8%), C*07:01:01G (105/902, 11.6%) and C*07:02:01G (95/902, 10.5%) showed frequencies over 10% (Suppl Table 1). Nine alleles (9/34, 26.5%) appeared only once accounting for 1% of the total frequency. In SAE we found 23 alleles, 3 of which appeared only once, while in SAA, 30 alleles were found and 8 appeared only once. At low resolution, the analysis of the top 3 alleles families showed differences among both groups (SAE: C*07; C*04; C*12 and SAA: C*07; C*03; C*04). Interestingly, C*03 appeared in SAA 57 times, while in SAE it was found only 27 times (Table 2). Likewise, C*12 was found 28 times in SAE group, while only 18 times in SAA group. Other antigens highlighting the differences in frequency between both groups were C*02 and C*15 (SAE = 24 vs. SAA = 13 and SAE = 21 vs. SAA = 28 respectively).

Among Class II genes, forty-seven alleles were defined for HLA-DRB1 (Table 1). Five alleles showed frequencies over 5%, and the cumulative frequency was 35.2%. DRB1*07:01:01G (93/902, 10.3%) is the most frequent allele, followed by DRB1*03:01:01G (61/902, 6.8%) (Suppl Table 1). Nine alleles (9/47, 19.1%) were observed only once accounting for 1% of the total frequency (Table 1). Regarding SAE and SAA populations, a total of 37 and 38 alleles were found, whereas 6 and 3 alleles appeared only once respectively. At low resolution, the main differences were observed in DRB1*01 (SAE = 46 vs. SAA = 23) as well as in DRB1*08 (SAE = 14 vs SAA = 34) (Table 3). Remarkably, among the common alleles previously described in our population^18,20,23–26^, we focused on DRB1*04:11:01 (SAA = 13 vs. SAE = 4); DRB1*04:07:01G/03 (SAA = 10 vs. SAE = 7 and SAA = 4 vs. SAE = 1, respectively); DRB1*08:02:01 (SAA = 16 vs. SAE = 8); DRB1*08:07 (SAA = 8 vs. SAE = 1); DRB1*09:01:02G (SAA = 16 vs. SAE = 5); DRB1*14:02:01 (SAA = 18 vs. SAE = 2) and DRB1*16:02:01G (SAA = 17 vs. SAE = 4) which showed higher allele frequencies in SAA group (Suppl. Table 1).

**Table 3:** Low resolution Class II allele frequency.

HLA-DRB1	General		SAE			SAA		
	N	A freq	N	A freq	R freq	N	A freq	R freq
DRB1*01	83	0.0920	46	0.0510	0.1345	23	0.0255	0.0625
DRB1*03	62	0.0687	19	0.0211	0.0556	27	0.0299	0.0734
DRB1*04	158	0.1752	56	0.0621	0.1637	76	0.0843	0.2065
DRB1*07	93	0.1031	43	0.0477	0.1257	31	0.0344	0.0842
DRB1*08	57	0.0632	14	0.0155	0.0409	34	0.0377	0.0924
DRB1*09	26	0.0288	5	0.0055	0.0146	16	0.0177	0.0435
DRB1*10	8	0.0089	4	0.0044	0.0117	2	0.0022	0.0054
DRB1*11	111	0.1231	51	0.0565	0.1491	40	0.0443	0.1087
DRB1*12	9	0.0100	2	0.0022	0.0058	4	0.0044	0.0109
DRB1*13	119	0.1319	45	0.0499	0.1316	46	0.0510	0.1250
DRB1*14	62	0.0687	16	0.0177	0.0468	28	0.0310	0.0761
DRB1*15	66	0.0732	28	0.0310	0.0819	20	0.0222	0.0543
DRB1*16	48	0.0532	13	0.0144	0.0380	21	0.0233	0.0571

HLA-DQB1	General		SAE			SAA		
	N	A freq	N	A freq	R freq	N	A freq	R freq
DQB1*02	140	0.1552	56	0.0621	0.1637	54	0.0599	0.1467
DQB1*03	408	0.4523	143	0.1585	0.4181	182	0.2018	0.4946
DQB1*04	50	0.0554	13	0.0144	0.0380	30	0.0333	0.0815
DQB1*05	152	0.1685	75	0.0831	0.2193	44	0.0488	0.1196
DQB1*06	152	0.1685	55	0.0610	0.1608	58	0.0643	0.1576

Low resolution allele frequency for the general population (451 UCB units, 902 alleles), SAE group group (171 UCB units, 342 alleles) and SAA group group (184 UCB units, 368 alleles) N: Number of alleles present, A Freq: absolute frequency, R Freq: relative frequency within each group.

Seventeen HLA-DQB1 alleles are present in our population. Eight alleles, each present at least 45 times, make up for 88.7% of the total frequency (Table 1). Three alleles have frequencies over 10%: DQB1*03:01:01G (220/902, 24.4%), DQB1*02:01:01G (140/902, 15.5%), DQB1*03:02:01G (138/902, 15.3%) (Suppl Table 1). Three alleles (3/17, 17.6%) appeared only once accounting for 0.3% of the total frequency. Remarkably, DQB1*05 was represented 75 times in SAE population and 44 times in SAA population (Table 3).

### Common, intermediate and well documented alleles

In order to further study the alleles found in our samples, we classified them according to the CIWD catalogue^10^. The majority of the alleles detected at each locus are common (> 80% for all loci) (Table 4).

**Table 4:** Common, intermediate and well-documented alleles.

Loci	Total alleles (N)	Common alleles N (%)	Other alleles	Allele	Referred ancestry
HLA-A	42	34 (81%)	Intermediate	02:13	SAE
				02:33	SAA,OU
				24:02:02	OU
				31:02	SAA
			Well documented	01:104	SAE
				03:08	SAA
				24:175	SAA
				68:23	SAE
HLA-B	88	74 (84%)	Intermediate	15:20	SAA,SAE
				15:27:02	OU
				35:06	SAA
				35:21	SAA,OU
				39:11	M
				39:13:01	SAA
				39:14	SAA,SAE
				48:02:01	SAA,SAE,M, OU
				51:13:01	SAA
				67:01:02G	SAE
			Well documented	08:33	OU
				15:70	OU
				49:18:02	SAE
				51:04	SAA
HLA-C	34	28 (82%)	Intermediate	02:14:01G	SAA
				05:09:01	SAA
			Well documented	06:30	M
				07:206	SAA
				15:03	SAA
				15:08	OU
HLA-DRB1	47	43 (91%)	Intermediate	04:05:04	SAA
				04:07:03	SAA, SAE
				16:07	SAE
			Well documented	14:02:02	OU
HLA-DQB1	17	15 (88%)	Intermediate	06:11:01	OU
			Well documented	04:02:03	SAA

Alleles were classified according to CIWD version 3.0.0. and assigned to one of four frequency categories: common (≥ 1 in 10 000), intermediate (≥ 1 in 100 000), well-documented (≥ 5 occurrences) or not-CIWD. Referred ancestry according to our categories: SAE; SAA; M (SAE and SAA); OU.

For HLA-A, 4 intermediate alleles were found: A*02:13; A*02:33; A*24:02:02, and A*31:02, whereas 2 intermediate HLA-C alleles were observed: C*02:14:01G and C*05:09:01. Ten HLA-B alleles were classified as intermediate. For Class II alleles, HLA-DRB1 and HLA-DQB1, 3 and 1 intermediate alleles were found respectively.

Well documented (WD) alleles were observed in all loci. Notably, A*01:104, known for Asian Pacific Islander population (API) within the CIWD catalogue, was observed in SAE group, and A*03:08, observed in European (EURO) and African (AFA) populations according to the catalogue, was spotted in SAA group. Also, A*68:23 and A*24:175 catalogued for Hispanic (HIS) and EURO populations were seen in our SAE and SAA group respectively.

Regarding HLA-B WD alleles, B*08:33, and B*15:70, both catalogue for HIS and EURO populations, were seen in OU (other mixed or unknown origin). Also, B*49:18:02 known for being present in HIS population was found in our SAE group, whereas B*51:04 known to be in many populations (HIS, EURO, AFA, and Middle East North Coast of Africa: MENA) was seen in our SAA group.

Most of HLA-C well documented alleles found in our cohort are known for EURO and HIS populations, and notably spotted in SAA group (C*07:206, C*15:03), in OU group (C*15:08) and in SAE and SAA Mixed (M) group (C*06:30).

Within Class II group, only one well documented allele was found for HLA-DRB1 and HLA-DQB1 respectively. DRB1*14:02:02 known for EURO and HIS ancestry was seen in OU and DQB1*04:02:03 known for EURO and API ancestry, was spotted in SAA group.

### Hardy Weinberg equilibrium and linkage disequilibrium

Genotype frequencies of HLA-A, -B, -C, -DRB1, -DQB1 loci did not deviate from Hardy–Weinberg equilibrium (HWE) expectations in any of the groups (Suppl. Table 2). Strong linkage disequilibrium (LD) was confirmed between class I, HLA-A, -B, and -C, as well as class II, HLA-DRB1 and -DQB1 loci (Suppl. Table 3).

### Haplotype frequencies

A complete list of predicted five locus haplotypes is given in Supplementary Tables 4–6. A total of 10872 HLA-A~C~B~DRB1~DQB1 haplotypes were estimated in our general population (Suppl. Table 4), with 655 haplotypes accounting for almost 100% cumulative frequency. The list of 15 most frequent allelic combinations (> 5%) is available in Table 5.

**Table 5:** List of the more frequent estimated HLA-A~B~C~DRB1~DQB1 haplotypes for the general population.

General population						
Pos	Freq	Haplotype				
1	0.01663	A*01:01:01G	C*07:01:01G	B*08:01:01G	DRB1*03:01:01G	DQB1*02:01:01G
2	0.01545	A*29:02:01G	C*16:01:01G	B*44:03:01G	DRB1*07:01:01G	DQB1*02:01:01G
3	0.01330	A*03:01:01G	C*07:02:01G	B*07:02:01G	DRB1*15:01:01G	DQB1*06:02:01G
4	0.00887	A*30:02:01G	C*05:01:01G	B*18:01:01G	DRB1*03:01:01G	DQB1*02:01:01G
5	0.00776	A*02:01:01G	C*07:02:01G	B*07:02:01G	DRB1*15:01:01G	DQB1*06:02:01G
6	0.00776	A*02:01:01G	C*15:02:01G	B*51:01:01G	DRB1*11:01:01G	DQB1*03:01:01G
7	0.00769	A*02:01:01G	C*05:01:01G	B*44:02:01G	DRB1*13:01:01G	DQB1*06:03:01G
8	0.00665	A*02:01:01G	C*05:01:01G	B*44:02:01G	DRB1*01:01:01G	DQB1*05:01:01G
9	0.00665	A*24:02:01G	C*12:03:01G	B*18:01:01G	DRB1*11:04:01G	DQB1*03:01:01G
10	0.00665	A*33:01:01G	C*08:02:01G	B*14:02:01G	DRB1*01:02:01	DQB1*05:01:01G
11	0.00660	A*03:01:01G	C*04:01:01G	B*35:01:01G	DRB1*01:01:01G	DQB1*05:01:01G
12	0.00554	A*02:01:01G	C*03:04:01G	B*40:01:01G	DRB1*13:02:01G	DQB1*06:04:01G
13	0.00554	A*24:02:01G	C*07:01:01G	B*08:01:01G	DRB1*03:01:01G	DQB1*02:01:01G
14	0.00554	A*24:02:01G	C*04:01:01G	B*35:02:01G	DRB1*11:04:01G	DQB1*03:01:01G
15	0.00554	A*30:01:01G	C*06:02:01G	B*13:02:01G	DRB1*07:01:01G	DQB1*02:01:01G

Regarding SAE group, a total of 4337 haplotypes were estimated; 277 of which accounted for a cumulative frequency of almost 100% (Suppl. Table 5).

For SAA group a total of 4522 haplotypes were estimated, where 299 accounted for almost 100% frequency (Suppl. Table 6).

Further analysis showed that most of the estimated haplotypes had been reported previously in our population (27). Notably, we observed one haplotype combination, in group SAE, position 13 (frequency: 0.00585), that includes an odd LD for HLA-DRB1~HLA-DQB1 (HLA-A*01:01:01G~C*08:02:01G~B*14:01:01~DRB1*15:01:01G~DQB1*02:01:01G). Detailed analysis of the units containing this LD showed that others LD for HLA-DRB1~HLA-DQB1 are more likely to be present, since they have been previously reported (Suppl. Table 7)^27–33^.

## Discussion

This is the first report on high-resolution HLA diversity on Argentinian cord blood units. Also, it is the first report on Argentina’s population analyzing allele and haplotype frequencies taking into consideration donors reported ancestral origins.

As previously described, different ancestral origins coexist within Argentina’s borders, mainly from European descent (especially from Italy and Spain) and from Native American tribes (Mapuches, Kollas, etc.) but also from Africa, Asia. Moreover, this diversity is remarkable in terms of geographic distribution where groups of Amerindian descent tend to live in the Northern and Southern regions of the country. Therefore, our analysis included the general population, but also a sub-study to account for these differences. Our results showed that, out of 451 UCB units analyzed, SAE group (of European descent) and SAA group (of Native American descent) were both evenly distributed (SAE = 171 vs. SAA = 184, Fig. 1). As we expected, geographical distribution was also noticeable, as SAE group was more frequent in the Central region and SAA group was more frequent in the North region.

To a great extent, our results, regarding allele frequency for all loci and haplotypes estimations, are in accordance with the previous reports on Argentinian bone marrow donors by the National Registry^34,35^. Nonetheless, numerous differences were found between SAE and SAA groups.

Among the common alleles, A*68:17 has been previously described and frequently found in our population. A*68:17 was first identified in Kolla Amerindians of North West Argentina^14,15^. At low resolution, A*68 was present 65 times (SAA = 34 vs. SAE = 14, Table 2), within this allele group, A*68:17 was seen 21.5% (14 times, Suppl. Table 1) (including two units homozygous for this allele). Remarkably, when ancestral origin is considered, A*68:17 appeared 7 times in SAA group and only 2 times in SAE group. Further analyzing these units, we noticed that it usually appeared in combination with alleles in locus B also described in our populations, such us B*35:19 (and C*08:01:01); B*35:21; B*39:14; B*35:05:01; B*35:06; B*48:03:01 among others.

Other alleles that also showed differences among both groups are: A*02:11:01G (founded 8 times in SAA group and 2 times in SAE group) and A*02:22:01G (appearing in SAA group) (see Suppl. Table 1)^15,17^.

Regarding HLA-B locus, B*39:05:01, an allele commonly found in Amerindian populations from Mexico to Argentina^17–19^, appeared 12 times in SAA group and only 2 times in SAE group (Suppl. Table 1). Other alleles, such as B*35:21, B*40:04, B*15:04:01G, B*35:04:01, previously described in South Western Brazil and North Western Argentina populations^16,20,21^, were also seen more frequently in SAA group. Based on our experience, B*51:13:01^14,22^, which appeared twice in SAA group, should be highlighted as well.

Analyzing HLA-DR locus, 3 alleles previously described in Argentinian Amerindian populations caught our attention: DRB1*08:02:01, DRB1*08:07 and DRB1*04:11:01^18,20,23–25^. DRB1*08:02:01 was spotted 16 times in SAA group and 8 times in SAE group, whereas DRB1*08:07 was seen 8 times within SAA group and only once in SAE group. For DRB1*04:11:01, we detected it 20 times in our population, 13 times in SAA group and only 4 times in SAE group (see Suppl. Table 1). Noticeably, alleles such as DRB1*04:07:01G/03; DRB1*09:01:02G; DRB1*14:02:01; DRB1*16:02:01G also had a marked predominance in SAA group in comparison to SAE group^20,24–26^.

Finally, as we analyzed the haplotypes estimations for the general population, and for SAE and SAA groups respectively, most of the more frequent estimated haplotypes had already been described in previous works, at the same level of resolution, in bone marrow donors by the Argentinian Registry (Table 5)^34^.

In conclusion, our results showed clear differences in allele frequencies between both groups (SAE vs. SAA). We believe it is important to represent, in our Public Umbilical Cord Blood Bank, all of Argentinians HLA diversity and among it, our ethnic minorities (people born in South America of Amerindians populations) poorly represented in other registries. This lack of representation in worldwide registries, among other challenges, is reflected in the failed searches for unrelated donors for certain patients not only in our population but also for individuals in neighboring countries in South America^36^. Thus, we believe this report will not only provide a better comprehension of the HLA heterogeneity in Argentinian population but also contribute to improve our strategies of recruitment of UCB donors across the country.

## Materials and methods

### Samples

Cryopreserved cord blood units (N = 451) collected between 2015 and 2017, were randomly separated for HLA-A, -B, -C, -DRB1, and -DQB1 genotyping. All units were collected and processed according to the Cord Blood Bank’s Standard Operating Procedures. Informed consent in written form was collected from all donating mothers. Ethical approval for the present protocol was provided by the Ethics Committee of the Hospital de Pediatría Garrahan (No. 1109/2018).

### Population definition

At the time of recruitment, along with the questionnaire to screen for risk of transfusion transmitted diseases, questions regarding the donor’s origin are made.

Considering our mixed ancestry and according to HLA-Net methodological recommendations^37^, 4 groups were defined: SAE (South American of European descent): people born in South America of European descent and/or people born in European countries living in Argentina; SAA (South American of Amerindians populations descent): people born in South America of Amerindians populations descent; M: Mixed population of Amerindians and European populations descent, OU: people referring other ancestries (for example Asian or African), mixed origins (including Amerindian or European with Asian, African or unknown ancestry) or unknown ancestry born or living in Argentina.

### UCB samples and typing

Whole Blood samples with citrate phosphate dextrose solution (CPD) as anticoagulant were sent to LabCorp (Laboratory Corporation of America Holdings, USA) for HLA typing. Labcorp is a contract lab for the National Marrow Donor Program. It is accredited by several agencies, including the American Society for Histocompatibility and Immunogenetics (ASHI) and the College of American Pathologists (CAP).

Samples were analyzed by next generation sequencing as part of a batch of the Argentinian Hematopoietic Progenitor Cell Donor Registry (INCUCAI—Registro Nacional De Donantes De CPH), within the boundaries of government agreements and according to standard protocols.

The results were reported back to INCUCAI with a “G” level resolution.

### Statistics

HLA allele frequencies were calculated for the global cohort, but also separately for the different groups (SAE, SAA, M, OU) by direct counting using Arlequin software 3.5.2.2 (http://cmpg.unibe.ch/software/arlequin35/Arlequin35.html)^38^. Allele frequencies at each HLA locus, deviations from Hardy–Weinberg equilibrium proportions, linkage disequilibrium analysis and five-locus haplotype estimated frequencies were obtained and analyzed using Arlequin software 3.5.2.2. Deviations from Hardy–Weinberg equilibrium were calculated with exact tests using a Markov chain for all Loci for 3 groups (all samples, SAE and SAA). *P* values ≤ 0.05 indicated statistical difference between Observed and Expected Heterozygosity (Obs.Het. vs. Exp.Het.) and thus a deviation from HWE. Linkage disequilibrium analysis was performed between all pairs of loci with unknown gametic phase (significance level < 0.05). We generated five-locus haplotype estimated frequencies (A~C~B~DRB1~DQB1) from the general population, SAE and SAA groups, using the iterative expectation maximization (EM) algorithm (ε = 1e^−7^).

## Supplementary Information

Supplementary Information.
