# Supplementary material for: HLA diversity in the Argentinian Umbilical Cord Blood Bank: frequencies according to donor’s reported ancestry and geographical distribution
Source: Sci Rep. 2021 Feb 18;11:4140. doi: 10.1038/s41598-021-83282-1 (PMC7892815; doi:10.1038/s41598-021-83282-1)
Supplement: Supplementary file 1 — Supplementary Information. [file 41598_2021_83282_MOESM1_ESM.pdf]

HLA diversity in the Argentinean Umbilical Cord Blood Bank: frequencies according to donor's reported ancestry and geographical distribution.

Fernández Souto, Daniela. BS, Cord Blood Bank, Hemotherapy Regional Center, Hospital de Pediatría Dr.Juan P. Garrahan, Buenos Aires, Argentina.

Rosello, Julieta. BS, Cord Blood Bank, Hemotherapy Regional Center, Hospital de Pediatría Dr.Juan P. Garrahan, Buenos Aires, Argentina.

Lazo, Laura. Hematology Tech, Cord Blood Bank, Hemotherapy Regional Center, Hospital de Pediatría Dr.Juan P. Garrahan, Buenos Aires, Argentina.

Veloso, Florencia. Hematology Tech, Cord Blood Bank, Hemotherapy Regional Center, Hospital de Pediatría Dr.Juan P. Garrahan, Buenos Aires, Argentina.

Gamba, Cecilia. MSC. Cord Blood Bank, Hemotherapy Regional Center, Hospital de Pediatría Dr.Juan P. Garrahan, Buenos Aires, Argentina.

Kuperman, Silvina. MD. Cord Blood Bank, Hemotherapy Regional Center, Hospital de Pediatría Dr.Juan P. Garrahan, Buenos Aires, Argentina.

Roca, Valeria* PhD. Cord Blood Bank, Hemotherapy Regional Center, Hospital de Pediatría Dr.Juan P. Garrahan, Buenos Aires, Argentina and National Scientific and Technical Research Council (CONICET), Argentina.

***Corresponding Author:** Roca Valeria, PhD.

Combate de los Pozos 1881, (1245) Buenos Aires, Argentina.

Phone +54 9 11 41226000 ext 6910.

vroca@garrahan.gov.ar

Supplementary Table 1: Allele frequency for all loci

For the General population (451 UCB units, 902 alleles), for SAE group (171 UCB units, 342 alleles) and for SAA group (184 UCB units, 368 alleles) N: Number of alleles present, Abs Freq: Absolute frequency, Rel Freq: Relative Frequency within each group

HLA-A	General (2N=902)		SAE (2N=342)			SAA (2N=368)		
	N	Abs Freq	N	Abs Freq	Rel Freq	N	Abs Freq	Rel Freq
A*01:01:01G	81	0,0898	33	0,0366	0,0965	34	0,0377	0,0924
A*01:104	1	0,0011	1	0,0011	0,0029	0		
A*02:01:01G	216	0,2395	75	0,0831	0,2193	90	0,0998	0,2446
A*02:02:01G	5	0,0055	1	0,0011	0,0029	3	0,0033	0,0082
A*02:04	4	0,0044	1	0,0011	0,0029	3	0,0033	0,0082
A*02:05:01G	10	0,0111	5	0,0055	0,0146	2	0,0022	0,0054
A*02:06:01G	4	0,0044	1	0,0011	0,0029	1	0,0011	0,0027
A*02:11:01G	12	0,0133	2	0,0022	0,0058	8	0,0089	0,0217
A*02:13	1	0,0011	1	0,0011	0,0029	0		
A*02:22:01G	1	0,0011	0			1	0,0011	0,0027
A*02:33	2	0,0022	0			1	0,0011	0,0027
A*03:01:01G	82	0,0909	33	0,0366	0,0965	30	0,0333	0,0815
A*03:02:01G	3	0,0033	2	0,0022	0,0058	1	0,0011	0,0027
A*03:08	1	0,0011	0			1	0,0011	0,0027
A*11:01:01G	45	0,0499	20	0,0222	0,0585	20	0,0222	0,0543
A*23:01:01G	20	0,0222	5	0,0055	0,0146	7	0,0078	0,0190
A*24:02:01G	91	0,1009	44	0,0488	0,1287	28	0,0310	0,0761
A*24:02:02	1	0,0011	0	0,0000	0,0000	0		
A*24:03:01G	15	0,0166	5	0,0055	0,0146	8	0,0089	0,0217
A*24:175	1	0,0011	0			1	0,0011	0,0027
A*25:01:01G	15	0,0166	8	0,0089	0,0234	4	0,0044	0,0109
A*26:01:01G	29	0,0322	16	0,0177	0,0468	8	0,0089	0,0217
A*29:01:01G	2	0,0022	1	0,0011	0,0029	1	0,0011	0,0027
A*29:02:01G	35	0,0388	13	0,0144	0,0380	17	0,0188	0,0462
A*30:01:01G	15	0,0166	8	0,0089	0,0234	5	0,0055	0,0136
A*30:02:01G	20	0,0222	6	0,0067	0,0175	8	0,0089	0,0217
A*30:04:01G	1	0,0011	1	0,0011	0,0029	0	0,0000	0,0000
A*31:01:02G	60	0,0665	19	0,0211	0,0556	31	0,0344	0,0842
A*31:02	1	0,0011	0			1	0,0011	0,0027
A*32:01:01G	32	0,0355	13	0,0144	0,0380	8	0,0089	0,0217
A*33:01:01G	16	0,0177	10	0,0111	0,0292	4	0,0044	0,0109
A*33:03:01G	4	0,0044	0			3	0,0033	0,0082
A*33:05	1	0,0011	0			1	0,0011	0,0027
A*34:02:01	1	0,0011	0			1	0,0011	0,0027
A*66:01:01G	5	0,0055	1	0,0011	0,0029	2	0,0022	0,0054
A*68:01:01G	7	0,0078	4	0,0044	0,0117	1	0,0011	0,0027
A*68:01:02G	31	0,0344	6	0,0067	0,0175	19	0,0211	0,0516
A*68:02:01G	12	0,0133	1	0,0011	0,0029	7	0,0078	0,0190
A*68:17	14	0,0155	2	0,0022	0,0058	7	0,0078	0,0190
A*68:23	1	0,0011	1	0,0011	0,0029	0		
A*74:01:01G	1	0,0011	1	0,0011	0,0029	0		
A*80:01:01G	3	0,0033	2	0,0022	0,0058	1	0,0011	0,0027

HLA-B	General (2N=902)		SAE (2N=342)			SAA (2N=368)		
	N	Freq	N	Abs Freq	Rel Freq	N	Abs Freq	Rel Freq
B*07:02:01G	49	0,0543	23	0,0255	0,0673	15	0,0166	0,0408
B*07:05:01G	4	0,0044	1	0,0011	0,0029	2	0,0022	0,0054
B*08:01:01G	44	0,0488	14	0,0155	0,0409	21	0,0233	0,0571
B*08:33	1	0,0011	0			0		
B*13:02:01G	16	0,0177	9	0,0100	0,0263	4	0,0044	0,0109
B*14:01:01	13	0,0144	8	0,0089	0,0234	3	0,0033	0,0082
B*14:02:01G	32	0,0355	13	0,0144	0,0380	10	0,0111	0,0272
B*15:01:01G	30	0,0333	7	0,0078	0,0205	18	0,0200	0,0489
B*15:03:01G	2	0,0022	0			1	0,0011	0,0027
B*15:04:01G	16	0,0177	0			11	0,0122	0,0299
B*15:07:01G	1	0,0011	0			1	0,0011	0,0027
B*15:08:01	2	0,0022	0			1	0,0011	0,0027
B*15:09	1	0,0011	1	0,0011	0,0029	0		
B*15:10:01	3	0,0033	0			2	0,0022	0,0054
B*15:15	1	0,0011	0			1	0,0011	0,0027
B*15:17:01G	6	0,0067	3	0,0033	0,0088	3	0,0033	0,0082
B*15:18:01G	2	0,0022	2	0,0022	0,0058	0	0,0000	0,0000
B*15:20	2	0,0022	1	0,0011	0,0029	1	0,0011	0,0027
B*15:27:02	1	0,0011	0			0		
B*15:70	1	0,0011	0			0		
B*18:01:01G	58	0,0643	29	0,0322	0,0848	16	0,0177	0,0435
B*18:03	1	0,0011	1	0,0011	0,0029	0	0,0000	0,0000
B*27:02:01	3	0,0033	2	0,0022	0,0058	1	0,0011	0,0027
B*27:05:02G	12	0,0133	5	0,0055	0,0146	6	0,0067	0,0163
B*27:05:03	1	0,0011	1	0,0011	0,0029	0		
B*27:07:01G	1	0,0011	1	0,0011	0,0029	0		
B*35:01:01G	48	0,0532	23	0,0255	0,0673	16	0,0177	0,0435
B*35:02:01G	11	0,0122	5	0,0055	0,0146	3	0,0033	0,0082
B*35:03:01G	9	0,0100	4	0,0044	0,0117	2	0,0022	0,0054
B*35:04:01	11	0,0122	0			10	0,0111	0,0272
B*35:05:01	14	0,0155	1	0,0011	0,0029	11	0,0122	0,0299
B*35:06	2	0,0022	0			1	0,0011	0,0027
B*35:08:01	10	0,0111	3	0,0033	0,0088	5	0,0055	0,0136
B*35:09:01	7	0,0078	3	0,0033	0,0088	1	0,0011	0,0027
B*35:19	3	0,0033	1	0,0011	0,0029	2	0,0022	0,0054
B*35:20:01	4	0,0044	2	0,0022	0,0058	1	0,0011	0,0027
B*35:21	4	0,0044	0			3	0,0033	0,0082
B*37:01:01G	5	0,0055	2	0,0022	0,0058	3	0,0033	0,0082
B*38:01:01	17	0,0188	9	0,0100	0,0263	5	0,0055	0,0136
B*39:01:01G	8	0,0089	4	0,0044	0,0117	3	0,0033	0,0082
B*39:02:02	1	0,0011	1	0,0011	0,0029	0	0,0000	0,0000
B*39:03	11	0,0122	5	0,0055	0,0146	3	0,0033	0,0082
B*39:05:01	15	0,0166	2	0,0022	0,0058	12	0,0133	0,0326
B*39:06:02	3	0,0033	2	0,0022	0,0058	1	0,0011	0,0027
B*39:09:01	5	0,0055	1	0,0011	0,0029	2	0,0022	0,0054
B*39:10:01	1	0,0011	0			1	0,0011	0,0027
B*39:11	1	0,0011	0			0		
B*39:13:01	2	0,0022	0			2	0,0022	0,0054
B*39:14	2	0,0022	1	0,0011	0,0029	1	0,0011	0,0027
B*40:01:01G	23	0,0255	8	0,0089	0,0234	8	0,0089	0,0217
B*40:02:01G	21	0,0233	8	0,0089	0,0234	12	0,0133	0,0326
B*40:04	14	0,0155	1	0,0011	0,0029	12	0,0133	0,0326
B*40:06:01G	1	0,0011	0			1	0,0011	0,0027
B*40:08	2	0,0022	0			1	0,0011	0,0027
B*41:01:01	9	0,0100	3	0,0033	0,0088	3	0,0033	0,0082
B*41:02:01G	4	0,0044	1	0,0011	0,0029	2	0,0022	0,0054

B*44:02:01G	48	0,0532	17	0,0188	0,0497	18	0,0200	0,0489
B*44:03:01G	52	0,0576	26	0,0288	0,0760	18	0,0200	0,0489
B*44:03:02G	1	0,0011	1	0,0011	0,0029	0		
B*44:04	2	0,0022	0			1	0,0011	0,0027
B*44:05:01	2	0,0022	2	0,0022	0,0058	0		0,0000
B*45:01:01G	8	0,0089	2	0,0022	0,0058	4	0,0044	0,0109
B*46:01:01G	1	0,0011	0			0		
B*47:01:01G	3	0,0033	1	0,0011	0,0029	0		
B*48:01:01G	10	0,0111	3	0,0033	0,0088	5	0,0055	0,0136
B*48:02:01	8	0,0089	2	0,0022	0,0058	4	0,0044	0,0109
B*48:03:01	1	0,0011	0			0		
B*49:01:01G	11	0,0122	4	0,0044	0,0117	2	0,0022	0,0054
B*49:18:02	1	0,0011	1	0,0011	0,0029	0		
B*50:01:01G	12	0,0133	3	0,0033	0,0088	4	0,0044	0,0109
B*50:02	1	0,0011	1	0,0011	0,0029	0	0,0000	0,0000
B*51:01:01G	82	0,0909	38	0,0421	0,1111	30	0,0333	0,0815
B*51:04	1	0,0011	0			1	0,0011	0,0027
B*51:07:01	1	0,0011	0			1	0,0011	0,0027
B*51:08:01	2	0,0022	1	0,0011	0,0029	1	0,0011	0,0027
B*51:13:01	2	0,0022	0			2	0,0022	0,0054
B*52:01:01G	7	0,0078	2	0,0022	0,0058	4	0,0044	0,0109
B*52:01:02	4	0,0044	2	0,0022	0,0058	2	0,0022	0,0054
B*53:01:01G	11	0,0122	4	0,0044	0,0117	4	0,0044	0,0109
B*55:01:01G	9	0,0100	7	0,0078	0,0205	1	0,0011	0,0027
B*56:01:01G	3	0,0033	1	0,0011	0,0029	0		
B*57:01:01G	22	0,0244	7	0,0078	0,0205	7	0,0078	0,0190
B*57:03:01G	5	0,0055	1	0,0011	0,0029	4	0,0044	0,0109
B*58:01:01G	14	0,0155	2	0,0022	0,0058	7	0,0078	0,0190
B*58:02:01	4	0,0044	1	0,0011	0,0029	3	0,0033	0,0082
B*67:01:02G	1	0,0011	1	0,0011	0,0029	0		
B*78:01:01	1	0,0011	1	0,0011	0,0029	0		
B*81:01:01G	1	0,0011	0			1	0,0011	0,0027

HLA-C	General (2N=902)		SAE (2N=342)			SAA (2N=368)		
	N	Freq	N	Abs Freq	Rel Freq	N	Abs Freq	Rel Freq
C*01:02:01G	23	0,0255	7	0,0078	0,0205	9	0,0100	0,0245
C*02:02:02G	39	0,0432	24	0,0266	0,0702	11	0,0122	0,0299
C*02:10:01G	2	0,0022	0			1	0,0011	0,0027
C*02:14:01G	1	0,0011	0			1	0,0011	0,0027
C*03:02:01G	2	0,0022	0			1	0,0011	0,0027
C*03:03:01G	44	0,0488	14	0,0155	0,0409	21	0,0233	0,0571
C*03:04:01G	58	0,0643	12	0,0133	0,0351	33	0,0366	0,0897
C*03:04:02	3	0,0033	0			2	0,0022	0,0054
C*03:05	1	0,0011	1	0,0011	0,0029	0	0,0000	0,0000
C*04:01:01G	143	0,1585	54	0,0599	0,1579	56	0,0621	0,1522
C*05:01:01G	57	0,0632	22	0,0244	0,0643	18	0,0200	0,0489
C*05:09:01	1	0,0011	0			1	0,0011	0,0027
C*06:02:01G	69	0,0765	26	0,0288	0,0760	29	0,0322	0,0788
C*06:30	1	0,0011	0			0		
C*07:01:01G	105	0,1164	38	0,0421	0,1111	42	0,0466	0,1141
C*07:02:01G	95	0,1053	38	0,0421	0,1111	39	0,0432	0,1060
C*07:04:01G	8	0,0089	3	0,0033	0,0088	5	0,0055	0,0136
C*07:206	1	0,0011	0			1	0,0011	0,0027
C*08:01:01G	10	0,0111	2	0,0022	0,0058	6	0,0067	0,0163
C*08:02:01G	40	0,0443	17	0,0188	0,0497	13	0,0144	0,0353
C*08:03:01G	5	0,0055	1	0,0011	0,0029	3	0,0033	0,0082
C*12:02:01G	7	0,0078	2	0,0022	0,0058	4	0,0044	0,0109
C*12:03:01G	52	0,0576	26	0,0288	0,0760	14	0,0155	0,0380
C*14:02:01G	15	0,0166	6	0,0067	0,0175	4	0,0044	0,0109
C*14:03	1	0,0011	0			0		
C*15:02:01G	48	0,0532	19	0,0211	0,0556	24	0,0266	0,0652
C*15:03	1	0,0011	0			1	0,0011	0,0027
C*15:05:01G	6	0,0067	2	0,0022	0,0058	3	0,0033	0,0082
C*15:08	1	0,0011	0			0		
C*16:01:01G	43	0,0477	20	0,0222	0,0585	18	0,0200	0,0489
C*16:02:01G	5	0,0055	3	0,0033	0,0088	1	0,0011	0,0027
C*16:04:01	3	0,0033	1	0,0011	0,0029	2	0,0022	0,0054
C*17:01:01G	11	0,0122	4	0,0044	0,0117	4	0,0044	0,0109
C*18:01:01G	1	0,0011	0			1	0,0011	0,0027

HLA-DRB1	General (2N=902)		SAE (2N=342)			SAA (2N=368)		
	N	Freq	N	Abs Freq	Rel Freq	N	Abs Freq	Rel Freq
DRB1*01:01:01G	44	0,0488	25	0,0277	0,0731	11	0,0122	0,0299
DRB1*01:02:01	25	0,0277	11	0,0122	0,0322	9	0,0100	0,0245
DRB1*01:03	14	0,0155	10	0,0111	0,0292	3	0,0033	0,0082
DRB1*03:01:01G	61	0,0676	19	0,0211	0,0556	27	0,0299	0,0734
DRB1*03:02:01	1	0,0011	0			0		
DRB1*04:01:01G	19	0,0211	8	0,0089	0,0234	10	0,0111	0,0272
DRB1*04:02:01	22	0,0244	11	0,0122	0,0322	7	0,0078	0,0190
DRB1*04:03:01	21	0,0233	7	0,0078	0,0205	9	0,0100	0,0245
DRB1*04:04:01	35	0,0388	12	0,0133	0,0351	18	0,0200	0,0489
DRB1*04:05:01	10	0,0111	5	0,0055	0,0146	1	0,0011	0,0027
DRB1*04:05:04	1	0,0011	0			1	0,0011	0,0027
DRB1*04:06:01G	1	0,0011	1	0,0011	0,0029	0		
DRB1*04:07:01G	20	0,0222	7	0,0078	0,0205	10	0,0111	0,0272
DRB1*04:07:03	5	0,0055	1	0,0011	0,0029	4	0,0044	0,0109
DRB1*04:08:01	3	0,0033	0			3	0,0033	0,0082
DRB1*04:10:01G	1	0,0011	0			0		
DRB1*04:11:01	20	0,0222	4	0,0044	0,0117	13	0,0144	0,0353
DRB1*07:01:01G	93	0,1031	43	0,0477	0,1257	31	0,0344	0,0842
DRB1*08:01:01G	11	0,0122	3	0,0033	0,0088	5	0,0055	0,0136
DRB1*08:02:01	29	0,0322	8	0,0089	0,0234	16	0,0177	0,0435
DRB1*08:03:02	1	0,0011	0			0		
DRB1*08:04:01	7	0,0078	2	0,0022	0,0058	5	0,0055	0,0136
DRB1*08:07	9	0,0100	1	0,0011	0,0029	8	0,0089	0,0217
DRB1*09:01:02G	26	0,0288	5	0,0055	0,0146	16	0,0177	0,0435
DRB1*10:01:01G	8	0,0089	4	0,0044	0,0117	2	0,0022	0,0054
DRB1*11:01:01G	50	0,0554	24	0,0266	0,0702	16	0,0177	0,0435
DRB1*11:01:02	5	0,0055	0			3	0,0033	0,0082
DRB1*11:02:01	7	0,0078	1	0,0011	0,0029	6	0,0067	0,0163
DRB1*11:03:01	9	0,0100	6	0,0067	0,0175	2	0,0022	0,0054
DRB1*11:04:01G	40	0,0443	20	0,0222	0,0585	13	0,0144	0,0353
DRB1*12:01:01G	9	0,0100	2	0,0022	0,0058	4	0,0044	0,0109
DRB1*13:01:01G	60	0,0665	22	0,0244	0,0643	27	0,0299	0,0734
DRB1*13:02:01G	35	0,0388	10	0,0111	0,0292	15	0,0166	0,0408
DRB1*13:03:01	18	0,0200	9	0,0100	0,0263	4	0,0044	0,0109
DRB1*13:04	1	0,0011	0			0		
DRB1*13:05:01	5	0,0055	4	0,0044	0,0117	0		
DRB1*14:01:01G	28	0,0310	13	0,0144	0,0380	9	0,0100	0,0245
DRB1*14:02:01	30	0,0333	2	0,0022	0,0058	18	0,0200	0,0489
DRB1*14:02:02	1	0,0011	0			0	0,0000	0,0000
DRB1*14:04:01	1	0,0011	0			1	0,0011	0,0027
DRB1*14:06:01	2	0,0022	1	0,0011	0,0029	0		
DRB1*15:01:01G	54	0,0599	26	0,0288	0,0760	14	0,0155	0,0380
DRB1*15:02:01G	8	0,0089	2	0,0022	0,0058	4	0,0044	0,0109
DRB1*15:03:01G	4	0,0044	0			2	0,0022	0,0054
DRB1*16:01:01	21	0,0233	8	0,0089	0,0234	4	0,0044	0,0109
DRB1*16:02:01G	26	0,0288	4	0,0044	0,0117	17	0,0188	0,0462
DRB1*16:07	1	0,0011	1	0,0011	0,0029	0		

HLA-DQB1	General (2N=902)		SAE (2N=342)			SAA (2N=368)		
	N	Freq	N	Abs Freq	Rel Freq	N	Abs Freq	Rel Freq
DQB1*02:01:01G	140	0,1552	56	0,0621	0,1637	54	0,0599	0,1467
DQB1*03:01:01G	220	0,2439	82	0,0909	0,2398	89	0,0987	0,2418
DQB1*03:02:01G	138	0,1530	46	0,0510	0,1345	70	0,0776	0,1902
DQB1*03:03:02G	45	0,0499	14	0,0155	0,0409	22	0,0244	0,0598
DQB1*03:04:01	4	0,0044	1	0,0011	0,0029	1	0,0011	0,0027
DQB1*03:05:01G	1	0,0011	0			0		
DQB1*04:02:01G	49	0,0543	13	0,0144	0,0380	29	0,0322	0,0788
DQB1*04:02:03	1	0,0011	0			1	0,0011	0,0027
DQB1*05:01:01G	97	0,1075	52	0,0576	0,1520	28	0,0310	0,0761
DQB1*05:02:01G	27	0,0299	10	0,0111	0,0292	6	0,0067	0,0163
DQB1*05:03:01G	28	0,0310	13	0,0144	0,0380	10	0,0111	0,0272
DQB1*06:01:01G	8	0,0089	2	0,0022	0,0058	4	0,0044	0,0109
DQB1*06:02:01G	54	0,0599	23	0,0255	0,0673	16	0,0177	0,0435
DQB1*06:03:01G	57	0,0632	21	0,0233	0,0614	24	0,0266	0,0652
DQB1*06:04:01G	25	0,0277	8	0,0089	0,0234	11	0,0122	0,0299
DQB1*06:09:01G	7	0,0078	1	0,0011	0,0029	3	0,0033	0,0082
DQB1*06:11:01	1	0,0011	0			0		

Supplementary Table 2: Hardy Weinberg equilibrium for all loci
Deviations from Hardy Weinberg equilibrium were calculated with exact test using a Markov chain for all Loci. #Genot: number of genotypes, Obs.Het.: Observed heterozygosity, Exp.Het.: Expected Heterozygosity, P- values < 0.05 are considered statistically significant and thus reflect differences between Obs. and Exp. Het., s.d.: standard deviation.

**General Pop.**	#Genot	Obs.Het.	Exp.Het.	P-value	s.d.	Steps done
HLA-A	451	0.87583	0.90218	0.61165	0.00021	1001000
HLA-B	451	0.96231	0.96560	0.65258	0.00012	1001000
HLA-C	451	0.88692	0.92113	0.05660	0.00009	1001000
HLA-DRB1	451	0.95787	0.95715	0.10500	0.00011	1001000
HLA-DQB1	451	0.86031	0.86660	0.22456	0.00024	1001000

**SAE**	#Genot	Obs.Het.	Exp.Het.	P-value	s.d.	Steps done
HLA-A	171	0.89474	0.90422	0.69159	0.00018	1001000
HLA-B	171	0.95906	0.95630	0.22075	0.00007	1001000
HLA-C	171	0.90643	0.91941	0.18921	0.00025	1001000
HLA-DRB1	171	0.97076	0.94910	0.49087	0.00027	1001000
HLA-DQB1	171	0.88304	0.86270	0.97326	0.00014	1001000

**SAA**	#Genot	Obs.Het.	Exp.Het.	P-value	s.d.	Steps done
HLA-A	184	0.86957	0.90260	0.39033	0.00010	1001000
HLA-B	184	0.96196	0.96978	0.61041	0.00010	1001000
HLA-C	184	0.85870	0.92331	0.36152	0.00022	1001000
HLA-DRB1	184	0.94022	0.96002	0.14377	0.00012	1001000
HLA-DQB1	184	0.84783	0.86232	0.12825	0.00019	1001000

Supplementary Table 3: Linkage disequilibrium for all loci
Linkage disequilibrium (LD) analysis was performed between all pairs of loci with unknown gametic phase: + positive LD, - Negative LD (significance level p <0.0500).

**General Pop.**	HLA-A	HLA-B	HLA-C	HLA-DRB1	HLA-DQB1
HLA-A	*	+	+	+	+
HLA-B	+	*	+	+	+
HLA-C	+	+	*	+	+
HLA-DRB1	+	+	+	*	+
HLA-DQB1	+	+	+	+	*

**SAE**	HLA-A	HLA-B	HLA-C	HLA-DRB1	HLA-DQB1
HLA-A	*	+	+	+	-
HLA-B	+	*	+	+	+
HLA-C	+	+	*	+	+
HLA-DRB1	+	+	+	*	+
HLA-DQB1	-	+	+	+	*

**SAA**	HLA-A	HLA-B	HLA-C	HLA-DRB1	HLA-DQB1
HLA-A	*	+	+	+	+
HLA-B	+	*	+	+	+
HLA-C	+	+	*	+	+
HLA-DRB1	+	+	+	*	+
HLA-DQB1	+	+	+	+	*

Supplementary Table 4: Estimated haplotypes and frequencies for the general population.
Five-locus haplotype frequencies (A~C~B~DRB1~DQB1) were estimated for the general population using the iterative expectation maximization (EM) algorithm (ε=1e^{-7}).

Pos	Freq	Haplotype				
1	0,01663	A*01:01:01G	C*07:01:01G	B*08:01:01G	DRB1*03:01:01G	DQB1*02:01:01G
2	0,01545	A*29:02:01G	C*16:01:01G	B*44:03:01G	DRB1*07:01:01G	DQB1*02:01:01G
3	0,01330	A*03:01:01G	C*07:02:01G	B*07:02:01G	DRB1*15:01:01G	DQB1*06:02:01G
4	0,00887	A*30:02:01G	C*05:01:01G	B*18:01:01G	DRB1*03:01:01G	DQB1*02:01:01G
5	0,00776	A*02:01:01G	C*07:02:01G	B*07:02:01G	DRB1*15:01:01G	DQB1*06:02:01G
6	0,00776	A*02:01:01G	C*15:02:01G	B*51:01:01G	DRB1*11:01:01G	DQB1*03:01:01G
7	0,00769	A*02:01:01G	C*05:01:01G	B*44:02:01G	DRB1*13:01:01G	DQB1*06:03:01G
8	0,00665	A*02:01:01G	C*05:01:01G	B*44:02:01G	DRB1*01:01:01G	DQB1*05:01:01G
9	0,00665	A*24:02:01G	C*12:03:01G	B*18:01:01G	DRB1*11:04:01G	DQB1*03:01:01G
10	0,00665	A*33:01:01G	C*08:02:01G	B*14:02:01G	DRB1*01:02:01	DQB1*05:01:01G
11	0,00660	A*03:01:01G	C*04:01:01G	B*35:01:01G	DRB1*01:01:01G	DQB1*05:01:01G
12	0,00554	A*02:01:01G	C*03:04:01G	B*40:01:01G	DRB1*13:02:01G	DQB1*06:04:01G
13	0,00554	A*24:02:01G	C*07:01:01G	B*08:01:01G	DRB1*03:01:01G	DQB1*02:01:01G
14	0,00554	A*24:02:01G	C*04:01:01G	B*35:02:01G	DRB1*11:04:01G	DQB1*03:01:01G
15	0,00554	A*30:01:01G	C*06:02:01G	B*13:02:01G	DRB1*07:01:01G	DQB1*02:01:01G
16	0,00554	A*68:01:02G	C*03:04:01G	B*40:04	DRB1*04:11:01	DQB1*03:02:01G
17	0,00444	A*02:01:01G	C*05:01:01G	B*44:02:01G	DRB1*04:02:01	DQB1*03:02:01G
18	0,00444	A*03:01:01G	C*04:01:01G	B*35:02:01G	DRB1*11:04:01G	DQB1*03:01:01G
19	0,00444	A*24:03:01G	C*03:04:01G	B*40:02:01G	DRB1*16:02:01G	DQB1*03:01:01G
20	0,00444	A*29:02:01G	C*16:01:01G	B*44:03:01G	DRB1*03:01:01G	DQB1*02:01:01G
21	0,00439	A*01:01:01G	C*06:02:01G	B*57:01:01G	DRB1*07:01:01G	DQB1*03:03:02G
22	0,00339	A*29:02:01G	C*16:01:01G	B*44:03:01G	DRB1*13:01:01G	DQB1*06:03:01G
23	0,00333	A*01:01:01G	C*07:01:01G	B*08:01:01G	DRB1*15:01:01G	DQB1*06:02:01G
24	0,00333	A*01:01:01G	C*15:02:01G	B*51:01:01G	DRB1*04:02:01	DQB1*03:02:01G

25	0,00333	A*02:01:01G	C*07:01:01G	B*08:01:01G	DRB1*03:01:01G	DQB1*02:01:01G
26	0,00333	A*02:01:01G	C*01:02:01G	B*15:04:01G	DRB1*14:02:01	DQB1*03:01:01G
27	0,00333	A*02:01:01G	C*03:03:01G	B*15:04:01G	DRB1*16:02:01G	DQB1*03:02:01G
28	0,00333	A*02:01:01G	C*04:01:01G	B*35:01:01G	DRB1*07:01:01G	DQB1*02:01:01G
29	0,00333	A*02:01:01G	C*03:04:01G	B*40:01:01G	DRB1*04:04:01	DQB1*03:02:01G
30	0,00333	A*02:01:01G	C*05:01:01G	B*44:02:01G	DRB1*11:04:01G	DQB1*03:01:01G
31	0,00333	A*02:01:01G	C*06:02:01G	B*57:01:01G	DRB1*07:01:01G	DQB1*03:03:02G
32	0,00333	A*02:04	C*15:02:01G	B*51:01:01G	DRB1*04:11:01	DQB1*04:02:01G
33	0,00333	A*02:11:01G	C*04:01:01G	B*48:02:01	DRB1*04:11:01	DQB1*03:01:01G
34	0,00333	A*02:11:01G	C*04:01:01G	B*48:02:01	DRB1*09:01:02G	DQB1*03:03:02G
35	0,00333	A*03:01:01G	C*08:02:01G	B*14:02:01G	DRB1*13:02:01G	DQB1*06:09:01G
36	0,00333	A*03:01:01G	C*02:02:02G	B*51:01:01G	DRB1*11:01:01G	DQB1*03:01:01G
37	0,00333	A*11:01:01G	C*04:01:01G	B*35:01:01G	DRB1*01:03	DQB1*05:01:01G
38	0,00333	A*24:02:01G	C*07:02:01G	B*39:03	DRB1*04:04:01	DQB1*03:02:01G
39	0,00333	A*24:02:01G	C*06:02:01G	B*50:01:01G	DRB1*07:01:01G	DQB1*02:01:01G
40	0,00333	A*25:01:01G	C*12:03:01G	B*18:01:01G	DRB1*15:01:01G	DQB1*06:02:01G
41	0,00333	A*26:01:01G	C*12:03:01G	B*38:01:01	DRB1*13:01:01G	DQB1*06:03:01G
42	0,00333	A*31:01:02G	C*03:03:01G	B*15:04:01G	DRB1*16:02:01G	DQB1*03:01:01G
43	0,00333	A*31:01:02G	C*04:01:01G	B*35:01:01G	DRB1*01:01:01G	DQB1*05:01:01G
44	0,00333	A*31:01:02G	C*04:01:01G	B*35:04:01	DRB1*08:07	DQB1*04:02:01G
45	0,00333	A*31:01:02G	C*04:01:01G	B*35:20:01	DRB1*16:02:01G	DQB1*03:01:01G
46	0,00333	A*31:01:02G	C*07:02:01G	B*39:03	DRB1*04:04:01	DQB1*03:02:01G
47	0,00333	A*31:01:02G	C*03:04:01G	B*40:01:01G	DRB1*04:04:01	DQB1*03:02:01G
48	0,00333	A*33:01:01G	C*08:02:01G	B*14:02:01G	DRB1*07:01:01G	DQB1*02:01:01G
49	0,00333	A*68:01:02G	C*04:01:01G	B*35:05:01	DRB1*09:01:02G	DQB1*03:03:02G
50	0,00229	A*02:01:01G	C*05:01:01G	B*44:02:01G	DRB1*07:01:01G	DQB1*02:01:01G
51	0,00222	A*01:01:01G	C*07:01:01G	B*08:01:01G	DRB1*08:01:01G	DQB1*04:02:01G

52	0,00222	A*01:01:01G	C*07:01:01G	B*15:17:01G	DRB1*11:01:01G	DQB1*03:01:01G
53	0,00222	A*01:01:01G	C*05:01:01G	B*18:01:01G	DRB1*07:01:01G	DQB1*02:01:01G
54	0,00222	A*01:01:01G	C*06:02:01G	B*37:01:01G	DRB1*10:01:01G	DQB1*05:01:01G
55	0,00222	A*01:01:01G	C*06:02:01G	B*50:01:01G	DRB1*07:01:01G	DQB1*02:01:01G
56	0,00222	A*01:01:01G	C*16:01:01G	B*51:01:01G	DRB1*13:02:01G	DQB1*06:04:01G
57	0,00222	A*01:01:01G	C*06:02:01G	B*57:01:01G	DRB1*07:01:01G	DQB1*02:01:01G
58	0,00222	A*01:01:01G	C*06:02:01G	B*57:01:01G	DRB1*11:01:01G	DQB1*03:01:01G
59	0,00222	A*01:01:01G	C*07:01:01G	B*57:01:01G	DRB1*07:01:01G	DQB1*03:03:02G
60	0,00222	A*02:01:01G	C*07:02:01G	B*07:02:01G	DRB1*08:02:01	DQB1*03:02:01G
61	0,00222	A*02:01:01G	C*07:02:01G	B*07:02:01G	DRB1*12:01:01G	DQB1*03:01:01G
62	0,00222	A*02:01:01G	C*07:01:01G	B*08:01:01G	DRB1*14:01:01G	DQB1*05:03:01G
63	0,00222	A*02:01:01G	C*08:02:01G	B*14:02:01G	DRB1*08:02:01	DQB1*04:02:01G
64	0,00222	A*02:01:01G	C*05:01:01G	B*18:01:01G	DRB1*03:01:01G	DQB1*02:01:01G
65	0,00222	A*02:01:01G	C*07:01:01G	B*18:01:01G	DRB1*13:01:01G	DQB1*06:03:01G
66	0,00222	A*02:01:01G	C*02:02:02G	B*27:05:02G	DRB1*07:01:01G	DQB1*03:03:02G
67	0,00222	A*02:01:01G	C*04:01:01G	B*35:01:01G	DRB1*16:01:01	DQB1*05:02:01G
68	0,00222	A*02:01:01G	C*04:01:01G	B*35:04:01	DRB1*14:02:01	DQB1*03:01:01G
69	0,00222	A*02:01:01G	C*04:01:01G	B*35:05:01	DRB1*04:04:01	DQB1*03:02:01G
70	0,00222	A*02:01:01G	C*04:01:01G	B*35:09:01	DRB1*04:03:01	DQB1*03:02:01G
71	0,00222	A*02:01:01G	C*04:01:01G	B*35:09:01	DRB1*08:02:01	DQB1*03:02:01G
72	0,00222	A*02:01:01G	C*07:02:01G	B*39:05:01	DRB1*04:04:01	DQB1*03:02:01G
73	0,00222	A*02:01:01G	C*07:02:01G	B*39:05:01	DRB1*04:07:03	DQB1*03:02:01G
74	0,00222	A*02:01:01G	C*02:02:02G	B*40:02:01G	DRB1*11:01:01G	DQB1*03:01:01G
75	0,00222	A*02:01:01G	C*03:04:01G	B*40:04	DRB1*04:11:01	DQB1*03:02:01G
76	0,00222	A*02:01:01G	C*17:01:01G	B*41:02:01G	DRB1*13:03:01	DQB1*03:01:01G
77	0,00222	A*02:01:01G	C*05:01:01G	B*44:02:01G	DRB1*04:01:01G	DQB1*03:01:01G
78	0,00222	A*02:01:01G	C*04:01:01G	B*44:03:01G	DRB1*07:01:01G	DQB1*02:01:01G

79	0,00222	A*02:01:01G	C*02:02:02G	B*51:01:01G	DRB1*03:01:01G	DQB1*02:01:01G
80	0,00222	A*02:01:01G	C*03:03:01G	B*51:01:01G	DRB1*04:01:01G	DQB1*03:02:01G
81	0,00222	A*02:01:01G	C*14:02:01G	B*51:01:01G	DRB1*01:01:01G	DQB1*05:01:01G
82	0,00222	A*02:01:01G	C*14:02:01G	B*51:01:01G	DRB1*16:01:01	DQB1*05:02:01G
83	0,00222	A*02:01:01G	C*15:02:01G	B*51:01:01G	DRB1*07:01:01G	DQB1*02:01:01G
84	0,00222	A*02:02:01G	C*17:01:01G	B*41:01:01	DRB1*04:05:01	DQB1*02:01:01G
85	0,00222	A*02:05:01G	C*07:02:01G	B*07:02:01G	DRB1*13:01:01G	DQB1*06:03:01G
86	0,00222	A*02:05:01G	C*06:02:01G	B*50:01:01G	DRB1*09:01:02G	DQB1*03:03:02G
87	0,00222	A*02:11:01G	C*03:04:01G	B*15:01:01G	DRB1*08:04:01	DQB1*04:02:01G
88	0,00222	A*02:33	C*04:01:01G	B*35:21	DRB1*16:02:01G	DQB1*03:01:01G
89	0,00222	A*03:01:01G	C*07:02:01G	B*07:02:01G	DRB1*08:01:01G	DQB1*04:02:01G
90	0,00222	A*03:01:01G	C*12:03:01G	B*18:01:01G	DRB1*04:03:01	DQB1*03:02:01G
91	0,00222	A*03:01:01G	C*01:02:01G	B*27:05:02G	DRB1*01:01:01G	DQB1*05:01:01G
92	0,00222	A*03:01:01G	C*03:03:01G	B*51:01:01G	DRB1*09:01:02G	DQB1*03:03:02G
93	0,00222	A*03:01:01G	C*12:02:01G	B*52:01:01G	DRB1*15:02:01G	DQB1*06:01:01G
94	0,00222	A*03:01:01G	C*04:01:01G	B*53:01:01G	DRB1*07:01:01G	DQB1*02:01:01G
95	0,00222	A*11:01:01G	C*07:01:01G	B*08:01:01G	DRB1*03:01:01G	DQB1*02:01:01G
96	0,00222	A*11:01:01G	C*15:02:01G	B*51:01:01G	DRB1*15:01:01G	DQB1*06:02:01G
97	0,00222	A*23:01:01G	C*06:02:01G	B*45:01:01G	DRB1*11:01:01G	DQB1*03:01:01G
98	0,00222	A*24:02:01G	C*06:02:01G	B*13:02:01G	DRB1*13:03:01	DQB1*03:01:01G
99	0,00222	A*24:02:01G	C*03:03:01G	B*15:01:01G	DRB1*11:03:01	DQB1*03:01:01G
100	0,00222	A*24:02:01G	C*12:03:01G	B*18:01:01G	DRB1*03:01:01G	DQB1*02:01:01G
101	0,00222	A*24:02:01G	C*04:01:01G	B*35:05:01	DRB1*04:04:01	DQB1*03:02:01G
102	0,00222	A*24:02:01G	C*05:01:01G	B*44:02:01G	DRB1*01:01:01G	DQB1*05:01:01G
103	0,00222	A*24:02:01G	C*16:01:01G	B*44:03:01G	DRB1*11:01:01G	DQB1*03:01:01G
104	0,00222	A*24:02:01G	C*15:02:01G	B*51:01:01G	DRB1*13:01:01G	DQB1*06:03:01G
105	0,00222	A*24:02:01G	C*12:02:01G	B*52:01:01G	DRB1*15:02:01G	DQB1*06:01:01G

106	0,00222	A*24:03:01G	C*03:03:01G	B*15:01:01G	DRB1*08:04:01	DQB1*04:02:01G
107	0,00222	A*25:01:01G	C*07:01:01G	B*58:01:01G	DRB1*13:01:01G	DQB1*06:03:01G
108	0,00222	A*26:01:01G	C*08:02:01G	B*14:01:01	DRB1*07:01:01G	DQB1*02:01:01G
109	0,00222	A*26:01:01G	C*16:02:01G	B*51:01:01G	DRB1*07:01:01G	DQB1*02:01:01G
110	0,00222	A*29:02:01G	C*04:01:01G	B*35:01:01G	DRB1*01:01:01G	DQB1*05:01:01G
111	0,00222	A*30:02:01G	C*06:02:01G	B*13:02:01G	DRB1*07:01:01G	DQB1*02:01:01G
112	0,00222	A*31:01:02G	C*01:02:01G	B*07:02:01G	DRB1*04:04:01	DQB1*03:02:01G
113	0,00222	A*31:01:02G	C*03:03:01G	B*15:01:01G	DRB1*08:02:01	DQB1*04:02:01G
114	0,00222	A*31:01:02G	C*04:01:01G	B*35:08:01	DRB1*04:03:01	DQB1*03:02:01G
115	0,00222	A*31:01:02G	C*07:02:01G	B*39:03	DRB1*13:02:01G	DQB1*06:09:01G
116	0,00222	A*31:01:02G	C*03:04:01G	B*40:02:01G	DRB1*04:07:01G	DQB1*03:02:01G
117	0,00222	A*32:01:01G	C*03:03:01G	B*15:01:01G	DRB1*11:01:01G	DQB1*03:01:01G
118	0,00222	A*68:01:02G	C*03:04:01G	B*40:02:01G	DRB1*09:01:02G	DQB1*03:03:02G
119	0,00222	A*68:01:02G	C*03:04:01G	B*40:04	DRB1*04:07:01G	DQB1*03:02:01G
120	0,00222	A*68:17	C*08:01:01G	B*35:19	DRB1*04:03:01	DQB1*03:02:01G
121	0,00222	A*68:17	C*04:01:01G	B*35:21	DRB1*08:02:01	DQB1*04:02:01G
122	0,00222	A*68:17	C*07:02:01G	B*39:14	DRB1*14:02:01	DQB1*03:01:01G
123	0,00116	A*01:01:01G	C*04:01:01G	B*35:01:01G	DRB1*01:01:01G	DQB1*05:01:01G
124	0,00116	A*03:01:01G	C*06:02:01G	B*57:01:01G	DRB1*07:01:01G	DQB1*03:03:02G
125	0,00111	A*01:01:01G	C*04:01:01G	B*07:02:01G	DRB1*08:04:01	DQB1*04:02:01G
126	0,00111	A*01:01:01G	C*15:05:01G	B*07:05:01G	DRB1*15:01:01G	DQB1*06:02:01G
127	0,00111	A*01:01:01G	C*03:02:01G	B*08:01:01G	DRB1*11:02:01	DQB1*03:01:01G
128	0,00111	A*01:01:01G	C*07:01:01G	B*08:01:01G	DRB1*13:01:01G	DQB1*06:03:01G
129	0,00111	A*01:01:01G	C*07:206	B*08:01:01G	DRB1*11:01:02	DQB1*05:02:01G
130	0,00111	A*01:01:01G	C*07:01:01G	B*08:33	DRB1*15:01:01G	DQB1*02:01:01G
131	0,00111	A*01:01:01G	C*08:02:01G	B*14:01:01	DRB1*07:01:01G	DQB1*02:01:01G
132	0,00111	A*01:01:01G	C*12:03:01G	B*14:02:01G	DRB1*04:03:01	DQB1*02:01:01G

133	0,00111	A*01:01:01G	C*07:01:01G	B*15:01:01G	DRB1*13:01:01G	DQB1*06:03:01G
134	0,00111	A*01:01:01G	C*03:03:01G	B*15:17:01G	DRB1*04:07:01G	DQB1*03:03:02G
135	0,00111	A*01:01:01G	C*07:01:01G	B*15:17:01G	DRB1*04:07:03	DQB1*06:04:01G
136	0,00111	A*01:01:01G	C*07:01:01G	B*18:01:01G	DRB1*07:01:01G	DQB1*03:03:02G
137	0,00111	A*01:01:01G	C*12:03:01G	B*18:01:01G	DRB1*03:01:01G	DQB1*02:01:01G
138	0,00111	A*01:01:01G	C*01:02:01G	B*27:05:02G	DRB1*11:01:01G	DQB1*03:01:01G
139	0,00111	A*01:01:01G	C*04:01:01G	B*35:01:01G	DRB1*11:04:01G	DQB1*03:01:01G
140	0,00111	A*01:01:01G	C*07:01:01G	B*35:02:01G	DRB1*11:04:01G	DQB1*03:01:01G
141	0,00111	A*01:01:01G	C*06:02:01G	B*35:05:01	DRB1*04:04:01	DQB1*03:02:01G
142	0,00111	A*01:01:01G	C*04:01:01G	B*35:08:01	DRB1*04:04:01	DQB1*04:02:01G
143	0,00111	A*01:01:01G	C*04:01:01G	B*35:08:01	DRB1*16:07	DQB1*05:02:01G
144	0,00111	A*01:01:01G	C*06:02:01G	B*37:01:01G	DRB1*11:01:01G	DQB1*03:01:01G
145	0,00111	A*01:01:01G	C*06:02:01G	B*37:01:01G	DRB1*11:03:01	DQB1*03:01:01G
146	0,00111	A*01:01:01G	C*07:02:01G	B*39:06:02	DRB1*13:02:01G	DQB1*06:04:01G
147	0,00111	A*01:01:01G	C*03:04:01G	B*40:01:01G	DRB1*04:01:01G	DQB1*03:01:01G
148	0,00111	A*01:01:01G	C*03:04:01G	B*40:04	DRB1*04:11:01	DQB1*03:02:01G
149	0,00111	A*01:01:01G	C*15:02:01G	B*40:06:01G	DRB1*14:04:01	DQB1*05:03:01G
150	0,00111	A*01:01:01G	C*03:04:01G	B*40:08	DRB1*16:02:01G	DQB1*03:01:01G
151	0,00111	A*01:01:01G	C*07:02:01G	B*44:03:01G	DRB1*08:02:01	DQB1*02:01:01G
152	0,00111	A*01:01:01G	C*16:01:01G	B*44:03:01G	DRB1*15:01:01G	DQB1*06:02:01G
153	0,00111	A*01:01:01G	C*06:02:01G	B*45:01:01G	DRB1*04:01:01G	DQB1*03:01:01G
154	0,00111	A*01:01:01G	C*06:02:01G	B*48:01:01G	DRB1*04:02:01	DQB1*04:02:01G
155	0,00111	A*01:01:01G	C*07:01:01G	B*49:01:01G	DRB1*01:03	DQB1*05:01:01G
156	0,00111	A*01:01:01G	C*07:01:01G	B*49:01:01G	DRB1*11:02:01	DQB1*03:01:01G
157	0,00111	A*01:01:01G	C*07:01:01G	B*49:01:01G	DRB1*11:04:01G	DQB1*03:01:01G
158	0,00111	A*01:01:01G	C*06:02:01G	B*50:01:01G	DRB1*11:01:01G	DQB1*03:01:01G
159	0,00111	A*01:01:01G	C*06:02:01G	B*51:01:01G	DRB1*13:01:01G	DQB1*06:03:01G

160	0,00111	A*01:01:01G	C*06:02:01G	B*57:01:01G	DRB1*13:03:01	DQB1*03:01:01G
161	0,00111	A*01:01:01G	C*07:01:01G	B*58:01:01G	DRB1*07:01:01G	DQB1*02:01:01G
162	0,00111	A*01:104	C*07:01:01G	B*18:01:01G	DRB1*11:01:01G	DQB1*03:01:01G
163	0,00111	A*02:01:01G	C*07:02:01G	B*07:02:01G	DRB1*01:03	DQB1*05:01:01G
164	0,00111	A*02:01:01G	C*07:02:01G	B*07:02:01G	DRB1*04:02:01	DQB1*03:02:01G
165	0,00111	A*02:01:01G	C*07:02:01G	B*07:02:01G	DRB1*08:01:01G	DQB1*02:01:01G
166	0,00111	A*02:01:01G	C*15:02:01G	B*07:02:01G	DRB1*04:01:01G	DQB1*03:01:01G
167	0,00111	A*02:01:01G	C*04:01:01G	B*08:01:01G	DRB1*03:01:01G	DQB1*02:01:01G
168	0,00111	A*02:01:01G	C*06:02:01G	B*08:01:01G	DRB1*01:01:01G	DQB1*05:01:01G
169	0,00111	A*02:01:01G	C*07:01:01G	B*08:01:01G	DRB1*16:01:01	DQB1*05:02:01G
170	0,00111	A*02:01:01G	C*06:02:01G	B*13:02:01G	DRB1*11:04:01G	DQB1*03:01:01G
171	0,00111	A*02:01:01G	C*16:04:01	B*13:02:01G	DRB1*10:01:01G	DQB1*05:01:01G
172	0,00111	A*02:01:01G	C*12:03:01G	B*14:01:01	DRB1*07:01:01G	DQB1*02:01:01G
173	0,00111	A*02:01:01G	C*02:02:02G	B*14:02:01G	DRB1*13:01:01G	DQB1*06:03:01G
174	0,00111	A*02:01:01G	C*08:02:01G	B*14:02:01G	DRB1*01:02:01	DQB1*03:01:01G
175	0,00111	A*02:01:01G	C*08:02:01G	B*14:02:01G	DRB1*13:03:01	DQB1*03:01:01G
176	0,00111	A*02:01:01G	C*01:02:01G	B*15:01:01G	DRB1*07:01:01G	DQB1*02:01:01G
177	0,00111	A*02:01:01G	C*03:03:01G	B*15:01:01G	DRB1*07:01:01G	DQB1*02:01:01G
178	0,00111	A*02:01:01G	C*03:03:01G	B*15:01:01G	DRB1*13:01:01G	DQB1*05:01:01G
179	0,00111	A*02:01:01G	C*03:04:01G	B*15:01:01G	DRB1*04:01:01G	DQB1*03:02:01G
180	0,00111	A*02:01:01G	C*04:01:01G	B*15:01:01G	DRB1*08:07	DQB1*04:02:01G
181	0,00111	A*02:01:01G	C*16:01:01G	B*15:01:01G	DRB1*14:01:01G	DQB1*05:03:01G
182	0,00111	A*02:01:01G	C*01:02:01G	B*15:04:01G	DRB1*09:01:02G	DQB1*03:03:02G
183	0,00111	A*02:01:01G	C*03:04:01G	B*15:04:01G	DRB1*14:02:01	DQB1*03:01:01G
184	0,00111	A*02:01:01G	C*04:01:01G	B*15:04:01G	DRB1*14:02:01	DQB1*03:01:01G
185	0,00111	A*02:01:01G	C*07:02:01G	B*15:07:01G	DRB1*03:01:01G	DQB1*02:01:01G
186	0,00111	A*02:01:01G	C*01:02:01G	B*15:09	DRB1*11:01:01G	DQB1*03:01:01G

187	0,00111	A*02:01:01G	C*07:01:01G	B*15:17:01G	DRB1*13:02:01G	DQB1*06:04:01G
188	0,00111	A*02:01:01G	C*05:01:01G	B*18:01:01G	DRB1*11:01:01G	DQB1*03:01:01G
189	0,00111	A*02:01:01G	C*05:01:01G	B*18:01:01G	DRB1*11:02:01	DQB1*03:02:01G
190	0,00111	A*02:01:01G	C*07:01:01G	B*18:01:01G	DRB1*04:01:01G	DQB1*03:01:01G
191	0,00111	A*02:01:01G	C*07:01:01G	B*18:01:01G	DRB1*04:04:01	DQB1*03:02:01G
192	0,00111	A*02:01:01G	C*07:01:01G	B*18:01:01G	DRB1*11:01:01G	DQB1*03:01:01G
193	0,00111	A*02:01:01G	C*07:01:01G	B*18:01:01G	DRB1*12:01:01G	DQB1*03:03:02G
194	0,00111	A*02:01:01G	C*12:03:01G	B*18:01:01G	DRB1*11:04:01G	DQB1*03:01:01G
195	0,00111	A*02:01:01G	C*12:03:01G	B*18:01:01G	DRB1*16:01:01	DQB1*05:02:01G
196	0,00111	A*02:01:01G	C*02:02:02G	B*27:02:01	DRB1*04:01:01G	DQB1*03:02:01G
197	0,00111	A*02:01:01G	C*02:02:02G	B*27:05:03	DRB1*04:04:01	DQB1*03:02:01G
198	0,00111	A*02:01:01G	C*03:04:01G	B*35:01:01G	DRB1*14:02:02	DQB1*03:02:01G
199	0,00111	A*02:01:01G	C*04:01:01G	B*35:01:01G	DRB1*13:01:01G	DQB1*03:03:02G
200	0,00111	A*02:01:01G	C*04:01:01G	B*35:01:01G	DRB1*13:01:01G	DQB1*06:03:01G
201	0,00111	A*02:01:01G	C*04:01:01G	B*35:01:01G	DRB1*16:02:01G	DQB1*03:01:01G
202	0,00111	A*02:01:01G	C*17:01:01G	B*35:01:01G	DRB1*14:01:01G	DQB1*03:01:01G
203	0,00111	A*02:01:01G	C*03:04:01G	B*35:04:01	DRB1*14:02:01	DQB1*03:01:01G
204	0,00111	A*02:01:01G	C*04:01:01G	B*35:04:01	DRB1*04:07:01G	DQB1*03:02:01G
205	0,00111	A*02:01:01G	C*04:01:01G	B*35:05:01	DRB1*08:02:01	DQB1*05:01:01G
206	0,00111	A*02:01:01G	C*04:01:01G	B*35:05:01	DRB1*16:02:01G	DQB1*03:01:01G
207	0,00111	A*02:01:01G	C*04:01:01G	B*35:08:01	DRB1*16:01:01	DQB1*05:02:01G
208	0,00111	A*02:01:01G	C*06:02:01G	B*35:08:01	DRB1*11:04:01G	DQB1*03:01:01G
209	0,00111	A*02:01:01G	C*08:01:01G	B*38:01:01	DRB1*04:07:01G	DQB1*03:02:01G
210	0,00111	A*02:01:01G	C*08:02:01G	B*38:01:01	DRB1*07:01:01G	DQB1*03:02:01G
211	0,00111	A*02:01:01G	C*12:03:01G	B*38:01:01	DRB1*11:01:01G	DQB1*03:01:01G
212	0,00111	A*02:01:01G	C*12:03:01G	B*38:01:01	DRB1*13:01:01G	DQB1*06:03:01G
213	0,00111	A*02:01:01G	C*12:03:01G	B*38:01:01	DRB1*14:01:01G	DQB1*05:03:01G

214	0,00111	A*02:01:01G	C*07:02:01G	B*39:01:01G	DRB1*01:03	DQB1*05:01:01G
215	0,00111	A*02:01:01G	C*12:03:01G	B*39:01:01G	DRB1*03:01:01G	DQB1*02:01:01G
216	0,00111	A*02:01:01G	C*03:04:02	B*39:05:01	DRB1*01:02:01	DQB1*05:01:01G
217	0,00111	A*02:01:01G	C*07:02:01G	B*39:05:01	DRB1*04:03:01	DQB1*03:02:01G
218	0,00111	A*02:01:01G	C*12:03:01G	B*39:06:02	DRB1*16:01:01	DQB1*06:04:01G
219	0,00111	A*02:01:01G	C*07:02:01G	B*39:09:01	DRB1*16:02:01G	DQB1*03:01:01G
220	0,00111	A*02:01:01G	C*07:01:01G	B*39:11	DRB1*14:02:01	DQB1*03:01:01G
221	0,00111	A*02:01:01G	C*07:02:01G	B*39:13:01	DRB1*04:04:01	DQB1*02:01:01G
222	0,00111	A*02:01:01G	C*03:04:01G	B*40:08	DRB1*14:02:01	DQB1*05:01:01G
223	0,00111	A*02:01:01G	C*01:02:01G	B*41:01:01	DRB1*13:02:01G	DQB1*06:04:01G
224	0,00111	A*02:01:01G	C*05:01:01G	B*44:02:01G	DRB1*03:01:01G	DQB1*03:02:01G
225	0,00111	A*02:01:01G	C*05:01:01G	B*44:02:01G	DRB1*04:04:01	DQB1*03:02:01G
226	0,00111	A*02:01:01G	C*05:01:01G	B*44:02:01G	DRB1*09:01:02G	DQB1*03:03:02G
227	0,00111	A*02:01:01G	C*05:01:01G	B*44:02:01G	DRB1*10:01:01G	DQB1*05:01:01G
228	0,00111	A*02:01:01G	C*05:01:01G	B*44:02:01G	DRB1*15:01:01G	DQB1*06:03:01G
229	0,00111	A*02:01:01G	C*05:09:01	B*44:02:01G	DRB1*13:01:01G	DQB1*06:03:01G
230	0,00111	A*02:01:01G	C*06:02:01G	B*44:02:01G	DRB1*11:04:01G	DQB1*03:01:01G
231	0,00111	A*02:01:01G	C*06:02:01G	B*44:02:01G	DRB1*14:01:01G	DQB1*05:03:01G
232	0,00111	A*02:01:01G	C*07:04:01G	B*44:02:01G	DRB1*11:01:01G	DQB1*03:01:01G
233	0,00111	A*02:01:01G	C*06:02:01G	B*44:03:01G	DRB1*07:01:01G	DQB1*02:01:01G
234	0,00111	A*02:01:01G	C*15:02:01G	B*44:03:01G	DRB1*04:04:01	DQB1*03:02:01G
235	0,00111	A*02:01:01G	C*16:01:01G	B*44:03:01G	DRB1*04:03:01	DQB1*03:04:01
236	0,00111	A*02:01:01G	C*16:01:01G	B*44:03:01G	DRB1*07:01:01G	DQB1*02:01:01G
237	0,00111	A*02:01:01G	C*04:01:01G	B*48:01:01G	DRB1*08:02:01	DQB1*03:02:01G
238	0,00111	A*02:01:01G	C*08:01:01G	B*48:01:01G	DRB1*04:05:04	DQB1*06:02:01G
239	0,00111	A*02:01:01G	C*08:01:01G	B*48:01:01G	DRB1*14:02:01	DQB1*03:01:01G
240	0,00111	A*02:01:01G	C*07:01:01G	B*49:01:01G	DRB1*04:05:01	DQB1*03:01:01G

241	0,00111	A*02:01:01G	C*07:01:01G	B*49:01:01G	DRB1*07:01:01G	DQB1*04:02:03
242	0,00111	A*02:01:01G	C*07:02:01G	B*49:01:01G	DRB1*13:02:01G	DQB1*06:04:01G
243	0,00111	A*02:01:01G	C*06:02:01G	B*50:01:01G	DRB1*07:01:01G	DQB1*02:01:01G
244	0,00111	A*02:01:01G	C*02:02:02G	B*51:01:01G	DRB1*08:01:01G	DQB1*04:02:01G
245	0,00111	A*02:01:01G	C*02:02:02G	B*51:01:01G	DRB1*11:01:01G	DQB1*03:01:01G
246	0,00111	A*02:01:01G	C*03:03:01G	B*51:01:01G	DRB1*09:01:02G	DQB1*03:02:01G
247	0,00111	A*02:01:01G	C*04:01:01G	B*51:01:01G	DRB1*01:02:01	DQB1*03:02:01G
248	0,00111	A*02:01:01G	C*07:01:01G	B*51:01:01G	DRB1*13:01:01G	DQB1*06:03:01G
249	0,00111	A*02:01:01G	C*07:01:01G	B*51:01:01G	DRB1*13:03:01	DQB1*02:01:01G
250	0,00111	A*02:01:01G	C*07:04:01G	B*51:01:01G	DRB1*11:01:01G	DQB1*03:01:01G
251	0,00111	A*02:01:01G	C*14:02:01G	B*51:01:01G	DRB1*01:03	DQB1*05:01:01G
252	0,00111	A*02:01:01G	C*14:02:01G	B*51:01:01G	DRB1*08:01:01G	DQB1*04:02:01G
253	0,00111	A*02:01:01G	C*14:02:01G	B*51:01:01G	DRB1*14:01:01G	DQB1*05:03:01G
254	0,00111	A*02:01:01G	C*14:03	B*51:01:01G	DRB1*11:01:02	DQB1*06:02:01G
255	0,00111	A*02:01:01G	C*15:02:01G	B*51:01:01G	DRB1*03:01:01G	DQB1*02:01:01G
256	0,00111	A*02:01:01G	C*16:02:01G	B*51:01:01G	DRB1*11:04:01G	DQB1*03:01:01G
257	0,00111	A*02:01:01G	C*12:03:01G	B*51:04	DRB1*14:01:01G	DQB1*04:02:01G
258	0,00111	A*02:01:01G	C*16:02:01G	B*52:01:02	DRB1*04:11:01	DQB1*03:01:01G
259	0,00111	A*02:01:01G	C*04:01:01G	B*53:01:01G	DRB1*03:01:01G	DQB1*02:01:01G
260	0,00111	A*02:01:01G	C*04:01:01G	B*53:01:01G	DRB1*13:02:01G	DQB1*06:04:01G
261	0,00111	A*02:01:01G	C*02:02:02G	B*55:01:01G	DRB1*04:04:01	DQB1*06:02:01G
262	0,00111	A*02:01:01G	C*03:03:01G	B*55:01:01G	DRB1*14:01:01G	DQB1*05:03:01G
263	0,00111	A*02:01:01G	C*01:02:01G	B*56:01:01G	DRB1*03:01:01G	DQB1*06:02:01G
264	0,00111	A*02:01:01G	C*06:02:01G	B*57:01:01G	DRB1*04:03:01	DQB1*03:02:01G
265	0,00111	A*02:01:01G	C*08:01:01G	B*57:01:01G	DRB1*08:02:01	DQB1*03:02:01G
266	0,00111	A*02:01:01G	C*07:01:01G	B*57:03:01G	DRB1*11:03:01	DQB1*03:02:01G
267	0,00111	A*02:01:01G	C*07:01:01G	B*57:03:01G	DRB1*13:02:01G	DQB1*06:04:01G

268	0,00111	A*02:01:01G	C*02:02:02G	B*58:01:01G	DRB1*07:01:01G	DQB1*03:01:01G
269	0,00111	A*02:01:01G	C*03:02:01G	B*58:01:01G	DRB1*04:05:01	DQB1*02:01:01G
270	0,00111	A*02:01:01G	C*07:01:01G	B*58:01:01G	DRB1*04:03:01	DQB1*03:05:01G
271	0,00111	A*02:01:01G	C*07:01:01G	B*58:01:01G	DRB1*11:03:01	DQB1*03:01:01G
272	0,00111	A*02:02:01G	C*06:02:01G	B*15:08:01	DRB1*08:07	DQB1*03:01:01G
273	0,00111	A*02:02:01G	C*07:02:01G	B*41:01:01	DRB1*11:04:01G	DQB1*03:02:01G
274	0,00111	A*02:02:01G	C*17:01:01G	B*41:01:01	DRB1*03:01:01G	DQB1*02:01:01G
275	0,00111	A*02:04	C*15:02:01G	B*51:01:01G	DRB1*15:01:01G	DQB1*03:01:01G
276	0,00111	A*02:05:01G	C*06:02:01G	B*15:20	DRB1*13:01:01G	DQB1*06:03:01G
277	0,00111	A*02:05:01G	C*04:01:01G	B*35:01:01G	DRB1*14:01:01G	DQB1*05:03:01G
278	0,00111	A*02:05:01G	C*06:02:01G	B*50:01:01G	DRB1*15:01:01G	DQB1*06:02:01G
279	0,00111	A*02:05:01G	C*03:03:01G	B*51:01:01G	DRB1*16:02:01G	DQB1*03:01:01G
280	0,00111	A*02:05:01G	C*07:01:01G	B*55:01:01G	DRB1*07:01:01G	DQB1*03:01:01G
281	0,00111	A*02:05:01G	C*07:01:01G	B*58:01:01G	DRB1*04:01:01G	DQB1*03:02:01G
282	0,00111	A*02:06:01G	C*04:01:01G	B*35:05:01	DRB1*14:01:01G	DQB1*03:01:01G
283	0,00111	A*02:06:01G	C*05:01:01G	B*44:02:01G	DRB1*12:01:01G	DQB1*03:01:01G
284	0,00111	A*02:06:01G	C*08:03:01G	B*48:01:01G	DRB1*04:03:01	DQB1*03:04:01
285	0,00111	A*02:06:01G	C*15:02:01G	B*51:01:01G	DRB1*14:01:01G	DQB1*05:03:01G
286	0,00111	A*02:11:01G	C*12:03:01G	B*39:01:01G	DRB1*13:01:01G	DQB1*06:03:01G
287	0,00111	A*02:11:01G	C*03:04:01G	B*40:04	DRB1*14:02:01	DQB1*03:01:01G
288	0,00111	A*02:11:01G	C*04:01:01G	B*48:02:01	DRB1*04:07:01G	DQB1*03:02:01G
289	0,00111	A*02:11:01G	C*04:01:01G	B*48:02:01	DRB1*16:02:01G	DQB1*03:01:01G
290	0,00111	A*02:13	C*15:02:01G	B*51:01:01G	DRB1*04:07:01G	DQB1*03:02:01G
291	0,00111	A*02:22:01G	C*07:02:01G	B*08:01:01G	DRB1*01:02:01	DQB1*04:02:01G
292	0,00111	A*03:01:01G	C*07:02:01G	B*07:02:01G	DRB1*04:02:01	DQB1*03:02:01G
293	0,00111	A*03:01:01G	C*07:02:01G	B*07:02:01G	DRB1*13:02:01G	DQB1*05:02:01G
294	0,00111	A*03:01:01G	C*07:02:01G	B*07:02:01G	DRB1*14:01:01G	DQB1*05:03:01G

295	0,00111	A*03:01:01G	C*07:02:01G	B*08:01:01G	DRB1*04:03:01	DQB1*03:02:01G
296	0,00111	A*03:01:01G	C*03:04:01G	B*14:01:01	DRB1*04:01:01G	DQB1*05:01:01G
297	0,00111	A*03:01:01G	C*08:02:01G	B*14:01:01	DRB1*04:05:01	DQB1*02:01:01G
298	0,00111	A*03:01:01G	C*15:05:01G	B*14:02:01G	DRB1*01:02:01	DQB1*05:01:01G
299	0,00111	A*03:01:01G	C*16:04:01	B*14:02:01G	DRB1*04:02:01	DQB1*05:01:01G
300	0,00111	A*03:01:01G	C*03:03:01G	B*15:01:01G	DRB1*11:03:01	DQB1*03:01:01G
301	0,00111	A*03:01:01G	C*07:02:01G	B*15:01:01G	DRB1*12:01:01G	DQB1*03:01:01G
302	0,00111	A*03:01:01G	C*07:04:01G	B*15:01:01G	DRB1*04:02:01	DQB1*06:03:01G
303	0,00111	A*03:01:01G	C*07:04:01G	B*15:18:01G	DRB1*13:01:01G	DQB1*06:03:01G
304	0,00111	A*03:01:01G	C*03:04:01G	B*18:01:01G	DRB1*11:01:01G	DQB1*03:01:01G
305	0,00111	A*03:01:01G	C*07:01:01G	B*18:01:01G	DRB1*11:04:01G	DQB1*03:01:01G
306	0,00111	A*03:01:01G	C*04:01:01G	B*35:01:01G	DRB1*11:01:01G	DQB1*03:01:01G
307	0,00111	A*03:01:01G	C*04:01:01G	B*35:01:01G	DRB1*13:01:01G	DQB1*06:03:01G
308	0,00111	A*03:01:01G	C*04:01:01G	B*35:01:01G	DRB1*13:05:01	DQB1*03:01:01G
309	0,00111	A*03:01:01G	C*08:02:01G	B*35:01:01G	DRB1*01:02:01	DQB1*05:01:01G
310	0,00111	A*03:01:01G	C*03:04:01G	B*35:03:01G	DRB1*15:01:01G	DQB1*05:02:01G
311	0,00111	A*03:01:01G	C*12:03:01G	B*35:03:01G	DRB1*14:01:01G	DQB1*05:03:01G
312	0,00111	A*03:01:01G	C*15:03	B*35:03:01G	DRB1*08:04:01	DQB1*05:03:01G
313	0,00111	A*03:01:01G	C*04:01:01G	B*35:04:01	DRB1*01:02:01	DQB1*05:01:01G
314	0,00111	A*03:01:01G	C*12:03:01G	B*38:01:01	DRB1*11:04:01G	DQB1*03:01:01G
315	0,00111	A*03:01:01G	C*07:01:01G	B*40:01:01G	DRB1*03:01:01G	DQB1*02:01:01G
316	0,00111	A*03:01:01G	C*08:02:01G	B*40:01:01G	DRB1*01:01:01G	DQB1*03:01:01G
317	0,00111	A*03:01:01G	C*12:03:01G	B*40:01:01G	DRB1*13:01:01G	DQB1*06:03:01G
318	0,00111	A*03:01:01G	C*02:02:02G	B*44:02:01G	DRB1*08:03:02	DQB1*03:01:01G
319	0,00111	A*03:01:01G	C*07:04:01G	B*44:02:01G	DRB1*13:02:01G	DQB1*06:04:01G
320	0,00111	A*03:01:01G	C*07:01:01G	B*44:03:01G	DRB1*07:01:01G	DQB1*03:03:02G
321	0,00111	A*03:01:01G	C*02:02:02G	B*44:05:01	DRB1*15:01:01G	DQB1*03:01:01G

322	0,00111	A*03:01:01G	C*16:01:01G	B*45:01:01G	DRB1*13:02:01G	DQB1*06:04:01G
323	0,00111	A*03:01:01G	C*07:01:01G	B*47:01:01G	DRB1*13:01:01G	DQB1*03:01:01G
324	0,00111	A*03:01:01G	C*12:03:01G	B*47:01:01G	DRB1*04:05:01	DQB1*03:02:01G
325	0,00111	A*03:01:01G	C*08:01:01G	B*48:01:01G	DRB1*09:01:02G	DQB1*03:01:01G
326	0,00111	A*03:01:01G	C*07:01:01G	B*49:01:01G	DRB1*03:01:01G	DQB1*02:01:01G
327	0,00111	A*03:01:01G	C*04:01:01G	B*51:01:01G	DRB1*01:01:01G	DQB1*06:03:01G
328	0,00111	A*03:01:01G	C*04:01:01G	B*51:01:01G	DRB1*04:07:01G	DQB1*04:02:01G
329	0,00111	A*03:01:01G	C*14:02:01G	B*51:01:01G	DRB1*07:01:01G	DQB1*02:01:01G
330	0,00111	A*03:01:01G	C*15:02:01G	B*51:01:01G	DRB1*14:01:01G	DQB1*05:03:01G
331	0,00111	A*03:01:01G	C*15:02:01G	B*52:01:02	DRB1*04:07:01G	DQB1*05:01:01G
332	0,00111	A*03:01:01G	C*03:03:01G	B*55:01:01G	DRB1*14:01:01G	DQB1*05:02:01G
333	0,00111	A*03:02:01G	C*08:02:01G	B*44:02:01G	DRB1*10:01:01G	DQB1*03:02:01G
334	0,00111	A*03:02:01G	C*04:01:01G	B*51:01:01G	DRB1*04:02:01	DQB1*03:02:01G
335	0,00111	A*03:02:01G	C*15:02:01G	B*51:01:01G	DRB1*11:01:01G	DQB1*03:01:01G
336	0,00111	A*03:08	C*07:02:01G	B*08:01:01G	DRB1*11:01:01G	DQB1*03:01:01G
337	0,00111	A*11:01:01G	C*06:02:01G	B*13:02:01G	DRB1*13:01:01G	DQB1*06:03:01G
338	0,00111	A*11:01:01G	C*08:02:01G	B*14:01:01	DRB1*01:03	DQB1*05:01:01G
339	0,00111	A*11:01:01G	C*08:02:01G	B*14:01:01	DRB1*07:01:01G	DQB1*02:01:01G
340	0,00111	A*11:01:01G	C*08:02:01G	B*14:02:01G	DRB1*01:02:01	DQB1*05:01:01G
341	0,00111	A*11:01:01G	C*02:02:02G	B*15:01:01G	DRB1*04:01:01G	DQB1*03:04:01
342	0,00111	A*11:01:01G	C*12:03:01G	B*18:01:01G	DRB1*15:01:01G	DQB1*06:02:01G
343	0,00111	A*11:01:01G	C*07:01:01G	B*18:03	DRB1*11:04:01G	DQB1*03:01:01G
344	0,00111	A*11:01:01G	C*02:02:02G	B*27:05:02G	DRB1*09:01:02G	DQB1*03:03:02G
345	0,00111	A*11:01:01G	C*02:02:02G	B*27:05:02G	DRB1*14:01:01G	DQB1*05:03:01G
346	0,00111	A*11:01:01G	C*04:01:01G	B*35:01:01G	DRB1*01:01:01G	DQB1*05:01:01G
347	0,00111	A*11:01:01G	C*04:01:01G	B*35:01:01G	DRB1*13:01:01G	DQB1*06:03:01G
348	0,00111	A*11:01:01G	C*04:01:01G	B*35:01:01G	DRB1*16:01:01	DQB1*03:02:01G

349	0,00111	A*11:01:01G	C*14:02:01G	B*35:01:01G	DRB1*08:01:01G	DQB1*03:01:01G
350	0,00111	A*11:01:01G	C*04:01:01G	B*35:03:01G	DRB1*04:01:01G	DQB1*03:01:01G
351	0,00111	A*11:01:01G	C*04:01:01G	B*38:01:01	DRB1*07:01:01G	DQB1*05:02:01G
352	0,00111	A*11:01:01G	C*06:02:01G	B*38:01:01	DRB1*13:01:01G	DQB1*06:03:01G
353	0,00111	A*11:01:01G	C*12:03:01G	B*38:01:01	DRB1*13:01:01G	DQB1*06:03:01G
354	0,00111	A*11:01:01G	C*07:02:01G	B*39:05:01	DRB1*04:04:01	DQB1*03:02:01G
355	0,00111	A*11:01:01G	C*07:02:01G	B*39:09:01	DRB1*13:01:01G	DQB1*06:03:01G
356	0,00111	A*11:01:01G	C*03:04:01G	B*40:01:01G	DRB1*01:01:01G	DQB1*03:02:01G
357	0,00111	A*11:01:01G	C*04:01:01G	B*41:01:01	DRB1*11:02:01	DQB1*05:03:01G
358	0,00111	A*11:01:01G	C*05:01:01G	B*44:02:01G	DRB1*07:01:01G	DQB1*05:03:01G
359	0,00111	A*11:01:01G	C*02:02:02G	B*44:03:01G	DRB1*09:01:02G	DQB1*03:03:02G
360	0,00111	A*11:01:01G	C*06:02:01G	B*44:03:01G	DRB1*03:01:01G	DQB1*02:01:01G
361	0,00111	A*11:01:01G	C*16:01:01G	B*44:03:01G	DRB1*11:01:01G	DQB1*03:01:01G
362	0,00111	A*11:01:01G	C*04:01:01G	B*44:03:02G	DRB1*04:01:01G	DQB1*05:03:01G
363	0,00111	A*11:01:01G	C*07:01:01G	B*49:01:01G	DRB1*14:02:01	DQB1*03:01:01G
364	0,00111	A*11:01:01G	C*07:01:01G	B*49:01:01G	DRB1*15:01:01G	DQB1*02:01:01G
365	0,00111	A*11:01:01G	C*16:01:01G	B*50:01:01G	DRB1*07:01:01G	DQB1*02:01:01G
366	0,00111	A*11:01:01G	C*06:02:01G	B*50:02	DRB1*04:06:01G	DQB1*04:02:01G
367	0,00111	A*11:01:01G	C*03:03:01G	B*51:01:01G	DRB1*15:01:01G	DQB1*03:02:01G
368	0,00111	A*11:01:01G	C*12:02:01G	B*51:01:01G	DRB1*15:02:01G	DQB1*06:01:01G
369	0,00111	A*11:01:01G	C*15:02:01G	B*51:01:01G	DRB1*04:02:01	DQB1*03:02:01G
370	0,00111	A*11:01:01G	C*15:02:01G	B*51:01:01G	DRB1*08:02:01	DQB1*04:02:01G
371	0,00111	A*11:01:01G	C*15:02:01G	B*51:01:01G	DRB1*11:02:01	DQB1*03:01:01G
372	0,00111	A*11:01:01G	C*15:02:01G	B*52:01:02	DRB1*14:02:01	DQB1*06:02:01G
373	0,00111	A*11:01:01G	C*03:03:01G	B*55:01:01G	DRB1*04:04:01	DQB1*05:01:01G
374	0,00111	A*11:01:01G	C*12:03:01G	B*58:01:01G	DRB1*08:02:01	DQB1*04:02:01G
375	0,00111	A*23:01:01G	C*06:02:01G	B*07:02:01G	DRB1*04:08:01	DQB1*05:01:01G

376	0,00111	A*23:01:01G	C*07:02:01G	B*07:02:01G	DRB1*04:05:01	DQB1*03:02:01G
377	0,00111	A*23:01:01G	C*02:10:01G	B*15:03:01G	DRB1*11:01:02	DQB1*03:01:01G
378	0,00111	A*23:01:01G	C*07:01:01G	B*15:27:02	DRB1*04:07:01G	DQB1*03:01:01G
379	0,00111	A*23:01:01G	C*02:02:02G	B*41:01:01	DRB1*13:02:01G	DQB1*06:04:01G
380	0,00111	A*23:01:01G	C*05:01:01G	B*44:02:01G	DRB1*13:02:01G	DQB1*06:09:01G
381	0,00111	A*23:01:01G	C*04:01:01G	B*44:03:01G	DRB1*03:01:01G	DQB1*02:01:01G
382	0,00111	A*23:01:01G	C*04:01:01G	B*44:03:01G	DRB1*13:01:01G	DQB1*06:03:01G
383	0,00111	A*23:01:01G	C*04:01:01G	B*44:03:01G	DRB1*15:01:01G	DQB1*06:02:01G
384	0,00111	A*23:01:01G	C*12:03:01G	B*44:03:01G	DRB1*16:01:01	DQB1*02:01:01G
385	0,00111	A*23:01:01G	C*15:05:01G	B*45:01:01G	DRB1*01:02:01	DQB1*03:01:01G
386	0,00111	A*23:01:01G	C*04:01:01G	B*53:01:01G	DRB1*08:02:01	DQB1*03:01:01G
387	0,00111	A*23:01:01G	C*03:03:01G	B*55:01:01G	DRB1*04:03:01	DQB1*03:02:01G
388	0,00111	A*23:01:01G	C*04:01:01G	B*56:01:01G	DRB1*15:03:01G	DQB1*05:01:01G
389	0,00111	A*23:01:01G	C*07:01:01G	B*57:01:01G	DRB1*07:01:01G	DQB1*06:02:01G
390	0,00111	A*23:01:01G	C*07:01:01G	B*57:01:01G	DRB1*11:04:01G	DQB1*03:01:01G
391	0,00111	A*23:01:01G	C*07:01:01G	B*58:01:01G	DRB1*04:02:01	DQB1*03:01:01G
392	0,00111	A*23:01:01G	C*07:01:01G	B*58:01:01G	DRB1*13:01:01G	DQB1*03:03:02G
393	0,00111	A*24:02:01G	C*07:02:01G	B*07:02:01G	DRB1*07:01:01G	DQB1*06:02:01G
394	0,00111	A*24:02:01G	C*15:05:01G	B*07:05:01G	DRB1*16:01:01	DQB1*02:01:01G
395	0,00111	A*24:02:01G	C*02:02:02G	B*14:02:01G	DRB1*01:02:01	DQB1*03:01:01G
396	0,00111	A*24:02:01G	C*03:03:01G	B*15:01:01G	DRB1*13:01:01G	DQB1*03:03:02G
397	0,00111	A*24:02:01G	C*05:01:01G	B*15:01:01G	DRB1*13:01:01G	DQB1*05:01:01G
398	0,00111	A*24:02:01G	C*07:01:01G	B*15:17:01G	DRB1*15:01:01G	DQB1*06:02:01G
399	0,00111	A*24:02:01G	C*04:01:01G	B*18:01:01G	DRB1*14:01:01G	DQB1*05:01:01G
400	0,00111	A*24:02:01G	C*07:01:01G	B*18:01:01G	DRB1*11:01:01G	DQB1*03:02:01G
401	0,00111	A*24:02:01G	C*07:01:01G	B*18:01:01G	DRB1*13:01:01G	DQB1*03:02:01G
402	0,00111	A*24:02:01G	C*07:01:01G	B*18:01:01G	DRB1*13:05:01	DQB1*03:01:01G

403	0,00111	A*24:02:01G	C*07:01:01G	B*18:01:01G	DRB1*16:01:01	DQB1*05:02:01G
404	0,00111	A*24:02:01G	C*02:02:02G	B*27:05:02G	DRB1*08:07	DQB1*04:02:01G
405	0,00111	A*24:02:01G	C*02:02:02G	B*27:05:02G	DRB1*11:01:01G	DQB1*03:01:01G
406	0,00111	A*24:02:01G	C*15:02:01G	B*27:05:02G	DRB1*04:01:01G	DQB1*03:01:01G
407	0,00111	A*24:02:01G	C*04:01:01G	B*35:02:01G	DRB1*07:01:01G	DQB1*02:01:01G
408	0,00111	A*24:02:01G	C*14:02:01G	B*35:05:01	DRB1*14:02:01	DQB1*03:01:01G
409	0,00111	A*24:02:01G	C*04:01:01G	B*35:08:01	DRB1*13:02:01G	DQB1*06:04:01G
410	0,00111	A*24:02:01G	C*04:01:01G	B*35:09:01	DRB1*08:02:01	DQB1*05:01:01G
411	0,00111	A*24:02:01G	C*12:03:01G	B*38:01:01	DRB1*04:02:01	DQB1*04:02:01G
412	0,00111	A*24:02:01G	C*12:03:01G	B*38:01:01	DRB1*07:01:01G	DQB1*02:01:01G
413	0,00111	A*24:02:01G	C*12:03:01G	B*38:01:01	DRB1*13:03:01	DQB1*03:01:01G
414	0,00111	A*24:02:01G	C*04:01:01G	B*39:01:01G	DRB1*14:02:01	DQB1*05:01:01G
415	0,00111	A*24:02:01G	C*12:03:01G	B*39:01:01G	DRB1*08:02:01	DQB1*06:02:01G
416	0,00111	A*24:02:01G	C*07:02:01G	B*39:03	DRB1*14:06:01	DQB1*03:01:01G
417	0,00111	A*24:02:01G	C*07:02:01G	B*39:05:01	DRB1*13:02:01G	DQB1*03:02:01G
418	0,00111	A*24:02:01G	C*07:02:01G	B*39:09:01	DRB1*04:02:01	DQB1*03:02:01G
419	0,00111	A*24:02:01G	C*16:01:01G	B*39:09:01	DRB1*08:02:01	DQB1*05:01:01G
420	0,00111	A*24:02:01G	C*03:04:01G	B*40:01:01G	DRB1*14:01:01G	DQB1*05:03:01G
421	0,00111	A*24:02:01G	C*04:01:01G	B*40:01:01G	DRB1*04:05:01	DQB1*03:02:01G
422	0,00111	A*24:02:01G	C*02:02:02G	B*40:02:01G	DRB1*04:05:01	DQB1*03:02:01G
423	0,00111	A*24:02:01G	C*03:05	B*40:02:01G	DRB1*04:07:01G	DQB1*03:02:01G
424	0,00111	A*24:02:01G	C*08:03:01G	B*40:02:01G	DRB1*09:01:02G	DQB1*03:03:02G
425	0,00111	A*24:02:01G	C*15:02:01G	B*40:02:01G	DRB1*01:01:01G	DQB1*05:01:01G
426	0,00111	A*24:02:01G	C*03:04:01G	B*40:04	DRB1*04:07:01G	DQB1*03:02:01G
427	0,00111	A*24:02:01G	C*03:04:01G	B*40:04	DRB1*04:11:01	DQB1*03:02:01G
428	0,00111	A*24:02:01G	C*05:01:01G	B*44:02:01G	DRB1*04:03:01	DQB1*03:04:01
429	0,00111	A*24:02:01G	C*01:02:01G	B*44:03:01G	DRB1*01:01:01G	DQB1*06:02:01G

430	0,00111	A*24:02:01G	C*01:02:01G	B*44:03:01G	DRB1*07:01:01G	DQB1*03:02:01G
431	0,00111	A*24:02:01G	C*02:02:02G	B*44:03:01G	DRB1*13:03:01	DQB1*03:01:01G
432	0,00111	A*24:02:01G	C*07:02:01G	B*45:01:01G	DRB1*10:01:01G	DQB1*03:02:01G
433	0,00111	A*24:02:01G	C*16:01:01G	B*45:01:01G	DRB1*14:01:01G	DQB1*05:03:01G
434	0,00111	A*24:02:01G	C*06:30	B*47:01:01G	DRB1*16:01:01	DQB1*05:02:01G
435	0,00111	A*24:02:01G	C*12:03:01G	B*48:01:01G	DRB1*01:01:01G	DQB1*05:01:01G
436	0,00111	A*24:02:01G	C*02:02:02G	B*51:01:01G	DRB1*01:03	DQB1*05:01:01G
437	0,00111	A*24:02:01G	C*03:03:01G	B*51:01:01G	DRB1*01:02:01	DQB1*05:01:01G
438	0,00111	A*24:02:01G	C*07:02:01G	B*51:01:01G	DRB1*13:02:01G	DQB1*04:02:01G
439	0,00111	A*24:02:01G	C*16:02:01G	B*51:08:01	DRB1*16:01:01	DQB1*05:02:01G
440	0,00111	A*24:02:01G	C*04:01:01G	B*53:01:01G	DRB1*08:07	DQB1*06:04:01G
441	0,00111	A*24:02:01G	C*06:02:01G	B*53:01:01G	DRB1*13:03:01	DQB1*02:01:01G
442	0,00111	A*24:02:01G	C*04:01:01G	B*57:01:01G	DRB1*04:02:01	DQB1*06:03:01G
443	0,00111	A*24:02:01G	C*07:02:01G	B*58:01:01G	DRB1*03:01:01G	DQB1*02:01:01G
444	0,00111	A*24:02:01G	C*12:03:01G	B*67:01:02G	DRB1*01:01:01G	DQB1*05:01:01G
445	0,00111	A*24:02:01G	C*02:14:01G	B*81:01:01G	DRB1*12:01:01G	DQB1*05:01:01G
446	0,00111	A*24:02:02	C*08:02:01G	B*49:01:01G	DRB1*10:01:01G	DQB1*05:01:01G
447	0,00111	A*24:03:01G	C*07:02:01G	B*07:02:01G	DRB1*11:04:01G	DQB1*03:01:01G
448	0,00111	A*24:03:01G	C*07:02:01G	B*07:02:01G	DRB1*15:01:01G	DQB1*05:03:01G
449	0,00111	A*24:03:01G	C*01:02:01G	B*15:20	DRB1*04:11:01	DQB1*03:02:01G
450	0,00111	A*24:03:01G	C*04:01:01G	B*35:01:01G	DRB1*04:10:01G	DQB1*03:02:01G
451	0,00111	A*24:03:01G	C*04:01:01G	B*37:01:01G	DRB1*01:01:01G	DQB1*05:01:01G
452	0,00111	A*24:03:01G	C*15:02:01G	B*39:01:01G	DRB1*08:07	DQB1*06:04:01G
453	0,00111	A*24:03:01G	C*05:01:01G	B*40:04	DRB1*04:07:03	DQB1*03:02:01G
454	0,00111	A*24:03:01G	C*14:02:01G	B*51:01:01G	DRB1*11:01:01G	DQB1*03:01:01G
455	0,00111	A*24:03:01G	C*15:02:01G	B*51:13:01	DRB1*08:02:01	DQB1*04:02:01G
456	0,00111	A*24:175	C*07:02:01G	B*35:04:01	DRB1*01:01:01G	DQB1*03:01:01G

457	0,00111	A*25:01:01G	C*07:02:01G	B*07:02:01G	DRB1*01:01:01G	DQB1*05:01:01G
458	0,00111	A*25:01:01G	C*03:03:01G	B*15:01:01G	DRB1*01:01:01G	DQB1*05:01:01G
459	0,00111	A*25:01:01G	C*04:01:01G	B*18:01:01G	DRB1*07:01:01G	DQB1*02:01:01G
460	0,00111	A*25:01:01G	C*05:01:01G	B*18:01:01G	DRB1*14:01:01G	DQB1*05:03:01G
461	0,00111	A*25:01:01G	C*12:03:01G	B*18:01:01G	DRB1*01:03	DQB1*05:01:01G
462	0,00111	A*25:01:01G	C*12:03:01G	B*35:03:01G	DRB1*01:03	DQB1*05:03:01G
463	0,00111	A*25:01:01G	C*07:01:01G	B*39:05:01	DRB1*08:02:01	DQB1*04:02:01G
464	0,00111	A*25:01:01G	C*15:02:01G	B*40:02:01G	DRB1*14:02:01	DQB1*06:02:01G
465	0,00111	A*25:01:01G	C*07:02:01G	B*51:01:01G	DRB1*14:02:01	DQB1*03:01:01G
466	0,00111	A*25:01:01G	C*03:03:01G	B*55:01:01G	DRB1*14:01:01G	DQB1*05:03:01G
467	0,00111	A*26:01:01G	C*07:02:01G	B*07:02:01G	DRB1*11:03:01	DQB1*03:01:01G
468	0,00111	A*26:01:01G	C*08:02:01G	B*14:01:01	DRB1*01:03	DQB1*05:01:01G
469	0,00111	A*26:01:01G	C*08:02:01G	B*14:01:01	DRB1*15:02:01G	DQB1*06:01:01G
470	0,00111	A*26:01:01G	C*03:03:01G	B*15:04:01G	DRB1*16:02:01G	DQB1*03:01:01G
471	0,00111	A*26:01:01G	C*02:02:02G	B*15:18:01G	DRB1*13:01:01G	DQB1*02:01:01G
472	0,00111	A*26:01:01G	C*07:01:01G	B*18:01:01G	DRB1*13:05:01	DQB1*03:01:01G
473	0,00111	A*26:01:01G	C*07:01:01G	B*18:01:01G	DRB1*15:01:01G	DQB1*02:01:01G
474	0,00111	A*26:01:01G	C*12:03:01G	B*18:01:01G	DRB1*11:04:01G	DQB1*03:01:01G
475	0,00111	A*26:01:01G	C*02:02:02G	B*27:02:01	DRB1*15:01:01G	DQB1*06:02:01G
476	0,00111	A*26:01:01G	C*07:01:01G	B*27:07:01G	DRB1*13:01:01G	DQB1*06:03:01G
477	0,00111	A*26:01:01G	C*04:01:01G	B*35:01:01G	DRB1*04:07:01G	DQB1*03:01:01G
478	0,00111	A*26:01:01G	C*12:03:01G	B*35:08:01	DRB1*13:03:01	DQB1*03:01:01G
479	0,00111	A*26:01:01G	C*04:01:01G	B*35:20:01	DRB1*08:02:01	DQB1*03:02:01G
480	0,00111	A*26:01:01G	C*05:01:01G	B*38:01:01	DRB1*04:02:01	DQB1*04:02:01G
481	0,00111	A*26:01:01G	C*12:03:01G	B*44:02:01G	DRB1*08:01:01G	DQB1*03:02:01G
482	0,00111	A*26:01:01G	C*12:03:01G	B*44:03:01G	DRB1*11:04:01G	DQB1*03:01:01G
483	0,00111	A*26:01:01G	C*16:01:01G	B*44:03:01G	DRB1*03:01:01G	DQB1*03:02:01G

484	0,00111	A*26:01:01G	C*07:02:01G	B*51:01:01G	DRB1*13:01:01G	DQB1*03:01:01G
485	0,00111	A*26:01:01G	C*15:02:01G	B*51:01:01G	DRB1*11:01:01G	DQB1*03:01:01G
486	0,00111	A*26:01:01G	C*14:02:01G	B*51:07:01	DRB1*15:01:01G	DQB1*05:02:01G
487	0,00111	A*26:01:01G	C*04:01:01G	B*53:01:01G	DRB1*03:02:01	DQB1*04:02:01G
488	0,00111	A*26:01:01G	C*08:02:01G	B*55:01:01G	DRB1*11:01:02	DQB1*06:02:01G
489	0,00111	A*29:01:01G	C*15:05:01G	B*07:05:01G	DRB1*09:01:02G	DQB1*03:03:02G
490	0,00111	A*29:01:01G	C*07:01:01G	B*44:02:01G	DRB1*11:04:01G	DQB1*03:01:01G
491	0,00111	A*29:02:01G	C*07:01:01G	B*08:01:01G	DRB1*15:01:01G	DQB1*05:01:01G
492	0,00111	A*29:02:01G	C*16:01:01G	B*14:02:01G	DRB1*01:02:01	DQB1*03:01:01G
493	0,00111	A*29:02:01G	C*14:02:01G	B*15:04:01G	DRB1*13:01:01G	DQB1*06:03:01G
494	0,00111	A*29:02:01G	C*06:02:01G	B*15:15	DRB1*04:01:01G	DQB1*03:01:01G
495	0,00111	A*29:02:01G	C*16:01:01G	B*18:01:01G	DRB1*11:04:01G	DQB1*03:01:01G
496	0,00111	A*29:02:01G	C*16:01:01G	B*44:03:01G	DRB1*11:01:01G	DQB1*03:01:01G
497	0,00111	A*29:02:01G	C*16:01:01G	B*44:03:01G	DRB1*12:01:01G	DQB1*03:02:01G
498	0,00111	A*29:02:01G	C*16:01:01G	B*44:03:01G	DRB1*15:01:01G	DQB1*06:02:01G
499	0,00111	A*29:02:01G	C*08:02:01G	B*44:04	DRB1*11:01:01G	DQB1*05:01:01G
500	0,00111	A*29:02:01G	C*16:01:01G	B*44:04	DRB1*11:01:01G	DQB1*03:01:01G
501	0,00111	A*29:02:01G	C*04:01:01G	B*51:01:01G	DRB1*11:04:01G	DQB1*03:01:01G
502	0,00111	A*29:02:01G	C*07:01:01G	B*57:03:01G	DRB1*11:01:01G	DQB1*03:01:01G
503	0,00111	A*30:01:01G	C*07:02:01G	B*07:02:01G	DRB1*07:01:01G	DQB1*03:03:02G
504	0,00111	A*30:01:01G	C*06:02:01G	B*13:02:01G	DRB1*03:01:01G	DQB1*02:01:01G
505	0,00111	A*30:01:01G	C*06:02:01G	B*13:02:01G	DRB1*14:01:01G	DQB1*05:03:01G
506	0,00111	A*30:01:01G	C*07:02:01G	B*39:01:01G	DRB1*14:01:01G	DQB1*05:03:01G
507	0,00111	A*30:01:01G	C*04:01:01G	B*44:03:01G	DRB1*15:01:01G	DQB1*06:02:01G
508	0,00111	A*30:01:01G	C*17:01:01G	B*44:03:01G	DRB1*01:01:01G	DQB1*05:01:01G
509	0,00111	A*30:01:01G	C*02:02:02G	B*44:05:01	DRB1*01:01:01G	DQB1*05:01:01G
510	0,00111	A*30:01:01G	C*07:01:01G	B*51:01:01G	DRB1*14:01:01G	DQB1*02:01:01G

511	0,00111	A*30:01:01G	C*12:02:01G	B*52:01:01G	DRB1*16:01:01	DQB1*05:02:01G
512	0,00111	A*30:01:01G	C*06:02:01G	B*58:02:01	DRB1*15:03:01G	DQB1*06:02:01G
513	0,00111	A*30:02:01G	C*03:03:01G	B*15:01:01G	DRB1*08:02:01	DQB1*05:02:01G
514	0,00111	A*30:02:01G	C*02:10:01G	B*15:03:01G	DRB1*11:04:01G	DQB1*06:11:01
515	0,00111	A*30:02:01G	C*03:04:01G	B*27:02:01	DRB1*13:02:01G	DQB1*06:04:01G
516	0,00111	A*30:02:01G	C*04:01:01G	B*35:01:01G	DRB1*08:01:01G	DQB1*04:02:01G
517	0,00111	A*30:02:01G	C*04:01:01G	B*35:01:01G	DRB1*15:02:01G	DQB1*06:01:01G
518	0,00111	A*30:02:01G	C*12:03:01G	B*39:01:01G	DRB1*01:02:01	DQB1*05:01:01G
519	0,00111	A*30:02:01G	C*07:01:01G	B*39:05:01	DRB1*14:02:01	DQB1*03:01:01G
520	0,00111	A*30:02:01G	C*07:01:01G	B*40:02:01G	DRB1*13:03:01	DQB1*03:01:01G
521	0,00111	A*30:02:01G	C*18:01:01G	B*57:03:01G	DRB1*13:01:01G	DQB1*05:01:01G
522	0,00111	A*30:02:01G	C*07:01:01G	B*58:01:01G	DRB1*13:02:01G	DQB1*06:04:01G
523	0,00111	A*30:04:01G	C*06:02:01G	B*58:02:01	DRB1*13:03:01	DQB1*03:01:01G
524	0,00111	A*31:01:02G	C*15:02:01G	B*07:02:01G	DRB1*01:03	DQB1*05:01:01G
525	0,00111	A*31:01:02G	C*15:05:01G	B*07:05:01G	DRB1*04:03:01	DQB1*03:02:01G
526	0,00111	A*31:01:02G	C*06:02:01G	B*13:02:01G	DRB1*13:01:01G	DQB1*06:03:01G
527	0,00111	A*31:01:02G	C*08:02:01G	B*14:02:01G	DRB1*01:02:01	DQB1*05:01:01G
528	0,00111	A*31:01:02G	C*08:02:01G	B*14:02:01G	DRB1*15:03:01G	DQB1*06:02:01G
529	0,00111	A*31:01:02G	C*03:03:01G	B*15:01:01G	DRB1*04:08:01	DQB1*03:01:01G
530	0,00111	A*31:01:02G	C*07:01:01G	B*15:01:01G	DRB1*07:01:01G	DQB1*03:02:01G
531	0,00111	A*31:01:02G	C*03:03:01G	B*15:04:01G	DRB1*09:01:02G	DQB1*03:03:02G
532	0,00111	A*31:01:02G	C*07:02:01G	B*15:10:01	DRB1*04:11:01	DQB1*03:02:01G
533	0,00111	A*31:01:02G	C*03:03:01G	B*15:70	DRB1*04:04:01	DQB1*03:01:01G
534	0,00111	A*31:01:02G	C*07:02:01G	B*18:01:01G	DRB1*13:01:01G	DQB1*06:03:01G
535	0,00111	A*31:01:02G	C*15:02:01G	B*35:01:01G	DRB1*08:02:01	DQB1*05:01:01G
536	0,00111	A*31:01:02G	C*08:03:01G	B*35:04:01	DRB1*14:02:01	DQB1*03:01:01G
537	0,00111	A*31:01:02G	C*12:02:01G	B*35:09:01	DRB1*04:07:01G	DQB1*03:02:01G

538	0,00111	A*31:01:02G	C*07:02:01G	B*39:02:02	DRB1*16:02:01G	DQB1*03:01:01G
539	0,00111	A*31:01:02G	C*07:02:01G	B*39:03	DRB1*09:01:02G	DQB1*03:01:01G
540	0,00111	A*31:01:02G	C*07:02:01G	B*39:05:01	DRB1*04:07:03	DQB1*03:02:01G
541	0,00111	A*31:01:02G	C*07:02:01G	B*39:05:01	DRB1*08:02:01	DQB1*03:02:01G
542	0,00111	A*31:01:02G	C*07:02:01G	B*39:05:01	DRB1*14:02:01	DQB1*03:01:01G
543	0,00111	A*31:01:02G	C*17:01:01G	B*39:05:01	DRB1*04:03:01	DQB1*03:01:01G
544	0,00111	A*31:01:02G	C*03:04:01G	B*39:13:01	DRB1*16:02:01G	DQB1*04:02:01G
545	0,00111	A*31:01:02G	C*02:02:02G	B*40:02:01G	DRB1*16:01:01	DQB1*05:02:01G
546	0,00111	A*31:01:02G	C*03:03:01G	B*40:02:01G	DRB1*14:02:01	DQB1*03:01:01G
547	0,00111	A*31:01:02G	C*05:01:01G	B*44:02:01G	DRB1*01:01:01G	DQB1*05:01:01G
548	0,00111	A*31:01:02G	C*03:04:01G	B*45:01:01G	DRB1*04:04:01	DQB1*03:02:01G
549	0,00111	A*31:01:02G	C*08:03:01G	B*48:01:01G	DRB1*09:01:02G	DQB1*06:03:01G
550	0,00111	A*31:01:02G	C*01:02:01G	B*51:01:01G	DRB1*01:01:01G	DQB1*05:01:01G
551	0,00111	A*31:01:02G	C*04:01:01G	B*51:01:01G	DRB1*04:02:01	DQB1*02:01:01G
552	0,00111	A*31:01:02G	C*07:02:01G	B*51:01:01G	DRB1*10:01:01G	DQB1*03:01:01G
553	0,00111	A*31:01:02G	C*15:08	B*51:01:01G	DRB1*04:11:01	DQB1*03:02:01G
554	0,00111	A*31:01:02G	C*03:03:01G	B*55:01:01G	DRB1*16:01:01	DQB1*05:02:01G
555	0,00111	A*31:01:02G	C*07:02:01G	B*58:02:01	DRB1*11:02:01	DQB1*04:02:01G
556	0,00111	A*31:02	C*04:01:01G	B*39:05:01	DRB1*14:02:01	DQB1*03:01:01G
557	0,00111	A*32:01:01G	C*07:04:01G	B*07:02:01G	DRB1*13:01:01G	DQB1*06:03:01G
558	0,00111	A*32:01:01G	C*07:01:01G	B*08:01:01G	DRB1*07:01:01G	DQB1*05:02:01G
559	0,00111	A*32:01:01G	C*08:02:01G	B*14:01:01	DRB1*04:05:01	DQB1*02:01:01G
560	0,00111	A*32:01:01G	C*08:02:01G	B*14:01:01	DRB1*11:01:01G	DQB1*06:02:01G
561	0,00111	A*32:01:01G	C*08:02:01G	B*14:01:01	DRB1*15:01:01G	DQB1*04:02:01G
562	0,00111	A*32:01:01G	C*02:02:02G	B*14:02:01G	DRB1*01:02:01	DQB1*05:01:01G
563	0,00111	A*32:01:01G	C*08:02:01G	B*14:02:01G	DRB1*11:01:01G	DQB1*03:01:01G
564	0,00111	A*32:01:01G	C*03:03:01G	B*15:01:01G	DRB1*01:03	DQB1*06:03:01G

565	0,00111	A*32:01:01G	C*03:04:02	B*15:10:01	DRB1*13:02:01G	DQB1*05:01:01G
566	0,00111	A*32:01:01G	C*07:02:01G	B*18:01:01G	DRB1*04:03:01	DQB1*03:02:01G
567	0,00111	A*32:01:01G	C*03:03:01G	B*35:01:01G	DRB1*08:01:01G	DQB1*03:01:01G
568	0,00111	A*32:01:01G	C*04:01:01G	B*35:01:01G	DRB1*04:03:01	DQB1*03:02:01G
569	0,00111	A*32:01:01G	C*06:02:01G	B*35:01:01G	DRB1*11:01:01G	DQB1*03:01:01G
570	0,00111	A*32:01:01G	C*07:01:01G	B*35:01:01G	DRB1*14:01:01G	DQB1*03:02:01G
571	0,00111	A*32:01:01G	C*04:01:01G	B*35:03:01G	DRB1*11:01:01G	DQB1*03:01:01G
572	0,00111	A*32:01:01G	C*06:02:01G	B*35:03:01G	DRB1*13:01:01G	DQB1*03:02:01G
573	0,00111	A*32:01:01G	C*07:02:01G	B*39:06:02	DRB1*14:01:01G	DQB1*05:03:01G
574	0,00111	A*32:01:01G	C*03:04:01G	B*40:01:01G	DRB1*13:02:01G	DQB1*06:04:01G
575	0,00111	A*32:01:01G	C*03:04:01G	B*40:01:01G	DRB1*13:03:01	DQB1*06:03:01G
576	0,00111	A*32:01:01G	C*02:02:02G	B*40:02:01G	DRB1*14:01:01G	DQB1*05:03:01G
577	0,00111	A*32:01:01G	C*07:04:01G	B*40:02:01G	DRB1*07:01:01G	DQB1*06:03:01G
578	0,00111	A*32:01:01G	C*07:01:01G	B*41:01:01	DRB1*11:01:02	DQB1*03:01:01G
579	0,00111	A*32:01:01G	C*17:01:01G	B*41:01:01	DRB1*16:01:01	DQB1*05:02:01G
580	0,00111	A*32:01:01G	C*05:01:01G	B*44:02:01G	DRB1*16:01:01	DQB1*05:02:01G
581	0,00111	A*32:01:01G	C*07:01:01G	B*44:03:01G	DRB1*11:03:01	DQB1*03:01:01G
582	0,00111	A*32:01:01G	C*14:02:01G	B*51:01:01G	DRB1*13:05:01	DQB1*03:01:01G
583	0,00111	A*32:01:01G	C*16:04:01	B*51:01:01G	DRB1*11:04:01G	DQB1*03:01:01G
584	0,00111	A*32:01:01G	C*15:02:01G	B*51:08:01	DRB1*13:03:01	DQB1*03:02:01G
585	0,00111	A*32:01:01G	C*04:01:01G	B*52:01:01G	DRB1*15:02:01G	DQB1*06:01:01G
586	0,00111	A*32:01:01G	C*15:02:01G	B*52:01:01G	DRB1*04:08:01	DQB1*03:01:01G
587	0,00111	A*33:01:01G	C*07:02:01G	B*07:02:01G	DRB1*15:01:01G	DQB1*06:03:01G
588	0,00111	A*33:01:01G	C*12:03:01G	B*18:01:01G	DRB1*07:01:01G	DQB1*02:01:01G
589	0,00111	A*33:01:01G	C*02:02:02G	B*38:01:01	DRB1*15:01:01G	DQB1*06:02:01G
590	0,00111	A*33:01:01G	C*14:02:01G	B*44:03:01G	DRB1*04:01:01G	DQB1*03:02:01G
591	0,00111	A*33:01:01G	C*15:02:01G	B*51:13:01	DRB1*08:02:01	DQB1*04:02:01G

592	0,00111	A*33:01:01G	C*06:02:01G	B*57:01:01G	DRB1*07:01:01G	DQB1*03:03:02G
593	0,00111	A*33:01:01G	C*16:01:01G	B*78:01:01	DRB1*11:03:01	DQB1*05:01:01G
594	0,00111	A*33:03:01G	C*04:01:01G	B*35:01:01G	DRB1*04:02:01	DQB1*04:02:01G
595	0,00111	A*33:03:01G	C*04:01:01G	B*35:08:01	DRB1*04:07:01G	DQB1*05:01:01G
596	0,00111	A*33:03:01G	C*15:02:01G	B*51:01:01G	DRB1*04:07:01G	DQB1*03:01:01G
597	0,00111	A*33:03:01G	C*07:01:01G	B*58:01:01G	DRB1*13:02:01G	DQB1*06:09:01G
598	0,00111	A*33:05	C*08:02:01G	B*14:02:01G	DRB1*07:01:01G	DQB1*03:03:02G
599	0,00111	A*34:02:01	C*06:02:01G	B*35:01:01G	DRB1*04:01:01G	DQB1*03:02:01G
600	0,00111	A*66:01:01G	C*03:04:01G	B*15:01:01G	DRB1*14:02:01	DQB1*03:01:01G
601	0,00111	A*66:01:01G	C*03:04:01G	B*40:02:01G	DRB1*16:02:01G	DQB1*03:01:01G
602	0,00111	A*66:01:01G	C*08:01:01G	B*44:03:01G	DRB1*14:02:01	DQB1*03:01:01G
603	0,00111	A*66:01:01G	C*01:02:01G	B*49:18:02	DRB1*04:02:01	DQB1*02:01:01G
604	0,00111	A*66:01:01G	C*07:04:01G	B*58:02:01	DRB1*13:01:01G	DQB1*03:03:02G
605	0,00111	A*68:01:01G	C*03:03:01G	B*15:01:01G	DRB1*13:01:01G	DQB1*06:03:01G
606	0,00111	A*68:01:01G	C*12:03:01G	B*18:01:01G	DRB1*16:01:01	DQB1*05:02:01G
607	0,00111	A*68:01:01G	C*04:01:01G	B*35:01:01G	DRB1*01:01:01G	DQB1*05:01:01G
608	0,00111	A*68:01:01G	C*04:01:01G	B*35:01:01G	DRB1*16:01:01	DQB1*05:02:01G
609	0,00111	A*68:01:01G	C*04:01:01G	B*35:03:01G	DRB1*15:01:01G	DQB1*06:02:01G
610	0,00111	A*68:01:01G	C*03:04:01G	B*40:01:01G	DRB1*01:02:01	DQB1*05:01:01G
611	0,00111	A*68:01:01G	C*05:01:01G	B*44:02:01G	DRB1*11:04:01G	DQB1*03:01:01G
612	0,00111	A*68:01:02G	C*02:02:02G	B*07:02:01G	DRB1*15:01:01G	DQB1*04:02:01G
613	0,00111	A*68:01:02G	C*06:02:01G	B*13:02:01G	DRB1*13:05:01	DQB1*04:02:01G
614	0,00111	A*68:01:02G	C*07:01:01G	B*14:02:01G	DRB1*01:02:01	DQB1*05:01:01G
615	0,00111	A*68:01:02G	C*03:03:01G	B*15:04:01G	DRB1*14:02:01	DQB1*03:01:01G
616	0,00111	A*68:01:02G	C*15:02:01G	B*15:08:01	DRB1*09:01:02G	DQB1*03:03:02G
617	0,00111	A*68:01:02G	C*01:02:01G	B*27:05:02G	DRB1*12:01:01G	DQB1*03:01:01G
618	0,00111	A*68:01:02G	C*04:01:01G	B*35:03:01G	DRB1*04:04:01	DQB1*03:02:01G

619	0,00111	A*68:01:02G	C*04:01:01G	B*35:04:01	DRB1*14:02:01	DQB1*03:01:01G
620	0,00111	A*68:01:02G	C*06:02:01G	B*35:09:01	DRB1*08:04:01	DQB1*03:02:01G
621	0,00111	A*68:01:02G	C*04:01:01G	B*39:03	DRB1*07:01:01G	DQB1*03:02:01G
622	0,00111	A*68:01:02G	C*07:02:01G	B*39:09:01	DRB1*16:02:01G	DQB1*03:01:01G
623	0,00111	A*68:01:02G	C*12:03:01G	B*39:10:01	DRB1*13:02:01G	DQB1*03:02:01G
624	0,00111	A*68:01:02G	C*03:04:01G	B*40:01:01G	DRB1*04:07:01G	DQB1*03:02:01G
625	0,00111	A*68:01:02G	C*03:04:01G	B*40:01:01G	DRB1*15:01:01G	DQB1*03:01:01G
626	0,00111	A*68:01:02G	C*07:02:01G	B*44:02:01G	DRB1*13:01:01G	DQB1*06:03:01G
627	0,00111	A*68:01:02G	C*01:02:01G	B*46:01:01G	DRB1*14:02:01	DQB1*05:02:01G
628	0,00111	A*68:01:02G	C*08:03:01G	B*48:01:01G	DRB1*08:02:01	DQB1*04:02:01G
629	0,00111	A*68:01:02G	C*15:02:01G	B*52:01:02	DRB1*04:11:01	DQB1*03:02:01G
630	0,00111	A*68:01:02G	C*01:02:01G	B*56:01:01G	DRB1*13:02:01G	DQB1*03:02:01G
631	0,00111	A*68:02:01G	C*03:04:01G	B*08:01:01G	DRB1*13:04	DQB1*03:01:01G
632	0,00111	A*68:02:01G	C*02:02:02G	B*14:02:01G	DRB1*13:02:01G	DQB1*04:02:01G
633	0,00111	A*68:02:01G	C*04:01:01G	B*14:02:01G	DRB1*04:04:01	DQB1*03:02:01G
634	0,00111	A*68:02:01G	C*08:02:01G	B*14:02:01G	DRB1*13:03:01	DQB1*03:01:01G
635	0,00111	A*68:02:01G	C*03:04:02	B*15:10:01	DRB1*15:03:01G	DQB1*06:02:01G
636	0,00111	A*68:02:01G	C*05:01:01G	B*18:01:01G	DRB1*03:01:01G	DQB1*03:03:02G
637	0,00111	A*68:02:01G	C*01:02:01G	B*27:05:02G	DRB1*01:01:01G	DQB1*05:01:01G
638	0,00111	A*68:02:01G	C*04:01:01G	B*35:08:01	DRB1*15:01:01G	DQB1*06:02:01G
639	0,00111	A*68:02:01G	C*04:01:01G	B*53:01:01G	DRB1*01:01:01G	DQB1*05:01:01G
640	0,00111	A*68:02:01G	C*04:01:01G	B*53:01:01G	DRB1*01:02:01	DQB1*05:01:01G
641	0,00111	A*68:02:01G	C*16:01:01G	B*57:01:01G	DRB1*01:02:01	DQB1*05:01:01G
642	0,00111	A*68:02:01G	C*07:01:01G	B*57:03:01G	DRB1*09:01:02G	DQB1*03:01:01G
643	0,00111	A*68:17	C*04:01:01G	B*35:05:01	DRB1*04:04:01	DQB1*03:02:01G
644	0,00111	A*68:17	C*07:02:01G	B*35:05:01	DRB1*03:01:01G	DQB1*02:01:01G
645	0,00111	A*68:17	C*04:01:01G	B*35:06	DRB1*04:07:01G	DQB1*03:01:01G

646	0,00111	A*68:17	C*07:02:01G	B*35:06	DRB1*08:07	DQB1*03:01:01G
647	0,00111	A*68:17	C*08:01:01G	B*35:19	DRB1*08:02:01	DQB1*03:02:01G
648	0,00111	A*68:17	C*17:01:01G	B*41:02:01G	DRB1*13:03:01	DQB1*03:01:01G
649	0,00111	A*68:17	C*08:01:01G	B*48:03:01	DRB1*14:06:01	DQB1*03:01:01G
650	0,00111	A*68:17	C*04:01:01G	B*50:01:01G	DRB1*13:03:01	DQB1*03:01:01G
651	0,00111	A*68:23	C*01:02:01G	B*48:01:01G	DRB1*09:01:02G	DQB1*03:03:02G
652	0,00111	A*74:01:01G	C*03:04:01G	B*53:01:01G	DRB1*13:02:01G	DQB1*06:04:01G
653	0,00111	A*80:01:01G	C*17:01:01G	B*41:02:01G	DRB1*12:01:01G	DQB1*03:01:01G
654	0,00111	A*80:01:01G	C*02:02:02G	B*49:01:01G	DRB1*13:01:01G	DQB1*06:03:01G
655	0,00111	A*80:01:01G	C*15:02:01G	B*51:01:01G	DRB1*11:02:01	DQB1*03:01:01G

Supplementary Table 5: Estimated haplotypes and frequencies for SAE group
Five-locus haplotype frequencies (A~C~B~DRB1~DQB1) were estimated for SAE group, using the iterative expectation maximization (EM) algorithm (ε=1e^{-7})

Pos	Freq	Haplotype				
1	0,02047	A*29:02:01G	C*16:01:01G	B*44:03:01G	DRB1*07:01:01G	DQB1*02:01:01G
2	0,01462	A*02:01:01G	C*05:01:01G	B*44:02:01G	DRB1*01:01:01G	DQB1*05:01:01G
3	0,01462	A*02:01:01G	C*15:02:01G	B*51:01:01G	DRB1*11:01:01G	DQB1*03:01:01G
4	0,01462	A*24:02:01G	C*12:03:01G	B*18:01:01G	DRB1*11:04:01G	DQB1*03:01:01G
5	0,01427	A*03:01:01G	C*04:01:01G	B*35:01:01G	DRB1*01:01:01G	DQB1*05:01:01G
6	0,00877	A*01:01:01G	C*07:01:01G	B*08:01:01G	DRB1*03:01:01G	DQB1*02:01:01G
7	0,00877	A*01:01:01G	C*06:02:01G	B*44:03:01G	DRB1*15:01:01G	DQB1*06:02:01G
8	0,00877	A*03:01:01G	C*02:02:02G	B*51:01:01G	DRB1*11:01:01G	DQB1*03:01:01G
9	0,00877	A*11:01:01G	C*04:01:01G	B*35:01:01G	DRB1*01:03	DQB1*05:01:01G
10	0,00877	A*26:01:01G	C*05:01:01G	B*44:02:01G	DRB1*04:02:01	DQB1*03:02:01G
11	0,00877	A*30:01:01G	C*06:02:01G	B*13:02:01G	DRB1*07:01:01G	DQB1*02:01:01G
12	0,00877	A*30:02:01G	C*05:01:01G	B*18:01:01G	DRB1*03:01:01G	DQB1*02:01:01G
13	0,00585	A*01:01:01G	C*08:02:01G	B*14:01:01	DRB1*15:01:01G	DQB1*02:01:01G
14	0,00585	A*01:01:01G	C*07:01:01G	B*15:17:01G	DRB1*11:01:01G	DQB1*03:01:01G
15	0,00585	A*02:01:01G	C*08:02:01G	B*14:02:01G	DRB1*07:01:01G	DQB1*02:01:01G
16	0,00585	A*02:01:01G	C*05:01:01G	B*18:01:01G	DRB1*03:01:01G	DQB1*02:01:01G
17	0,00585	A*02:01:01G	C*16:01:01G	B*44:03:01G	DRB1*15:01:01G	DQB1*06:02:01G
18	0,00585	A*02:01:01G	C*14:02:01G	B*51:01:01G	DRB1*01:01:01G	DQB1*05:01:01G
19	0,00585	A*02:05:01G	C*07:02:01G	B*07:02:01G	DRB1*13:01:01G	DQB1*06:03:01G
20	0,00585	A*03:01:01G	C*07:02:01G	B*07:02:01G	DRB1*15:01:01G	DQB1*06:02:01G
21	0,00585	A*03:01:01G	C*04:01:01G	B*35:02:01G	DRB1*11:04:01G	DQB1*03:01:01G
22	0,00585	A*11:01:01G	C*04:01:01G	B*35:01:01G	DRB1*14:01:01G	DQB1*05:03:01G

23	0,00585	A*24:02:01G	C*07:01:01G	B*07:02:01G	DRB1*15:01:01G	DQB1*06:02:01G
24	0,00585	A*24:02:01G	C*04:01:01G	B*08:01:01G	DRB1*11:04:01G	DQB1*03:01:01G
25	0,00585	A*24:02:01G	C*07:01:01G	B*08:01:01G	DRB1*03:01:01G	DQB1*02:01:01G
26	0,00585	A*24:02:01G	C*06:02:01G	B*13:02:01G	DRB1*13:03:01	DQB1*03:01:01G
27	0,00585	A*24:02:01G	C*03:03:01G	B*15:01:01G	DRB1*11:03:01	DQB1*03:01:01G
28	0,00585	A*24:02:01G	C*12:03:01G	B*18:01:01G	DRB1*03:01:01G	DQB1*02:01:01G
29	0,00585	A*24:02:01G	C*02:02:02G	B*44:03:01G	DRB1*01:02:01	DQB1*05:01:01G
30	0,00585	A*24:02:01G	C*16:01:01G	B*44:03:01G	DRB1*11:01:01G	DQB1*03:01:01G
31	0,00585	A*24:02:01G	C*02:02:02G	B*51:01:01G	DRB1*13:01:01G	DQB1*06:03:01G
32	0,00585	A*25:01:01G	C*12:03:01G	B*18:01:01G	DRB1*01:03	DQB1*05:01:01G
33	0,00585	A*25:01:01G	C*12:03:01G	B*18:01:01G	DRB1*15:01:01G	DQB1*06:02:01G
34	0,00585	A*31:01:02G	C*07:01:01G	B*08:01:01G	DRB1*03:01:01G	DQB1*02:01:01G
35	0,00585	A*31:01:02G	C*03:04:01G	B*40:01:01G	DRB1*04:04:01	DQB1*03:02:01G
36	0,00585	A*32:01:01G	C*08:02:01G	B*14:01:01	DRB1*15:01:01G	DQB1*06:02:01G
37	0,00585	A*32:01:01G	C*12:02:01G	B*52:01:01G	DRB1*15:02:01G	DQB1*06:01:01G
38	0,00585	A*33:01:01G	C*08:02:01G	B*14:02:01G	DRB1*01:02:01	DQB1*05:01:01G
39	0,00585	A*33:01:01G	C*08:02:01G	B*14:02:01G	DRB1*07:01:01G	DQB1*02:01:01G
40	0,00585	A*33:01:01G	C*08:02:01G	B*14:02:01G	DRB1*13:03:01	DQB1*03:01:01G
41	0,00550	A*01:01:01G	C*06:02:01G	B*57:01:01G	DRB1*07:01:01G	DQB1*03:03:02G
42	0,00328	A*01:01:01G	C*04:01:01G	B*35:01:01G	DRB1*01:01:01G	DQB1*05:01:01G
43	0,00328	A*03:01:01G	C*06:02:01G	B*57:01:01G	DRB1*07:01:01G	DQB1*03:03:02G
44	0,00292	A*01:01:01G	C*04:01:01G	B*07:02:01G	DRB1*08:04:01	DQB1*04:02:01G
45	0,00292	A*01:01:01G	C*15:05:01G	B*07:05:01G	DRB1*15:01:01G	DQB1*06:02:01G
46	0,00292	A*01:01:01G	C*05:01:01G	B*15:01:01G	DRB1*08:04:01	DQB1*02:01:01G
47	0,00292	A*01:01:01G	C*07:01:01G	B*18:03	DRB1*07:01:01G	DQB1*03:01:01G
48	0,00292	A*01:01:01G	C*04:01:01G	B*35:08:01	DRB1*04:04:01	DQB1*04:02:01G

49	0,00292	A*01:01:01G	C*04:01:01G	B*35:08:01	DRB1*16:07	DQB1*05:02:01G
50	0,00292	A*01:01:01G	C*06:02:01G	B*37:01:01G	DRB1*01:01:01G	DQB1*05:01:01G
51	0,00292	A*01:01:01G	C*12:03:01G	B*38:01:01	DRB1*04:03:01	DQB1*03:02:01G
52	0,00292	A*01:01:01G	C*07:02:01G	B*39:03	DRB1*07:01:01G	DQB1*02:01:01G
53	0,00292	A*01:01:01G	C*08:01:01G	B*40:01:01G	DRB1*04:03:01	DQB1*03:01:01G
54	0,00292	A*01:01:01G	C*03:04:01G	B*40:04	DRB1*07:01:01G	DQB1*03:03:02G
55	0,00292	A*01:01:01G	C*05:01:01G	B*44:02:01G	DRB1*07:01:01G	DQB1*02:01:01G
56	0,00292	A*01:01:01G	C*16:01:01G	B*44:03:01G	DRB1*04:01:01G	DQB1*03:02:01G
57	0,00292	A*01:01:01G	C*07:01:01G	B*49:01:01G	DRB1*01:03	DQB1*05:01:01G
58	0,00292	A*01:01:01G	C*04:01:01G	B*51:01:01G	DRB1*13:01:01G	DQB1*06:03:01G
59	0,00292	A*01:01:01G	C*16:01:01G	B*51:01:01G	DRB1*04:07:01G	DQB1*06:04:01G
60	0,00292	A*01:01:01G	C*03:03:01G	B*55:01:01G	DRB1*07:01:01G	DQB1*03:03:02G
61	0,00292	A*01:01:01G	C*07:01:01G	B*55:01:01G	DRB1*04:03:01	DQB1*03:01:01G
62	0,00292	A*01:01:01G	C*06:02:01G	B*57:01:01G	DRB1*04:02:01	DQB1*03:02:01G
63	0,00292	A*01:01:01G	C*06:02:01G	B*58:02:01	DRB1*15:01:01G	DQB1*03:01:01G
64	0,00292	A*01:104	C*07:01:01G	B*57:01:01G	DRB1*11:01:01G	DQB1*03:01:01G
65	0,00292	A*02:01:01G	C*07:02:01G	B*07:02:01G	DRB1*01:03	DQB1*05:01:01G
66	0,00292	A*02:01:01G	C*07:02:01G	B*07:02:01G	DRB1*04:02:01	DQB1*03:02:01G
67	0,00292	A*02:01:01G	C*07:02:01G	B*07:02:01G	DRB1*07:01:01G	DQB1*06:02:01G
68	0,00292	A*02:01:01G	C*07:02:01G	B*07:02:01G	DRB1*08:02:01	DQB1*03:02:01G
69	0,00292	A*02:01:01G	C*07:02:01G	B*07:02:01G	DRB1*15:01:01G	DQB1*05:03:01G
70	0,00292	A*02:01:01G	C*06:02:01G	B*08:01:01G	DRB1*11:04:01G	DQB1*02:01:01G
71	0,00292	A*02:01:01G	C*07:01:01G	B*08:01:01G	DRB1*07:01:01G	DQB1*02:01:01G
72	0,00292	A*02:01:01G	C*16:04:01	B*13:02:01G	DRB1*10:01:01G	DQB1*05:01:01G
73	0,00292	A*02:01:01G	C*12:03:01G	B*14:01:01	DRB1*07:01:01G	DQB1*02:01:01G
74	0,00292	A*02:01:01G	C*02:02:02G	B*14:02:01G	DRB1*01:03	DQB1*05:01:01G

75	0,00292	A*02:01:01G	C*06:02:01G	B*15:01:01G	DRB1*07:01:01G	DQB1*03:03:02G
76	0,00292	A*02:01:01G	C*07:02:01G	B*15:01:01G	DRB1*12:01:01G	DQB1*03:01:01G
77	0,00292	A*02:01:01G	C*07:04:01G	B*15:09	DRB1*11:01:01G	DQB1*03:01:01G
78	0,00292	A*02:01:01G	C*06:02:01G	B*18:01:01G	DRB1*04:03:01	DQB1*06:03:01G
79	0,00292	A*02:01:01G	C*07:01:01G	B*18:01:01G	DRB1*11:04:01G	DQB1*03:01:01G
80	0,00292	A*02:01:01G	C*12:03:01G	B*18:01:01G	DRB1*11:04:01G	DQB1*03:01:01G
81	0,00292	A*02:01:01G	C*02:02:02G	B*27:02:01	DRB1*04:01:01G	DQB1*03:02:01G
82	0,00292	A*02:01:01G	C*02:02:02G	B*27:02:01	DRB1*15:01:01G	DQB1*06:02:01G
83	0,00292	A*02:01:01G	C*02:02:02G	B*27:05:02G	DRB1*07:01:01G	DQB1*03:03:02G
84	0,00292	A*02:01:01G	C*02:02:02G	B*27:05:03	DRB1*04:04:01	DQB1*03:02:01G
85	0,00292	A*02:01:01G	C*04:01:01G	B*35:01:01G	DRB1*13:01:01G	DQB1*03:03:02G
86	0,00292	A*02:01:01G	C*04:01:01G	B*35:01:01G	DRB1*16:01:01	DQB1*05:02:01G
87	0,00292	A*02:01:01G	C*06:02:01G	B*35:01:01G	DRB1*13:01:01G	DQB1*06:03:01G
88	0,00292	A*02:01:01G	C*04:01:01G	B*35:02:01G	DRB1*03:01:01G	DQB1*02:01:01G
89	0,00292	A*02:01:01G	C*07:01:01G	B*35:02:01G	DRB1*16:01:01	DQB1*05:02:01G
90	0,00292	A*02:01:01G	C*08:02:01G	B*35:08:01	DRB1*16:01:01	DQB1*05:02:01G
91	0,00292	A*02:01:01G	C*07:02:01G	B*35:09:01	DRB1*04:03:01	DQB1*05:03:01G
92	0,00292	A*02:01:01G	C*12:03:01G	B*38:01:01	DRB1*01:02:01	DQB1*05:03:01G
93	0,00292	A*02:01:01G	C*12:03:01G	B*38:01:01	DRB1*13:01:01G	DQB1*06:03:01G
94	0,00292	A*02:01:01G	C*14:02:01G	B*38:01:01	DRB1*01:03	DQB1*05:01:01G
95	0,00292	A*02:01:01G	C*07:02:01G	B*39:03	DRB1*15:01:01G	DQB1*03:02:01G
96	0,00292	A*02:01:01G	C*07:02:01G	B*39:06:02	DRB1*16:01:01	DQB1*06:04:01G
97	0,00292	A*02:01:01G	C*17:01:01G	B*41:01:01	DRB1*11:02:01	DQB1*03:01:01G
98	0,00292	A*02:01:01G	C*05:01:01G	B*44:02:01G	DRB1*04:01:01G	DQB1*03:01:01G
99	0,00292	A*02:01:01G	C*05:01:01G	B*44:02:01G	DRB1*04:04:01	DQB1*03:02:01G
100	0,00292	A*02:01:01G	C*05:01:01G	B*44:02:01G	DRB1*11:01:01G	DQB1*03:01:01G

101	0,00292	A*02:01:01G	C*07:01:01G	B*44:02:01G	DRB1*03:01:01G	DQB1*03:01:01G
102	0,00292	A*02:01:01G	C*04:01:01G	B*44:03:01G	DRB1*04:02:01	DQB1*03:02:01G
103	0,00292	A*02:01:01G	C*08:01:01G	B*48:01:01G	DRB1*08:02:01	DQB1*04:02:01G
104	0,00292	A*02:01:01G	C*01:02:01G	B*51:01:01G	DRB1*11:01:01G	DQB1*03:01:01G
105	0,00292	A*02:01:01G	C*02:02:02G	B*51:01:01G	DRB1*03:01:01G	DQB1*02:01:01G
106	0,00292	A*02:01:01G	C*02:02:02G	B*51:01:01G	DRB1*13:03:01	DQB1*02:01:01G
107	0,00292	A*02:01:01G	C*07:02:01G	B*51:01:01G	DRB1*01:01:01G	DQB1*05:01:01G
108	0,00292	A*02:01:01G	C*12:03:01G	B*51:01:01G	DRB1*11:01:01G	DQB1*03:01:01G
109	0,00292	A*02:01:01G	C*14:02:01G	B*51:01:01G	DRB1*14:01:01G	DQB1*05:03:01G
110	0,00292	A*02:01:01G	C*15:02:01G	B*51:01:01G	DRB1*04:07:01G	DQB1*03:02:01G
111	0,00292	A*02:01:01G	C*15:02:01G	B*51:01:01G	DRB1*11:04:01G	DQB1*02:01:01G
112	0,00292	A*02:01:01G	C*15:02:01G	B*51:01:01G	DRB1*13:01:01G	DQB1*03:01:01G
113	0,00292	A*02:01:01G	C*16:02:01G	B*51:01:01G	DRB1*03:01:01G	DQB1*03:01:01G
114	0,00292	A*02:01:01G	C*15:02:01G	B*51:08:01	DRB1*04:11:01	DQB1*03:01:01G
115	0,00292	A*02:01:01G	C*04:01:01G	B*53:01:01G	DRB1*13:02:01G	DQB1*06:04:01G
116	0,00292	A*02:01:01G	C*03:03:01G	B*55:01:01G	DRB1*15:01:01G	DQB1*06:02:01G
117	0,00292	A*02:01:01G	C*03:03:01G	B*55:01:01G	DRB1*16:01:01	DQB1*05:02:01G
118	0,00292	A*02:01:01G	C*03:04:01G	B*57:01:01G	DRB1*07:01:01G	DQB1*06:04:01G
119	0,00292	A*02:01:01G	C*07:01:01G	B*57:01:01G	DRB1*13:01:01G	DQB1*03:02:01G
120	0,00292	A*02:01:01G	C*07:01:01G	B*57:03:01G	DRB1*04:11:01	DQB1*03:02:01G
121	0,00292	A*02:01:01G	C*07:01:01G	B*58:01:01G	DRB1*07:01:01G	DQB1*03:01:01G
122	0,00292	A*02:02:01G	C*17:01:01G	B*41:01:01	DRB1*04:05:01	DQB1*02:01:01G
123	0,00292	A*02:04	C*15:02:01G	B*51:01:01G	DRB1*04:11:01	DQB1*04:02:01G
124	0,00292	A*02:05:01G	C*07:01:01G	B*15:17:01G	DRB1*04:07:01G	DQB1*03:01:01G
125	0,00292	A*02:05:01G	C*06:02:01G	B*15:20	DRB1*13:01:01G	DQB1*02:01:01G
126	0,00292	A*02:05:01G	C*03:03:01G	B*50:01:01G	DRB1*09:01:02G	DQB1*02:01:01G

127	0,00292	A*02:06:01G	C*08:03:01G	B*48:01:01G	DRB1*04:03:01	DQB1*03:04:01
128	0,00292	A*02:11:01G	C*03:04:01G	B*48:02:01	DRB1*16:02:01G	DQB1*03:01:01G
129	0,00292	A*02:11:01G	C*04:01:01G	B*48:02:01	DRB1*11:03:01	DQB1*03:01:01G
130	0,00292	A*02:13	C*05:01:01G	B*44:02:01G	DRB1*13:01:01G	DQB1*06:03:01G
131	0,00292	A*03:01:01G	C*04:01:01G	B*07:02:01G	DRB1*14:01:01G	DQB1*03:02:01G
132	0,00292	A*03:01:01G	C*07:02:01G	B*07:02:01G	DRB1*08:01:01G	DQB1*04:02:01G
133	0,00292	A*03:01:01G	C*07:02:01G	B*07:02:01G	DRB1*15:01:01G	DQB1*05:03:01G
134	0,00292	A*03:01:01G	C*12:03:01G	B*07:02:01G	DRB1*13:02:01G	DQB1*05:02:01G
135	0,00292	A*03:01:01G	C*03:04:01G	B*14:01:01	DRB1*04:01:01G	DQB1*03:01:01G
136	0,00292	A*03:01:01G	C*03:04:01G	B*14:02:01G	DRB1*04:07:01G	DQB1*03:02:01G
137	0,00292	A*03:01:01G	C*07:04:01G	B*15:18:01G	DRB1*13:01:01G	DQB1*06:03:01G
138	0,00292	A*03:01:01G	C*07:02:01G	B*18:01:01G	DRB1*13:05:01	DQB1*03:01:01G
139	0,00292	A*03:01:01G	C*07:02:01G	B*27:05:02G	DRB1*11:01:01G	DQB1*03:02:01G
140	0,00292	A*03:01:01G	C*08:02:01G	B*27:05:02G	DRB1*01:01:01G	DQB1*05:01:01G
141	0,00292	A*03:01:01G	C*04:01:01G	B*35:01:01G	DRB1*16:02:01G	DQB1*03:01:01G
142	0,00292	A*03:01:01G	C*12:03:01G	B*35:01:01G	DRB1*13:03:01	DQB1*03:01:01G
143	0,00292	A*03:01:01G	C*04:01:01G	B*35:03:01G	DRB1*11:01:01G	DQB1*03:01:01G
144	0,00292	A*03:01:01G	C*04:01:01G	B*39:14	DRB1*14:02:01	DQB1*03:01:01G
145	0,00292	A*03:01:01G	C*08:02:01G	B*40:01:01G	DRB1*01:01:01G	DQB1*05:01:01G
146	0,00292	A*03:01:01G	C*02:02:02G	B*44:05:01	DRB1*11:01:01G	DQB1*03:01:01G
147	0,00292	A*03:01:01G	C*06:02:01G	B*47:01:01G	DRB1*04:05:01	DQB1*03:02:01G
148	0,00292	A*03:01:01G	C*07:02:01G	B*51:01:01G	DRB1*04:07:01G	DQB1*03:01:01G
149	0,00292	A*03:01:01G	C*14:02:01G	B*51:01:01G	DRB1*07:01:01G	DQB1*02:01:01G
150	0,00292	A*03:01:01G	C*06:02:01G	B*53:01:01G	DRB1*07:01:01G	DQB1*06:03:01G
151	0,00292	A*03:02:01G	C*15:02:01G	B*51:01:01G	DRB1*04:04:01	DQB1*03:02:01G
152	0,00292	A*03:02:01G	C*15:02:01G	B*51:01:01G	DRB1*11:01:01G	DQB1*03:01:01G

153	0,00292	A*11:01:01G	C*04:01:01G	B*14:02:01G	DRB1*01:02:01	DQB1*05:01:01G
154	0,00292	A*11:01:01G	C*02:02:02G	B*27:05:02G	DRB1*14:01:01G	DQB1*06:02:01G
155	0,00292	A*11:01:01G	C*04:01:01G	B*38:01:01	DRB1*13:01:01G	DQB1*02:01:01G
156	0,00292	A*11:01:01G	C*12:03:01G	B*38:01:01	DRB1*07:01:01G	DQB1*05:02:01G
157	0,00292	A*11:01:01G	C*03:03:01G	B*40:01:01G	DRB1*01:01:01G	DQB1*05:01:01G
158	0,00292	A*11:01:01G	C*05:01:01G	B*44:02:01G	DRB1*01:01:01G	DQB1*05:01:01G
159	0,00292	A*11:01:01G	C*16:01:01G	B*44:03:01G	DRB1*11:01:01G	DQB1*03:01:01G
160	0,00292	A*11:01:01G	C*16:01:01G	B*44:03:01G	DRB1*13:01:01G	DQB1*06:03:01G
161	0,00292	A*11:01:01G	C*07:01:01G	B*49:01:01G	DRB1*14:02:01	DQB1*06:09:01G
162	0,00292	A*11:01:01G	C*06:02:01G	B*50:02	DRB1*04:06:01G	DQB1*04:02:01G
163	0,00292	A*11:01:01G	C*02:02:02G	B*51:01:01G	DRB1*04:04:01	DQB1*03:02:01G
164	0,00292	A*11:01:01G	C*03:03:01G	B*51:01:01G	DRB1*09:01:02G	DQB1*03:03:02G
165	0,00292	A*11:01:01G	C*15:02:01G	B*51:01:01G	DRB1*15:01:01G	DQB1*06:02:01G
166	0,00292	A*11:01:01G	C*03:04:01G	B*55:01:01G	DRB1*04:04:01	DQB1*03:02:01G
167	0,00292	A*11:01:01G	C*07:01:01G	B*58:01:01G	DRB1*11:04:01G	DQB1*02:01:01G
168	0,00292	A*23:01:01G	C*07:01:01G	B*18:01:01G	DRB1*11:04:01G	DQB1*03:01:01G
169	0,00292	A*23:01:01G	C*17:01:01G	B*41:01:01	DRB1*01:01:01G	DQB1*06:04:01G
170	0,00292	A*23:01:01G	C*04:01:01G	B*44:03:01G	DRB1*16:01:01	DQB1*02:01:01G
171	0,00292	A*23:01:01G	C*03:03:01G	B*49:01:01G	DRB1*11:04:01G	DQB1*03:02:01G
172	0,00292	A*23:01:01G	C*04:01:01G	B*50:01:01G	DRB1*07:01:01G	DQB1*02:01:01G
173	0,00292	A*24:02:01G	C*02:02:02G	B*07:02:01G	DRB1*04:02:01	DQB1*03:01:01G
174	0,00292	A*24:02:01G	C*07:02:01G	B*07:02:01G	DRB1*04:04:01	DQB1*06:02:01G
175	0,00292	A*24:02:01G	C*08:02:01G	B*14:02:01G	DRB1*01:01:01G	DQB1*05:01:01G
176	0,00292	A*24:02:01G	C*15:02:01G	B*27:05:02G	DRB1*04:01:01G	DQB1*03:01:01G
177	0,00292	A*24:02:01G	C*04:01:01G	B*35:02:01G	DRB1*01:02:01	DQB1*03:01:01G
178	0,00292	A*24:02:01G	C*04:01:01G	B*35:03:01G	DRB1*14:01:01G	DQB1*05:03:01G

179	0,00292	A*24:02:01G	C*04:01:01G	B*38:01:01	DRB1*13:01:01G	DQB1*06:03:01G
180	0,00292	A*24:02:01G	C*12:03:01G	B*38:01:01	DRB1*04:02:01	DQB1*03:02:01G
181	0,00292	A*24:02:01G	C*07:02:01G	B*39:01:01G	DRB1*13:02:01G	DQB1*06:04:01G
182	0,00292	A*24:02:01G	C*12:03:01G	B*39:01:01G	DRB1*08:02:01	DQB1*04:02:01G
183	0,00292	A*24:02:01G	C*07:02:01G	B*39:03	DRB1*14:06:01	DQB1*03:01:01G
184	0,00292	A*24:02:01G	C*16:01:01G	B*39:09:01	DRB1*10:01:01G	DQB1*05:01:01G
185	0,00292	A*24:02:01G	C*02:02:02G	B*40:02:01G	DRB1*04:05:01	DQB1*03:02:01G
186	0,00292	A*24:02:01G	C*03:05	B*40:02:01G	DRB1*04:07:01G	DQB1*03:02:01G
187	0,00292	A*24:02:01G	C*07:02:01G	B*45:01:01G	DRB1*08:02:01	DQB1*03:02:01G
188	0,00292	A*24:02:01G	C*16:01:01G	B*45:01:01G	DRB1*14:01:01G	DQB1*05:03:01G
189	0,00292	A*24:02:01G	C*12:03:01G	B*50:01:01G	DRB1*07:01:01G	DQB1*02:01:01G
190	0,00292	A*24:02:01G	C*01:02:01G	B*51:01:01G	DRB1*07:01:01G	DQB1*03:02:01G
191	0,00292	A*24:02:01G	C*03:04:01G	B*53:01:01G	DRB1*13:02:01G	DQB1*06:04:01G
192	0,00292	A*24:02:01G	C*04:01:01G	B*56:01:01G	DRB1*08:02:01	DQB1*05:01:01G
193	0,00292	A*24:02:01G	C*02:02:02G	B*78:01:01	DRB1*11:03:01	DQB1*05:01:01G
194	0,00292	A*24:03:01G	C*03:03:01G	B*18:01:01G	DRB1*07:01:01G	DQB1*04:02:01G
195	0,00292	A*24:03:01G	C*04:01:01G	B*35:05:01	DRB1*04:04:01	DQB1*03:02:01G
196	0,00292	A*24:03:01G	C*04:01:01G	B*40:02:01G	DRB1*09:01:02G	DQB1*03:03:02G
197	0,00292	A*24:03:01G	C*15:02:01G	B*51:01:01G	DRB1*08:07	DQB1*04:02:01G
198	0,00292	A*24:03:01G	C*03:03:01G	B*55:01:01G	DRB1*14:01:01G	DQB1*05:02:01G
199	0,00292	A*25:01:01G	C*07:02:01G	B*07:02:01G	DRB1*01:01:01G	DQB1*05:01:01G
200	0,00292	A*25:01:01G	C*07:02:01G	B*39:05:01	DRB1*13:01:01G	DQB1*04:02:01G
201	0,00292	A*25:01:01G	C*05:01:01G	B*44:02:01G	DRB1*11:04:01G	DQB1*05:03:01G
202	0,00292	A*25:01:01G	C*03:03:01G	B*55:01:01G	DRB1*09:01:02G	DQB1*05:03:01G
203	0,00292	A*26:01:01G	C*07:02:01G	B*07:02:01G	DRB1*04:01:01G	DQB1*03:01:01G
204	0,00292	A*26:01:01G	C*07:02:01G	B*07:02:01G	DRB1*11:03:01	DQB1*03:01:01G

205	0,00292	A*26:01:01G	C*07:01:01G	B*08:01:01G	DRB1*07:01:01G	DQB1*06:02:01G
206	0,00292	A*26:01:01G	C*01:02:01G	B*14:01:01	DRB1*01:03	DQB1*05:01:01G
207	0,00292	A*26:01:01G	C*07:04:01G	B*15:18:01G	DRB1*13:01:01G	DQB1*06:03:01G
208	0,00292	A*26:01:01G	C*07:01:01G	B*18:01:01G	DRB1*13:05:01	DQB1*03:01:01G
209	0,00292	A*26:01:01G	C*07:02:01G	B*18:01:01G	DRB1*07:01:01G	DQB1*02:01:01G
210	0,00292	A*26:01:01G	C*07:01:01G	B*27:07:01G	DRB1*13:01:01G	DQB1*06:03:01G
211	0,00292	A*26:01:01G	C*04:01:01G	B*35:01:01G	DRB1*13:02:01G	DQB1*03:01:01G
212	0,00292	A*26:01:01G	C*04:01:01G	B*35:20:01	DRB1*08:02:01	DQB1*04:02:01G
213	0,00292	A*26:01:01G	C*12:03:01G	B*38:01:01	DRB1*08:01:01G	DQB1*04:02:01G
214	0,00292	A*26:01:01G	C*15:02:01G	B*51:01:01G	DRB1*11:01:01G	DQB1*03:01:01G
215	0,00292	A*26:01:01G	C*16:02:01G	B*51:01:01G	DRB1*07:01:01G	DQB1*06:03:01G
216	0,00292	A*29:01:01G	C*07:01:01G	B*18:01:01G	DRB1*14:01:01G	DQB1*03:01:01G
217	0,00292	A*29:02:01G	C*07:01:01G	B*08:01:01G	DRB1*15:01:01G	DQB1*05:01:01G
218	0,00292	A*29:02:01G	C*04:01:01G	B*35:01:01G	DRB1*07:01:01G	DQB1*02:01:01G
219	0,00292	A*29:02:01G	C*04:01:01G	B*35:01:01G	DRB1*16:01:01	DQB1*05:02:01G
220	0,00292	A*29:02:01G	C*16:01:01G	B*44:03:01G	DRB1*03:01:01G	DQB1*02:01:01G
221	0,00292	A*29:02:01G	C*16:01:01G	B*44:03:01G	DRB1*11:03:01	DQB1*03:01:01G
222	0,00292	A*29:02:01G	C*15:02:01G	B*51:01:01G	DRB1*04:02:01	DQB1*03:02:01G
223	0,00292	A*30:01:01G	C*07:02:01G	B*07:02:01G	DRB1*07:01:01G	DQB1*03:03:02G
224	0,00292	A*30:01:01G	C*04:01:01G	B*37:01:01G	DRB1*10:01:01G	DQB1*05:01:01G
225	0,00292	A*30:01:01G	C*07:02:01G	B*39:01:01G	DRB1*14:01:01G	DQB1*05:03:01G
226	0,00292	A*30:01:01G	C*02:02:02G	B*44:03:01G	DRB1*13:02:01G	DQB1*05:01:01G
227	0,00292	A*30:01:01G	C*02:02:02G	B*44:05:01	DRB1*01:01:01G	DQB1*05:01:01G
228	0,00292	A*30:02:01G	C*06:02:01G	B*13:02:01G	DRB1*01:01:01G	DQB1*05:01:01G
229	0,00292	A*30:02:01G	C*12:03:01G	B*39:01:01G	DRB1*14:01:01G	DQB1*05:01:01G
230	0,00292	A*30:02:01G	C*07:01:01G	B*49:01:01G	DRB1*13:01:01G	DQB1*03:01:01G

231	0,00292	A*30:04:01G	C*07:01:01G	B*08:01:01G	DRB1*13:03:01	DQB1*06:02:01G
232	0,00292	A*31:01:02G	C*03:03:01G	B*13:02:01G	DRB1*13:01:01G	DQB1*03:01:01G
233	0,00292	A*31:01:02G	C*07:01:01G	B*18:01:01G	DRB1*08:02:01	DQB1*06:03:01G
234	0,00292	A*31:01:02G	C*04:01:01G	B*35:01:01G	DRB1*01:01:01G	DQB1*05:01:01G
235	0,00292	A*31:01:02G	C*04:01:01G	B*35:09:01	DRB1*04:07:01G	DQB1*03:02:01G
236	0,00292	A*31:01:02G	C*04:01:01G	B*35:20:01	DRB1*16:02:01G	DQB1*02:01:01G
237	0,00292	A*31:01:02G	C*07:02:01G	B*39:02:02	DRB1*16:02:01G	DQB1*03:01:01G
238	0,00292	A*31:01:02G	C*07:02:01G	B*39:03	DRB1*04:04:01	DQB1*03:02:01G
239	0,00292	A*31:01:02G	C*07:02:01G	B*39:03	DRB1*13:02:01G	DQB1*03:01:01G
240	0,00292	A*31:01:02G	C*01:02:01G	B*39:05:01	DRB1*04:07:03	DQB1*03:02:01G
241	0,00292	A*31:01:02G	C*15:02:01G	B*40:02:01G	DRB1*16:01:01	DQB1*05:02:01G
242	0,00292	A*31:01:02G	C*15:05:01G	B*40:02:01G	DRB1*01:02:01	DQB1*05:01:01G
243	0,00292	A*31:01:02G	C*04:01:01G	B*44:03:01G	DRB1*04:02:01	DQB1*02:01:01G
244	0,00292	A*31:01:02G	C*01:02:01G	B*49:18:02	DRB1*04:02:01	DQB1*02:01:01G
245	0,00292	A*31:01:02G	C*15:02:01G	B*52:01:02	DRB1*10:01:01G	DQB1*05:01:01G
246	0,00292	A*31:01:02G	C*12:03:01G	B*67:01:02G	DRB1*01:02:01	DQB1*05:01:01G
247	0,00292	A*32:01:01G	C*08:02:01G	B*14:01:01	DRB1*04:05:01	DQB1*03:01:01G
248	0,00292	A*32:01:01G	C*06:02:01G	B*15:01:01G	DRB1*11:01:01G	DQB1*06:03:01G
249	0,00292	A*32:01:01G	C*07:01:01G	B*18:01:01G	DRB1*11:04:01G	DQB1*03:01:01G
250	0,00292	A*32:01:01G	C*07:02:01G	B*18:01:01G	DRB1*04:03:01	DQB1*03:02:01G
251	0,00292	A*32:01:01G	C*06:02:01G	B*35:01:01G	DRB1*13:05:01	DQB1*03:01:01G
252	0,00292	A*32:01:01G	C*07:02:01G	B*39:06:02	DRB1*14:01:01G	DQB1*05:03:01G
253	0,00292	A*32:01:01G	C*02:02:02G	B*40:02:01G	DRB1*07:01:01G	DQB1*02:01:01G
254	0,00292	A*32:01:01G	C*07:01:01G	B*44:03:02G	DRB1*04:01:01G	DQB1*03:02:01G
255	0,00292	A*32:01:01G	C*16:02:01G	B*52:01:02	DRB1*13:03:01	DQB1*03:02:01G
256	0,00292	A*33:01:01G	C*07:02:01G	B*07:02:01G	DRB1*15:01:01G	DQB1*06:03:01G

257	0,00292	A*33:01:01G	C*16:01:01G	B*14:02:01G	DRB1*01:02:01	DQB1*03:01:01G
258	0,00292	A*33:01:01G	C*06:02:01G	B*40:01:01G	DRB1*13:02:01G	DQB1*03:03:02G
259	0,00292	A*33:01:01G	C*14:02:01G	B*51:01:01G	DRB1*13:02:01G	DQB1*06:04:01G
260	0,00292	A*66:01:01G	C*07:01:01G	B*15:01:01G	DRB1*07:01:01G	DQB1*03:02:01G
261	0,00292	A*68:01:01G	C*04:01:01G	B*35:01:01G	DRB1*13:01:01G	DQB1*06:03:01G
262	0,00292	A*68:01:01G	C*04:01:01G	B*35:03:01G	DRB1*04:04:01	DQB1*03:02:01G
263	0,00292	A*68:01:01G	C*04:01:01G	B*35:03:01G	DRB1*15:01:01G	DQB1*06:02:01G
264	0,00292	A*68:01:01G	C*05:01:01G	B*44:02:01G	DRB1*11:04:01G	DQB1*03:01:01G
265	0,00292	A*68:01:02G	C*03:03:01G	B*13:02:01G	DRB1*08:01:01G	DQB1*04:02:01G
266	0,00292	A*68:01:02G	C*07:01:01G	B*18:01:01G	DRB1*04:11:01	DQB1*03:02:01G
267	0,00292	A*68:01:02G	C*01:02:01G	B*35:09:01	DRB1*13:02:01G	DQB1*03:02:01G
268	0,00292	A*68:01:02G	C*03:04:01G	B*40:01:01G	DRB1*01:02:01	DQB1*05:01:01G
269	0,00292	A*68:01:02G	C*03:04:01G	B*40:02:01G	DRB1*14:01:01G	DQB1*03:03:02G
270	0,00292	A*68:01:02G	C*04:01:01G	B*44:03:01G	DRB1*08:02:01	DQB1*03:02:01G
271	0,00292	A*68:02:01G	C*04:01:01G	B*53:01:01G	DRB1*11:04:01G	DQB1*05:01:01G
272	0,00292	A*68:17	C*07:02:01G	B*35:01:01G	DRB1*13:05:01	DQB1*03:01:01G
273	0,00292	A*68:17	C*03:04:01G	B*35:19	DRB1*04:01:01G	DQB1*03:02:01G
274	0,00292	A*68:23	C*01:02:01G	B*48:01:01G	DRB1*09:01:02G	DQB1*03:03:02G
275	0,00292	A*74:01:01G	C*04:01:01G	B*40:01:01G	DRB1*04:05:01	DQB1*03:02:01G
276	0,00292	A*80:01:01G	C*02:02:02G	B*40:02:01G	DRB1*13:03:01	DQB1*06:03:01G
277	0,00292	A*80:01:01G	C*17:01:01G	B*41:02:01G	DRB1*12:01:01G	DQB1*03:01:01G

Supplementary Table 6: Estimated haplotypes and frequencies for SAA group
Five-locus haplotype frequencies (A~C~B~DRB1~DQB1) were estimated for SAA group, using the iterative expectation maximization (EM) algorithm (ε=1e^{-7}).

Pos	Freq	Haplotype				
1	0,27174	A*01:01:01G	C*07:01:01G	B*08:01:01G	DRB1*03:01:01G	DQB1*02:01:01G
2	0,01359	A*03:01:01G	C*07:02:01G	B*07:02:01G	DRB1*15:01:01G	DQB1*06:02:01G
3	0,01359	A*29:02:01G	C*16:01:01G	B*44:03:01G	DRB1*07:01:01G	DQB1*02:01:01G
4	0,01087	A*31:01:02G	C*03:03:01G	B*15:04:01G	DRB1*16:02:01G	DQB1*03:01:01G
5	0,00815	A*02:01:01G	C*05:01:01G	B*44:02:01G	DRB1*07:01:01G	DQB1*02:01:01G
6	0,00815	A*02:01:01G	C*15:02:01G	B*51:01:01G	DRB1*11:02:01	DQB1*03:01:01G
7	0,00815	A*24:03:01G	C*03:04:01G	B*40:02:01G	DRB1*16:02:01G	DQB1*03:01:01G
8	0,00815	A*26:01:01G	C*12:03:01G	B*38:01:01	DRB1*13:01:01G	DQB1*06:03:01G
9	0,00815	A*29:02:01G	C*16:01:01G	B*44:03:01G	DRB1*03:01:01G	DQB1*02:01:01G
10	0,00815	A*30:02:01G	C*05:01:01G	B*18:01:01G	DRB1*03:01:01G	DQB1*02:01:01G
11	0,00815	A*31:01:02G	C*04:01:01G	B*35:04:01	DRB1*08:07	DQB1*04:02:01G
12	0,00815	A*68:01:02G	C*04:01:01G	B*35:05:01	DRB1*09:01:02G	DQB1*03:03:02G
13	0,00544	A*01:01:01G	C*15:02:01G	B*51:01:01G	DRB1*04:02:01	DQB1*03:02:01G
14	0,00544	A*01:01:01G	C*06:02:01G	B*57:01:01G	DRB1*07:01:01G	DQB1*02:01:01G
15	0,00544	A*01:01:01G	C*06:02:01G	B*57:01:01G	DRB1*09:01:02G	DQB1*03:03:02G
16	0,00544	A*02:01:01G	C*07:02:01G	B*07:02:01G	DRB1*15:01:01G	DQB1*06:02:01G
17	0,00544	A*02:01:01G	C*08:02:01G	B*14:02:01G	DRB1*08:02:01	DQB1*04:02:01G
18	0,00544	A*02:01:01G	C*07:01:01G	B*18:01:01G	DRB1*11:04:01G	DQB1*03:01:01G
19	0,00544	A*02:01:01G	C*04:01:01G	B*35:05:01	DRB1*04:04:01	DQB1*03:02:01G
20	0,00544	A*02:01:01G	C*07:02:01G	B*39:05:01	DRB1*04:04:01	DQB1*03:02:01G
21	0,00544	A*02:01:01G	C*03:04:01G	B*40:01:01G	DRB1*04:04:01	DQB1*03:02:01G
22	0,00544	A*02:01:01G	C*02:02:02G	B*40:02:01G	DRB1*11:01:01G	DQB1*03:01:01G
23	0,00544	A*02:01:01G	C*03:04:01G	B*40:04	DRB1*04:11:01	DQB1*03:02:01G
24	0,00544	A*02:01:01G	C*05:01:01G	B*44:02:01G	DRB1*04:01:01G	DQB1*03:01:01G

25	0,00544	A*02:01:01G	C*08:01:01G	B*48:01:01G	DRB1*04:07:01G	DQB1*03:02:01G
26	0,00544	A*02:01:01G	C*07:01:01G	B*51:01:01G	DRB1*13:01:01G	DQB1*03:01:01G
27	0,00544	A*02:01:01G	C*15:02:01G	B*51:01:01G	DRB1*07:01:01G	DQB1*02:01:01G
28	0,00544	A*02:01:01G	C*06:02:01G	B*57:01:01G	DRB1*07:01:01G	DQB1*03:03:02G
29	0,00544	A*02:04	C*15:02:01G	B*51:01:01G	DRB1*07:01:01G	DQB1*04:02:01G
30	0,00544	A*02:11:01G	C*03:04:01G	B*15:01:01G	DRB1*08:04:01	DQB1*03:01:01G
31	0,00544	A*03:01:01G	C*12:03:01G	B*18:01:01G	DRB1*04:03:01	DQB1*03:01:01G
32	0,00544	A*03:01:01G	C*12:03:01G	B*35:03:01G	DRB1*14:01:01G	DQB1*05:03:01G
33	0,00544	A*03:01:01G	C*03:04:01G	B*40:04	DRB1*04:11:01	DQB1*03:02:01G
34	0,00544	A*24:02:01G	C*07:01:01G	B*08:01:01G	DRB1*03:01:01G	DQB1*02:01:01G
35	0,00544	A*24:02:01G	C*04:01:01G	B*35:05:01	DRB1*04:04:01	DQB1*03:02:01G
36	0,00544	A*24:02:01G	C*05:01:01G	B*44:02:01G	DRB1*01:01:01G	DQB1*05:01:01G
37	0,00544	A*24:02:01G	C*04:01:01G	B*51:01:01G	DRB1*11:04:01G	DQB1*03:01:01G
38	0,00544	A*24:02:01G	C*12:02:01G	B*52:01:01G	DRB1*15:02:01G	DQB1*06:01:01G
39	0,00544	A*25:01:01G	C*07:01:01G	B*58:01:01G	DRB1*13:01:01G	DQB1*06:03:01G
40	0,00544	A*29:02:01G	C*16:01:01G	B*44:03:01G	DRB1*13:01:01G	DQB1*06:03:01G
41	0,00544	A*31:01:02G	C*03:03:01G	B*15:01:01G	DRB1*13:01:01G	DQB1*06:03:01G
42	0,00544	A*31:01:02G	C*04:01:01G	B*35:01:01G	DRB1*01:01:01G	DQB1*05:01:01G
43	0,00544	A*32:01:01G	C*04:01:01G	B*35:01:01G	DRB1*11:01:01G	DQB1*03:01:01G
44	0,00544	A*33:01:01G	C*08:02:01G	B*14:02:01G	DRB1*01:02:01	DQB1*05:01:01G
45	0,00544	A*68:01:02G	C*03:04:01G	B*40:04	DRB1*14:02:01	DQB1*03:01:01G
46	0,00272	A*01:01:01G	C*07:01:01G	B*08:01:01G	DRB1*08:01:01G	DQB1*04:02:01G
47	0,00272	A*01:01:01G	C*07:01:01G	B*08:01:01G	DRB1*13:02:01G	DQB1*06:09:01G
48	0,00272	A*01:01:01G	C*07:206	B*08:01:01G	DRB1*11:01:02	DQB1*05:02:01G
49	0,00272	A*01:01:01G	C*06:02:01G	B*13:02:01G	DRB1*11:01:01G	DQB1*03:01:01G
50	0,00272	A*01:01:01G	C*01:02:01G	B*15:01:01G	DRB1*10:01:01G	DQB1*02:01:01G
51	0,00272	A*01:01:01G	C*07:01:01G	B*15:01:01G	DRB1*13:01:01G	DQB1*06:03:01G
52	0,00272	A*01:01:01G	C*05:01:01G	B*18:01:01G	DRB1*07:01:01G	DQB1*03:01:01G

53	0,00272	A*01:01:01G	C*01:02:01G	B*27:05:02G	DRB1*13:01:01G	DQB1*03:01:01G
54	0,00272	A*01:01:01G	C*04:01:01G	B*35:01:01G	DRB1*11:04:01G	DQB1*03:02:01G
55	0,00272	A*01:01:01G	C*06:02:01G	B*37:01:01G	DRB1*11:01:01G	DQB1*03:01:01G
56	0,00272	A*01:01:01G	C*06:02:01G	B*37:01:01G	DRB1*11:03:01	DQB1*03:01:01G
57	0,00272	A*01:01:01G	C*07:01:01G	B*39:05:01	DRB1*13:02:01G	DQB1*06:04:01G
58	0,00272	A*01:01:01G	C*07:02:01G	B*39:06:02	DRB1*13:02:01G	DQB1*06:04:01G
59	0,00272	A*01:01:01G	C*15:02:01G	B*40:06:01G	DRB1*14:04:01	DQB1*05:03:01G
60	0,00272	A*01:01:01G	C*03:04:01G	B*41:01:01	DRB1*03:01:01G	DQB1*03:02:01G
61	0,00272	A*01:01:01G	C*03:04:01G	B*44:02:01G	DRB1*13:01:01G	DQB1*06:03:01G
62	0,00272	A*01:01:01G	C*07:01:01G	B*49:01:01G	DRB1*04:07:03	DQB1*03:01:01G
63	0,00272	A*01:01:01G	C*06:02:01G	B*51:08:01	DRB1*16:01:01	DQB1*03:01:01G
64	0,00272	A*02:01:01G	C*07:01:01G	B*07:02:01G	DRB1*07:01:01G	DQB1*02:01:01G
65	0,00272	A*02:01:01G	C*07:02:01G	B*07:02:01G	DRB1*03:01:01G	DQB1*02:01:01G
66	0,00272	A*02:01:01G	C*07:02:01G	B*07:02:01G	DRB1*08:02:01	DQB1*05:01:01G
67	0,00272	A*02:01:01G	C*08:01:01G	B*07:02:01G	DRB1*04:05:04	DQB1*04:02:01G
68	0,00272	A*02:01:01G	C*05:09:01	B*07:05:01G	DRB1*04:03:01	DQB1*06:03:01G
69	0,00272	A*02:01:01G	C*06:02:01G	B*13:02:01G	DRB1*11:04:01G	DQB1*04:02:01G
70	0,00272	A*02:01:01G	C*03:03:01G	B*15:01:01G	DRB1*13:01:01G	DQB1*05:01:01G
71	0,00272	A*02:01:01G	C*03:04:01G	B*15:01:01G	DRB1*13:02:01G	DQB1*06:04:01G
72	0,00272	A*02:01:01G	C*03:04:02	B*15:01:01G	DRB1*04:01:01G	DQB1*06:02:01G
73	0,00272	A*02:01:01G	C*04:01:01G	B*15:01:01G	DRB1*08:07	DQB1*03:02:01G
74	0,00272	A*02:01:01G	C*16:01:01G	B*15:01:01G	DRB1*04:01:01G	DQB1*05:03:01G
75	0,00272	A*02:01:01G	C*01:02:01G	B*15:04:01G	DRB1*14:02:01	DQB1*03:01:01G
76	0,00272	A*02:01:01G	C*03:03:01G	B*15:04:01G	DRB1*16:02:01G	DQB1*03:02:01G
77	0,00272	A*02:01:01G	C*03:04:01G	B*15:04:01G	DRB1*14:02:01	DQB1*03:01:01G
78	0,00272	A*02:01:01G	C*04:01:01G	B*15:04:01G	DRB1*14:02:01	DQB1*03:01:01G
79	0,00272	A*02:01:01G	C*07:02:01G	B*15:04:01G	DRB1*04:03:01	DQB1*03:02:01G
80	0,00272	A*02:01:01G	C*07:02:01G	B*15:10:01	DRB1*01:02:01	DQB1*05:01:01G

81	0,00272	A*02:01:01G	C*02:02:02G	B*18:01:01G	DRB1*11:01:01G	DQB1*06:03:01G
82	0,00272	A*02:01:01G	C*07:01:01G	B*18:01:01G	DRB1*09:01:02G	DQB1*03:03:02G
83	0,00272	A*02:01:01G	C*02:02:02G	B*27:05:02G	DRB1*04:11:01	DQB1*03:03:02G
84	0,00272	A*02:01:01G	C*04:01:01G	B*35:01:01G	DRB1*11:01:01G	DQB1*06:03:01G
85	0,00272	A*02:01:01G	C*03:03:01G	B*35:02:01G	DRB1*04:01:01G	DQB1*03:02:01G
86	0,00272	A*02:01:01G	C*03:04:01G	B*35:04:01	DRB1*04:11:01	DQB1*03:02:01G
87	0,00272	A*02:01:01G	C*04:01:01G	B*35:04:01	DRB1*14:02:01	DQB1*06:03:01G
88	0,00272	A*02:01:01G	C*15:02:01G	B*35:04:01	DRB1*08:02:01	DQB1*03:01:01G
89	0,00272	A*02:01:01G	C*04:01:01G	B*35:05:01	DRB1*07:01:01G	DQB1*02:01:01G
90	0,00272	A*02:01:01G	C*07:02:01G	B*35:05:01	DRB1*08:02:01	DQB1*05:01:01G
91	0,00272	A*02:01:01G	C*06:02:01G	B*35:08:01	DRB1*11:04:01G	DQB1*03:01:01G
92	0,00272	A*02:01:01G	C*07:01:01G	B*35:20:01	DRB1*03:01:01G	DQB1*02:01:01G
93	0,00272	A*02:01:01G	C*06:02:01G	B*37:01:01G	DRB1*07:01:01G	DQB1*05:01:01G
94	0,00272	A*02:01:01G	C*07:02:01G	B*39:01:01G	DRB1*01:03	DQB1*03:02:01G
95	0,00272	A*02:01:01G	C*07:02:01G	B*39:03	DRB1*04:04:01	DQB1*03:02:01G
96	0,00272	A*02:01:01G	C*07:02:01G	B*39:03	DRB1*09:01:02G	DQB1*03:02:01G
97	0,00272	A*02:01:01G	C*01:02:01G	B*39:05:01	DRB1*09:01:02G	DQB1*03:03:02G
98	0,00272	A*02:01:01G	C*07:02:01G	B*39:05:01	DRB1*11:02:01	DQB1*03:02:01G
99	0,00272	A*02:01:01G	C*07:02:01G	B*39:09:01	DRB1*16:02:01G	DQB1*02:01:01G
100	0,00272	A*02:01:01G	C*03:04:01G	B*40:01:01G	DRB1*07:01:01G	DQB1*02:01:01G
101	0,00272	A*02:01:01G	C*03:04:01G	B*40:02:01G	DRB1*16:02:01G	DQB1*03:01:01G
102	0,00272	A*02:01:01G	C*07:01:01G	B*40:02:01G	DRB1*14:01:01G	DQB1*05:03:01G
103	0,00272	A*02:01:01G	C*05:01:01G	B*40:08	DRB1*16:02:01G	DQB1*03:01:01G
104	0,00272	A*02:01:01G	C*05:01:01G	B*44:02:01G	DRB1*04:02:01	DQB1*03:02:01G
105	0,00272	A*02:01:01G	C*05:01:01G	B*44:02:01G	DRB1*04:04:01	DQB1*03:02:01G
106	0,00272	A*02:01:01G	C*06:02:01G	B*44:02:01G	DRB1*07:01:01G	DQB1*02:01:01G
107	0,00272	A*02:01:01G	C*06:02:01G	B*44:02:01G	DRB1*14:01:01G	DQB1*03:03:02G
108	0,00272	A*02:01:01G	C*04:01:01G	B*44:03:01G	DRB1*16:02:01G	DQB1*03:01:01G

109	0,00272	A*02:01:01G	C*16:01:01G	B*44:03:01G	DRB1*04:04:01	DQB1*02:01:01G
110	0,00272	A*02:01:01G	C*16:01:01G	B*44:03:01G	DRB1*13:01:01G	DQB1*06:03:01G
111	0,00272	A*02:01:01G	C*07:02:01G	B*49:01:01G	DRB1*08:01:01G	DQB1*04:02:03
112	0,00272	A*02:01:01G	C*03:03:01G	B*51:01:01G	DRB1*14:01:01G	DQB1*03:02:01G
113	0,00272	A*02:01:01G	C*12:03:01G	B*51:01:01G	DRB1*11:04:01G	DQB1*03:01:01G
114	0,00272	A*02:01:01G	C*14:02:01G	B*51:01:01G	DRB1*08:01:01G	DQB1*04:02:01G
115	0,00272	A*02:01:01G	C*15:02:01G	B*51:01:01G	DRB1*01:02:01	DQB1*03:02:01G
116	0,00272	A*02:01:01G	C*15:02:01G	B*51:01:01G	DRB1*08:01:01G	DQB1*04:02:01G
117	0,00272	A*02:01:01G	C*15:02:01G	B*51:01:01G	DRB1*11:01:01G	DQB1*06:02:01G
118	0,00272	A*02:01:01G	C*15:03	B*51:04	DRB1*08:04:01	DQB1*04:02:01G
119	0,00272	A*02:01:01G	C*08:02:01G	B*55:01:01G	DRB1*13:02:01G	DQB1*06:02:01G
120	0,00272	A*02:01:01G	C*07:01:01G	B*57:03:01G	DRB1*11:03:01	DQB1*03:01:01G
121	0,00272	A*02:01:01G	C*07:01:01G	B*57:03:01G	DRB1*13:02:01G	DQB1*06:04:01G
122	0,00272	A*02:02:01G	C*06:02:01G	B*15:08:01	DRB1*08:07	DQB1*03:01:01G
123	0,00272	A*02:02:01G	C*17:01:01G	B*39:05:01	DRB1*04:03:01	DQB1*03:02:01G
124	0,00272	A*02:02:01G	C*17:01:01G	B*40:04	DRB1*04:11:01	DQB1*02:01:01G
125	0,00272	A*02:04	C*15:02:01G	B*51:01:01G	DRB1*15:01:01G	DQB1*03:01:01G
126	0,00272	A*02:05:01G	C*06:02:01G	B*50:01:01G	DRB1*09:01:02G	DQB1*03:03:02G
127	0,00272	A*02:05:01G	C*07:01:01G	B*58:01:01G	DRB1*04:01:01G	DQB1*03:02:01G
128	0,00272	A*02:06:01G	C*15:02:01G	B*51:01:01G	DRB1*14:01:01G	DQB1*05:03:01G
129	0,00272	A*02:11:01G	C*12:03:01G	B*39:01:01G	DRB1*13:01:01G	DQB1*06:03:01G
130	0,00272	A*02:11:01G	C*03:04:01G	B*40:04	DRB1*14:02:01	DQB1*03:01:01G
131	0,00272	A*02:11:01G	C*04:01:01G	B*48:02:01	DRB1*04:07:01G	DQB1*03:03:02G
132	0,00272	A*02:11:01G	C*04:01:01G	B*48:02:01	DRB1*09:01:02G	DQB1*03:02:01G
133	0,00272	A*02:11:01G	C*04:01:01G	B*48:02:01	DRB1*16:02:01G	DQB1*04:02:01G
134	0,00272	A*02:11:01G	C*16:01:01G	B*48:02:01	DRB1*04:11:01	DQB1*03:01:01G
135	0,00272	A*02:22:01G	C*04:01:01G	B*08:01:01G	DRB1*01:02:01	DQB1*04:02:01G
136	0,00272	A*02:33	C*04:01:01G	B*35:21	DRB1*16:02:01G	DQB1*03:01:01G

137	0,00272	A*03:01:01G	C*07:02:01G	B*07:02:01G	DRB1*16:01:01	DQB1*05:02:01G
138	0,00272	A*03:01:01G	C*08:02:01G	B*14:02:01G	DRB1*04:04:01	DQB1*05:01:01G
139	0,00272	A*03:01:01G	C*15:02:01G	B*14:02:01G	DRB1*13:02:01G	DQB1*05:03:01G
140	0,00272	A*03:01:01G	C*07:01:01G	B*15:01:01G	DRB1*13:01:01G	DQB1*06:03:01G
141	0,00272	A*03:01:01G	C*07:01:01G	B*18:01:01G	DRB1*11:04:01G	DQB1*03:01:01G
142	0,00272	A*03:01:01G	C*12:03:01G	B*18:01:01G	DRB1*07:01:01G	DQB1*03:03:02G
143	0,00272	A*03:01:01G	C*01:02:01G	B*27:05:02G	DRB1*13:01:01G	DQB1*05:01:01G
144	0,00272	A*03:01:01G	C*04:01:01G	B*35:01:01G	DRB1*01:01:01G	DQB1*05:01:01G
145	0,00272	A*03:01:01G	C*06:02:01G	B*35:02:01G	DRB1*11:04:01G	DQB1*03:01:01G
146	0,00272	A*03:01:01G	C*04:01:01G	B*35:04:01	DRB1*01:02:01	DQB1*05:01:01G
147	0,00272	A*03:01:01G	C*08:02:01G	B*39:05:01	DRB1*04:07:03	DQB1*03:02:01G
148	0,00272	A*03:01:01G	C*07:01:01G	B*40:01:01G	DRB1*03:01:01G	DQB1*02:01:01G
149	0,00272	A*03:01:01G	C*07:04:01G	B*40:01:01G	DRB1*13:02:01G	DQB1*06:04:01G
150	0,00272	A*03:01:01G	C*08:02:01G	B*44:02:01G	DRB1*04:02:01	DQB1*05:01:01G
151	0,00272	A*03:01:01G	C*07:01:01G	B*44:03:01G	DRB1*01:02:01	DQB1*05:01:01G
152	0,00272	A*03:01:01G	C*08:01:01G	B*48:01:01G	DRB1*12:01:01G	DQB1*03:01:01G
153	0,00272	A*03:01:01G	C*03:03:01G	B*51:01:01G	DRB1*11:01:01G	DQB1*03:01:01G
154	0,00272	A*03:01:01G	C*08:02:01G	B*51:01:01G	DRB1*14:01:01G	DQB1*06:09:01G
155	0,00272	A*03:01:01G	C*14:02:01G	B*51:01:01G	DRB1*04:07:01G	DQB1*04:02:01G
156	0,00272	A*03:02:01G	C*16:04:01	B*14:02:01G	DRB1*10:01:01G	DQB1*03:02:01G
157	0,00272	A*03:08	C*07:02:01G	B*08:01:01G	DRB1*04:03:01	DQB1*03:02:01G
158	0,00272	A*11:01:01G	C*07:01:01G	B*08:01:01G	DRB1*03:01:01G	DQB1*02:01:01G
159	0,00272	A*11:01:01G	C*06:02:01G	B*13:02:01G	DRB1*13:01:01G	DQB1*06:03:01G
160	0,00272	A*11:01:01G	C*08:02:01G	B*14:01:01	DRB1*01:03	DQB1*03:02:01G
161	0,00272	A*11:01:01G	C*08:02:01G	B*14:01:01	DRB1*04:11:01	DQB1*02:01:01G
162	0,00272	A*11:01:01G	C*02:02:02G	B*15:01:01G	DRB1*04:01:01G	DQB1*03:04:01
163	0,00272	A*11:01:01G	C*04:01:01G	B*15:01:01G	DRB1*08:02:01	DQB1*05:02:01G
164	0,00272	A*11:01:01G	C*02:02:02G	B*27:05:02G	DRB1*07:01:01G	DQB1*03:03:02G

165	0,00272	A*11:01:01G	C*04:01:01G	B*35:01:01G	DRB1*08:01:01G	DQB1*03:01:01G
166	0,00272	A*11:01:01G	C*04:01:01G	B*35:01:01G	DRB1*15:01:01G	DQB1*05:01:01G
167	0,00272	A*11:01:01G	C*12:03:01G	B*38:01:01	DRB1*01:01:01G	DQB1*06:03:01G
168	0,00272	A*11:01:01G	C*07:02:01G	B*39:05:01	DRB1*04:04:01	DQB1*03:02:01G
169	0,00272	A*11:01:01G	C*07:02:01G	B*39:09:01	DRB1*08:02:01	DQB1*04:02:01G
170	0,00272	A*11:01:01G	C*15:02:01G	B*40:04	DRB1*04:07:01G	DQB1*03:02:01G
171	0,00272	A*11:01:01G	C*05:01:01G	B*44:02:01G	DRB1*04:02:01	DQB1*03:02:01G
172	0,00272	A*11:01:01G	C*07:01:01G	B*44:02:01G	DRB1*14:01:01G	DQB1*05:03:01G
173	0,00272	A*11:01:01G	C*06:02:01G	B*44:03:01G	DRB1*07:01:01G	DQB1*02:01:01G
174	0,00272	A*11:01:01G	C*16:01:01G	B*50:01:01G	DRB1*03:01:01G	DQB1*02:01:01G
175	0,00272	A*11:01:01G	C*12:02:01G	B*51:01:01G	DRB1*04:08:01	DQB1*06:01:01G
176	0,00272	A*11:01:01G	C*15:02:01G	B*51:01:01G	DRB1*13:01:01G	DQB1*03:01:01G
177	0,00272	A*11:01:01G	C*03:04:01G	B*52:01:02	DRB1*15:01:01G	DQB1*06:02:01G
178	0,00272	A*23:01:01G	C*06:02:01G	B*07:02:01G	DRB1*01:02:01	DQB1*05:01:01G
179	0,00272	A*23:01:01G	C*02:10:01G	B*15:03:01G	DRB1*11:01:02	DQB1*03:01:01G
180	0,00272	A*23:01:01G	C*04:01:01G	B*35:01:01G	DRB1*01:01:01G	DQB1*03:01:01G
181	0,00272	A*23:01:01G	C*04:01:01G	B*44:03:01G	DRB1*03:01:01G	DQB1*02:01:01G
182	0,00272	A*23:01:01G	C*15:05:01G	B*45:01:01G	DRB1*04:08:01	DQB1*03:01:01G
183	0,00272	A*23:01:01G	C*06:02:01G	B*53:01:01G	DRB1*08:04:01	DQB1*03:01:01G
184	0,00272	A*23:01:01G	C*07:01:01G	B*58:01:01G	DRB1*04:02:01	DQB1*03:01:01G
185	0,00272	A*24:02:01G	C*08:02:01G	B*14:01:01	DRB1*04:05:01	DQB1*02:01:01G
186	0,00272	A*24:02:01G	C*02:02:02G	B*14:02:01G	DRB1*13:02:01G	DQB1*06:04:01G
187	0,00272	A*24:02:01G	C*03:03:01G	B*15:01:01G	DRB1*12:01:01G	DQB1*06:03:01G
188	0,00272	A*24:02:01G	C*03:03:01G	B*15:04:01G	DRB1*13:01:01G	DQB1*03:03:02G
189	0,00272	A*24:02:01G	C*07:01:01G	B*15:17:01G	DRB1*15:01:01G	DQB1*03:01:01G
190	0,00272	A*24:02:01G	C*07:02:01G	B*15:17:01G	DRB1*04:07:03	DQB1*03:02:01G
191	0,00272	A*24:02:01G	C*07:04:01G	B*18:01:01G	DRB1*04:02:01	DQB1*03:02:01G
192	0,00272	A*24:02:01G	C*02:02:02G	B*27:05:02G	DRB1*08:07	DQB1*04:02:01G

193	0,00272	A*24:02:01G	C*04:01:01G	B*35:02:01G	DRB1*07:01:01G	DQB1*02:01:01G
194	0,00272	A*24:02:01G	C*04:01:01G	B*35:04:01	DRB1*01:01:01G	DQB1*03:01:01G
195	0,00272	A*24:02:01G	C*12:03:01G	B*38:01:01	DRB1*01:01:01G	DQB1*05:01:01G
196	0,00272	A*24:02:01G	C*07:02:01G	B*39:03	DRB1*04:11:01	DQB1*03:02:01G
197	0,00272	A*24:02:01G	C*08:03:01G	B*40:02:01G	DRB1*09:01:02G	DQB1*03:03:02G
198	0,00272	A*24:02:01G	C*03:04:01G	B*40:04	DRB1*04:07:01G	DQB1*03:02:01G
199	0,00272	A*24:02:01G	C*16:02:01G	B*45:01:01G	DRB1*04:01:01G	DQB1*05:02:01G
200	0,00272	A*24:02:01G	C*07:04:01G	B*50:01:01G	DRB1*11:01:01G	DQB1*03:01:01G
201	0,00272	A*24:02:01G	C*15:02:01G	B*51:01:01G	DRB1*04:08:01	DQB1*03:01:01G
202	0,00272	A*24:02:01G	C*06:02:01G	B*57:03:01G	DRB1*09:01:02G	DQB1*03:01:01G
203	0,00272	A*24:03:01G	C*07:02:01G	B*07:02:01G	DRB1*11:04:01G	DQB1*03:01:01G
204	0,00272	A*24:03:01G	C*03:03:01G	B*15:01:01G	DRB1*08:04:01	DQB1*04:02:01G
205	0,00272	A*24:03:01G	C*01:02:01G	B*15:20	DRB1*04:11:01	DQB1*04:02:01G
206	0,00272	A*24:03:01G	C*05:01:01G	B*27:02:01	DRB1*04:07:03	DQB1*03:02:01G
207	0,00272	A*24:03:01G	C*04:01:01G	B*51:13:01	DRB1*14:02:01	DQB1*04:02:01G
208	0,00272	A*24:175	C*07:02:01G	B*39:01:01G	DRB1*14:02:01	DQB1*05:01:01G
209	0,00272	A*25:01:01G	C*12:03:01G	B*35:08:01	DRB1*13:03:01	DQB1*03:01:01G
210	0,00272	A*25:01:01G	C*15:02:01G	B*40:02:01G	DRB1*14:02:01	DQB1*06:02:01G
211	0,00272	A*26:01:01G	C*03:03:01G	B*15:01:01G	DRB1*16:02:01G	DQB1*03:01:01G
212	0,00272	A*26:01:01G	C*04:01:01G	B*18:01:01G	DRB1*07:01:01G	DQB1*02:01:01G
213	0,00272	A*26:01:01G	C*07:02:01G	B*39:13:01	DRB1*03:01:01G	DQB1*03:02:01G
214	0,00272	A*26:01:01G	C*01:02:01G	B*41:01:01	DRB1*11:01:02	DQB1*06:04:01G
215	0,00272	A*26:01:01G	C*14:02:01G	B*51:07:01	DRB1*01:01:01G	DQB1*05:02:01G
216	0,00272	A*29:01:01G	C*15:05:01G	B*07:05:01G	DRB1*09:01:02G	DQB1*03:03:02G
217	0,00272	A*29:02:01G	C*03:04:01G	B*15:15	DRB1*04:04:01	DQB1*03:02:01G
218	0,00272	A*29:02:01G	C*14:02:01G	B*35:05:01	DRB1*14:02:01	DQB1*03:01:01G
219	0,00272	A*29:02:01G	C*03:04:01G	B*40:04	DRB1*11:01:01G	DQB1*03:02:01G
220	0,00272	A*29:02:01G	C*04:01:01G	B*44:03:01G	DRB1*12:01:01G	DQB1*03:02:01G

221	0,00272	A*29:02:01G	C*16:01:01G	B*44:03:01G	DRB1*11:01:01G	DQB1*03:02:01G
222	0,00272	A*29:02:01G	C*06:02:01G	B*45:01:01G	DRB1*11:01:01G	DQB1*05:01:01G
223	0,00272	A*29:02:01G	C*07:01:01G	B*58:01:01G	DRB1*11:01:01G	DQB1*03:01:01G
224	0,00272	A*30:01:01G	C*06:02:01G	B*13:02:01G	DRB1*07:01:01G	DQB1*02:01:01G
225	0,00272	A*30:01:01G	C*06:02:01G	B*50:01:01G	DRB1*03:01:01G	DQB1*02:01:01G
226	0,00272	A*30:01:01G	C*05:01:01G	B*51:01:01G	DRB1*07:01:01G	DQB1*02:01:01G
227	0,00272	A*30:01:01G	C*04:01:01G	B*52:01:01G	DRB1*16:01:01	DQB1*05:02:01G
228	0,00272	A*30:01:01G	C*06:02:01G	B*58:02:01	DRB1*15:03:01G	DQB1*06:02:01G
229	0,00272	A*30:02:01G	C*03:03:01G	B*35:01:01G	DRB1*16:01:01	DQB1*03:02:01G
230	0,00272	A*30:02:01G	C*12:02:01G	B*35:01:01G	DRB1*15:02:01G	DQB1*06:01:01G
231	0,00272	A*30:02:01G	C*03:04:01G	B*40:04	DRB1*13:02:01G	DQB1*06:04:01G
232	0,00272	A*30:02:01G	C*18:01:01G	B*57:03:01G	DRB1*11:01:01G	DQB1*05:01:01G
233	0,00272	A*30:02:01G	C*07:01:01G	B*58:01:01G	DRB1*13:02:01G	DQB1*06:04:01G
234	0,00272	A*31:01:02G	C*03:04:01G	B*08:01:01G	DRB1*04:07:01G	DQB1*03:02:01G
235	0,00272	A*31:01:02G	C*04:01:01G	B*08:01:01G	DRB1*16:02:01G	DQB1*03:01:01G
236	0,00272	A*31:01:02G	C*07:02:01G	B*14:02:01G	DRB1*13:02:01G	DQB1*06:09:01G
237	0,00272	A*31:01:02G	C*03:03:01G	B*15:04:01G	DRB1*09:01:02G	DQB1*03:03:02G
238	0,00272	A*31:01:02G	C*01:02:01G	B*15:07:01G	DRB1*04:04:01	DQB1*03:02:01G
239	0,00272	A*31:01:02G	C*07:01:01G	B*15:17:01G	DRB1*13:02:01G	DQB1*06:04:01G
240	0,00272	A*31:01:02G	C*04:01:01G	B*35:01:01G	DRB1*08:02:01	DQB1*05:01:01G
241	0,00272	A*31:01:02G	C*08:03:01G	B*35:04:01	DRB1*14:02:01	DQB1*03:01:01G
242	0,00272	A*31:01:02G	C*03:04:01G	B*35:06	DRB1*08:07	DQB1*04:02:01G
243	0,00272	A*31:01:02G	C*04:01:01G	B*35:08:01	DRB1*04:03:01	DQB1*03:02:01G
244	0,00272	A*31:01:02G	C*03:04:02	B*39:05:01	DRB1*04:11:01	DQB1*03:02:01G
245	0,00272	A*31:01:02G	C*07:02:01G	B*39:05:01	DRB1*14:02:01	DQB1*03:01:01G
246	0,00272	A*31:01:02G	C*03:03:01G	B*40:01:01G	DRB1*08:02:01	DQB1*04:02:01G
247	0,00272	A*31:01:02G	C*03:04:01G	B*40:01:01G	DRB1*04:04:01	DQB1*05:01:01G
248	0,00272	A*31:01:02G	C*03:03:01G	B*40:02:01G	DRB1*14:02:01	DQB1*03:01:01G

249	0,00272	A*31:01:02G	C*03:04:01G	B*40:02:01G	DRB1*08:02:01	DQB1*03:02:01G
250	0,00272	A*31:01:02G	C*07:02:01G	B*41:01:01	DRB1*11:04:01G	DQB1*03:01:01G
251	0,00272	A*31:01:02G	C*15:05:01G	B*44:02:01G	DRB1*13:01:01G	DQB1*03:02:01G
252	0,00272	A*31:01:02G	C*06:02:01G	B*45:01:01G	DRB1*04:01:01G	DQB1*03:01:01G
253	0,00272	A*31:01:02G	C*07:02:01G	B*58:02:01	DRB1*11:02:01	DQB1*04:02:01G
254	0,00272	A*31:02	C*04:01:01G	B*39:05:01	DRB1*14:02:01	DQB1*03:01:01G
255	0,00272	A*32:01:01G	C*07:02:01G	B*07:02:01G	DRB1*13:01:01G	DQB1*06:03:01G
256	0,00272	A*32:01:01G	C*03:03:01G	B*15:01:01G	DRB1*01:03	DQB1*06:03:01G
257	0,00272	A*32:01:01G	C*16:04:01	B*18:01:01G	DRB1*11:04:01G	DQB1*03:01:01G
258	0,00272	A*32:01:01G	C*02:02:02G	B*40:02:01G	DRB1*14:01:01G	DQB1*05:03:01G
259	0,00272	A*32:01:01G	C*03:04:01G	B*44:02:01G	DRB1*13:02:01G	DQB1*06:04:01G
260	0,00272	A*32:01:01G	C*15:02:01G	B*52:01:01G	DRB1*15:02:01G	DQB1*03:01:01G
261	0,00272	A*33:01:01G	C*03:03:01G	B*51:01:01G	DRB1*09:01:02G	DQB1*02:01:01G
262	0,00272	A*33:01:01G	C*02:02:02G	B*51:13:01	DRB1*08:02:01	DQB1*04:02:01G
263	0,00272	A*33:03:01G	C*05:01:01G	B*18:01:01G	DRB1*04:07:01G	DQB1*03:02:01G
264	0,00272	A*33:03:01G	C*04:01:01G	B*39:10:01	DRB1*13:02:01G	DQB1*03:02:01G
265	0,00272	A*33:03:01G	C*03:02:01G	B*58:01:01G	DRB1*11:02:01	DQB1*03:01:01G
266	0,00272	A*33:05	C*08:02:01G	B*14:02:01G	DRB1*07:01:01G	DQB1*03:03:02G
267	0,00272	A*34:02:01	C*06:02:01G	B*35:01:01G	DRB1*04:01:01G	DQB1*03:02:01G
268	0,00272	A*66:01:01G	C*17:01:01G	B*41:02:01G	DRB1*13:03:01	DQB1*03:01:01G
269	0,00272	A*66:01:01G	C*07:04:01G	B*58:02:01	DRB1*13:01:01G	DQB1*05:03:01G
270	0,00272	A*68:01:01G	C*02:14:01G	B*81:01:01G	DRB1*13:01:01G	DQB1*05:01:01G
271	0,00272	A*68:01:02G	C*07:02:01G	B*08:01:01G	DRB1*04:03:01	DQB1*03:02:01G
272	0,00272	A*68:01:02G	C*01:02:01G	B*27:05:02G	DRB1*12:01:01G	DQB1*03:01:01G
273	0,00272	A*68:01:02G	C*04:01:01G	B*35:01:01G	DRB1*04:07:01G	DQB1*03:02:01G
274	0,00272	A*68:01:02G	C*04:01:01G	B*35:04:01	DRB1*14:02:01	DQB1*03:01:01G
275	0,00272	A*68:01:02G	C*12:03:01G	B*35:08:01	DRB1*04:07:01G	DQB1*05:01:01G
276	0,00272	A*68:01:02G	C*04:01:01G	B*35:09:01	DRB1*08:02:01	DQB1*03:02:01G

277	0,00272	A*68:01:02G	C*07:02:01G	B*39:05:01	DRB1*09:01:02G	DQB1*03:03:02G
278	0,00272	A*68:01:02G	C*15:02:01G	B*40:01:01G	DRB1*14:02:01	DQB1*03:01:01G
279	0,00272	A*68:01:02G	C*07:04:01G	B*44:02:01G	DRB1*13:01:01G	DQB1*06:03:01G
280	0,00272	A*68:01:02G	C*16:01:01G	B*44:04	DRB1*04:11:01	DQB1*03:01:01G
281	0,00272	A*68:01:02G	C*02:02:02G	B*48:01:01G	DRB1*15:01:01G	DQB1*06:02:01G
282	0,00272	A*68:01:02G	C*08:03:01G	B*48:01:01G	DRB1*08:02:01	DQB1*04:02:01G
283	0,00272	A*68:01:02G	C*03:04:01G	B*51:01:01G	DRB1*15:01:01G	DQB1*06:02:01G
284	0,00272	A*68:01:02G	C*15:02:01G	B*52:01:02	DRB1*04:04:01	DQB1*03:02:01G
285	0,00272	A*68:02:01G	C*03:04:01G	B*15:10:01	DRB1*15:03:01G	DQB1*03:02:01G
286	0,00272	A*68:02:01G	C*04:01:01G	B*35:01:01G	DRB1*01:02:01	DQB1*03:02:01G
287	0,00272	A*68:02:01G	C*04:01:01G	B*35:08:01	DRB1*15:01:01G	DQB1*06:02:01G
288	0,00272	A*68:02:01G	C*04:01:01G	B*53:01:01G	DRB1*01:01:01G	DQB1*05:01:01G
289	0,00272	A*68:02:01G	C*04:01:01G	B*53:01:01G	DRB1*08:07	DQB1*04:02:01G
290	0,00272	A*68:02:01G	C*07:01:01G	B*53:01:01G	DRB1*13:03:01	DQB1*02:01:01G
291	0,00272	A*68:02:01G	C*16:01:01G	B*57:01:01G	DRB1*07:01:01G	DQB1*03:03:02G
292	0,00272	A*68:17	C*04:01:01G	B*35:05:01	DRB1*08:02:01	DQB1*04:02:01G
293	0,00272	A*68:17	C*08:01:01G	B*35:19	DRB1*04:03:01	DQB1*03:02:01G
294	0,00272	A*68:17	C*08:01:01G	B*35:19	DRB1*08:02:01	DQB1*03:02:01G
295	0,00272	A*68:17	C*04:01:01G	B*35:21	DRB1*04:04:01	DQB1*03:02:01G
296	0,00272	A*68:17	C*04:01:01G	B*35:21	DRB1*08:02:01	DQB1*03:01:01G
297	0,00272	A*68:17	C*07:02:01G	B*39:13:01	DRB1*16:02:01G	DQB1*03:01:01G
298	0,00272	A*68:17	C*07:02:01G	B*39:14	DRB1*14:02:01	DQB1*04:02:01G
299	0,00272	A*80:01:01G	C*17:01:01G	B*41:02:01G	DRB1*13:03:01	DQB1*03:01:01G

Supplementary Table 7: UCB units containing the LD for HLA-DRB1~HLA-DQB1.
Full genotypes of UCB units in SAE group containing the LD for DRB1*15:01:01G~ DQB1*02:01:01G.

UCB	HLA-A		HLA-B		HLA-C		HLA-DRB1		HLA-DQB1	
1	01:01:01G	23:01:01G	44:03:01G	50:01:01G	04:01:01G	06:02:01G	07:01:01G	15:01:01G	02:01:01G	06:02:01G
2	01:01:01G	26:01:01G	08:01:01G	14:01:01	07:01:01G	08:02:01G	07:01:01G	15:01:01G	02:01:01G	06:02:01G
3	01:01:01G	29:02:01G	07:05:01G	44:03:01G	15:05:01G	16:01:01G	07:01:01G	15:01:01G	02:01:01G	06:02:01G
4	24:02:01G	26:01:01G	07:02:01G	18:01:01G	07:01:01G	07:02:01G	07:01:01G	15:01:01G	02:01:01G	06:02:01G
5	01:01:01G	02:01:01G	08:01:01G	44:03:01G	06:02:01G	07:01:01G	07:01:01G	15:01:01G	02:01:01G	06:02:01G
6	03:01:01G	30:02:01G	07:02:01G	18:01:01G	05:01:01G	07:02:01G	03:01:01G	15:01:01G	02:01:01G	06:02:01G
7	01:01:01G	02:01:01G	07:02:01G	14:01:01	07:02:01G	08:02:01G	07:01:01G	15:01:01G	02:01:01G	06:02:01G
8	01:01:01G	02:01:01G	44:02:01G	44:03:01G	05:01:01G	16:01:01G	07:01:01G	15:01:01G	02:01:01G	06:02:01G
